# MyD88 in myeloid cells drives angiotensin II-induced vascular inflammation, is associated with prevalent heart failure, and predicts all-cause mortality in arterial hypertension

**DOI:** 10.1093/ehjopen/oeag031

**Published:** 2026-02-27

**Authors:** Sabine Wild, Stefanie Finger, Andreas Schulz, Melania Aluia, Jimena Bravo, Rahul Kumar, Jeremy Lagrange, Gregorio Alanis-Lobato, Voahanginirina Randriamboavonjy, Johannes Wild, Michael Molitor, Christian Müller, Tanja Zeller, Alexander Gieswinkel, Andreas Daiber, Susanne Helena Karbach, Marcus Dörr, Ingrid Fleming, Philipp Lurz, Thomas Münzel, Markus Radsak, Katrin Schäfer, Daniela S Krause, Miguel A Andrade-Navarro, Philipp Wild, Philip Wenzel

**Affiliations:** Center for Cardiology, University Medical Center Mainz, Langenbeckstrasse 1, Mainz 55131, Germany; Center for Thrombosis and Hemostasis Mainz, University Medical Center Mainz, Langenbeckstrasse 1, Mainz 55131, Germany; Center for Cardiology, University Medical Center Mainz, Langenbeckstrasse 1, Mainz 55131, Germany; Center for Thrombosis and Hemostasis Mainz, University Medical Center Mainz, Langenbeckstrasse 1, Mainz 55131, Germany; German Center for Cardiovascular Research (DZHK)—Partner Site Rhine-Main, Germany; Preventive Cardiology and Preventive Medicine, Center for Cardiology, University Medical Center Mainz, Langenbeckstrasse 1, Mainz 55131, Germany; Center for Thrombosis and Hemostasis Mainz, University Medical Center Mainz, Langenbeckstrasse 1, Mainz 55131, Germany; German Center for Cardiovascular Research (DZHK)—Partner Site Rhine-Main, Germany; Institute of Transfusion-Medicine Center, University Medical Center Mainz, Langenbeckstrasse 1, Mainz 55131, Germany; Institute of Transfusion-Medicine Center, University Medical Center Mainz, Langenbeckstrasse 1, Mainz 55131, Germany; Center for Thrombosis and Hemostasis Mainz, University Medical Center Mainz, Langenbeckstrasse 1, Mainz 55131, Germany; INSERM, DCAC, Université de Lorraine, Nancy F-54000, France; Inserm, IHU INFINY, Université de Lorraine, CHRU-Nancy, Nancy F-54000, France; Faculty of Biology, Johannes Gutenberg University Mainz, Gresemundweg 2, Mainz 55128, Germany; Institute for Molecular Biology GGmbH, Ackermannweg 4, Mainz 55128, Germany; German Center for Cardiovascular Research (DZHK)—Partner Site Rhine-Main, Germany; Institute for Vascular Signaling, Centre for Molecular Medicine, Goethe University, Theodor-Stern-Kai 7, Frankfurt am Main 60596, Germany; Center for Cardiology, University Medical Center Mainz, Langenbeckstrasse 1, Mainz 55131, Germany; Center for Thrombosis and Hemostasis Mainz, University Medical Center Mainz, Langenbeckstrasse 1, Mainz 55131, Germany; German Center for Cardiovascular Research (DZHK)—Partner Site Rhine-Main, Germany; Center for Cardiology, University Medical Center Mainz, Langenbeckstrasse 1, Mainz 55131, Germany; Center for Thrombosis and Hemostasis Mainz, University Medical Center Mainz, Langenbeckstrasse 1, Mainz 55131, Germany; German Center for Cardiovascular Research (DZHK)—Partner Site Rhine-Main, Germany; Institute for Cardiogenetics, University Hospital Schleswig Holstein, University of Luebeck, Ratzeburger Allee 160, Lübeck 23562 Germany; German Center for Cardiovascular Research (DZHK), Partner Site Hamburg/Kiel/Luebeck, Germany; Institute for Cardiogenetics, University Hospital Schleswig Holstein, University of Luebeck, Ratzeburger Allee 160, Lübeck 23562 Germany; German Center for Cardiovascular Research (DZHK), Partner Site Hamburg/Kiel/Luebeck, Germany; German Center for Cardiovascular Research (DZHK)—Partner Site Rhine-Main, Germany; Preventive Cardiology and Preventive Medicine, Center for Cardiology, University Medical Center Mainz, Langenbeckstrasse 1, Mainz 55131, Germany; Institute of Mathematics, Johannes Gutenberg University Mainz, Staudingerweg 9, Mainz 55128, Germany; Center for Cardiology, University Medical Center Mainz, Langenbeckstrasse 1, Mainz 55131, Germany; German Center for Cardiovascular Research (DZHK)—Partner Site Rhine-Main, Germany; Center for Cardiology, University Medical Center Mainz, Langenbeckstrasse 1, Mainz 55131, Germany; Center for Thrombosis and Hemostasis Mainz, University Medical Center Mainz, Langenbeckstrasse 1, Mainz 55131, Germany; German Center for Cardiovascular Research (DZHK)—Partner Site Rhine-Main, Germany; Institute for Community Medicine, University Medicine Greifswald, Walther-Rathenau-Str. 48, 17475 Greifswald, Germany; German Center for Cardiovascular Research (DZHK), Partner Site Greifswald, Germany; Department of Internal Medicine B, University Medicine Greifswald, Ferdinand-Sauerbruch-Straße -17475 Greifswald, Germany; German Center for Cardiovascular Research (DZHK)—Partner Site Rhine-Main, Germany; Institute for Vascular Signaling, Centre for Molecular Medicine, Goethe University, Theodor-Stern-Kai 7, Frankfurt am Main 60596, Germany; Center for Cardiology, University Medical Center Mainz, Langenbeckstrasse 1, Mainz 55131, Germany; German Center for Cardiovascular Research (DZHK)—Partner Site Rhine-Main, Germany; Center for Cardiology, University Medical Center Mainz, Langenbeckstrasse 1, Mainz 55131, Germany; Center for Thrombosis and Hemostasis Mainz, University Medical Center Mainz, Langenbeckstrasse 1, Mainz 55131, Germany; German Center for Cardiovascular Research (DZHK)—Partner Site Rhine-Main, Germany; IIIrd Medical Clinic, University Medical Center Mainz, Langenbeckstrasse 1, Mainz 55131, Germany; Center for Cardiology, University Medical Center Mainz, Langenbeckstrasse 1, Mainz 55131, Germany; German Center for Cardiovascular Research (DZHK)—Partner Site Rhine-Main, Germany; Institute of Transfusion-Medicine Center, University Medical Center Mainz, Langenbeckstrasse 1, Mainz 55131, Germany; Research Center for Immunotherapy (FZI), University Medical Center, University of Mainz, Langenbeckstr. 1, 55131 Mainz, Germany; German Cancer Research Center (DKFZ), Im Neuenheimer Feld 280, 69120 Heidelberg, Germany; German Cancer Consortium (DKTK), Germany; Faculty of Biology, Johannes Gutenberg University Mainz, Gresemundweg 2, Mainz 55128, Germany; Institute for Molecular Biology GGmbH, Ackermannweg 4, Mainz 55128, Germany; Center for Thrombosis and Hemostasis Mainz, University Medical Center Mainz, Langenbeckstrasse 1, Mainz 55131, Germany; German Center for Cardiovascular Research (DZHK)—Partner Site Rhine-Main, Germany; Preventive Cardiology and Preventive Medicine, Center for Cardiology, University Medical Center Mainz, Langenbeckstrasse 1, Mainz 55131, Germany; Center for Cardiology, University Medical Center Mainz, Langenbeckstrasse 1, Mainz 55131, Germany; Center for Thrombosis and Hemostasis Mainz, University Medical Center Mainz, Langenbeckstrasse 1, Mainz 55131, Germany; German Center for Cardiovascular Research (DZHK)—Partner Site Rhine-Main, Germany; Medical Clinic I - Cardiology and Internal Intensive Care Medicine, Klinikum Darmstadt GmbH, Grafenstrasse 9, Darmstadt 64283

**Keywords:** Arterial hypertension, Heart Failure, Myeloid cells, MyD88

## Abstract

**Aims:**

Angiotensin II (AngII) causes hypertension and vascular inflammation and is essential in neurohumoral activation promoting the development of heart failure. The role of the adaptor protein myeloid factor of differentiation 88 (MyD88) driving this pathology remains incompletely understood.

**Methods and results:**

Male C57BL/6JMyD88^−/−^, LysM^Cre/wt^MyD88^LSL/LSL^, LysM^Cre/wt^, TLR2^−/−^, TLR4^−/−^, TLR7^−/−^, and TLR9^−/−^ mice were investigated (1 mg/AngIIkg/d for 7 days). Additionally, we performed biodata analyses from a population-based cohort study and human protein network interactome analyses to understand the role of MyD88 in hypertension. MyD88 deficiency attenuated AngII-induced hypertension and endothelial dysfunction in conductance and resistance vessels, surpassing the effect of single TLR deficiencies. Vascular mRNA expression levels of *vcam-1*, *nos2*, *nox2*, *cd62L*, *cd68*, *ccl2*, *il12*, and *il1b* and accumulation of CD11b^+^Ly6C^hi^ inflammatory monocytes and interferon-g^+^ NK cells were significantly dampened in MyD88^−/−^. Vascular protection was conferred by MyD88 deficiency in bone marrow–derived cells. Re-expression of MyD88 in LysM^Cre/wt^MyD88^LSL/LSL^ mice restored AngII-induced pathology, revealing that myeloid cells drive vascular dysfunction in a MyD88-dependent manner. Computational analyses of the human protein interactome demonstrated that MyD88 expression significantly associates with proteins encoded by genetic loci associated with blood pressure traits in multiple GWAS. In hypertensive individuals of the Gutenberg Health Study, monocytic MyD88 mRNA expression was associated with prevalent heart failure and all-cause mortality after a median follow-up of 16.5 years.

**Conclusion:**

MyD88 promotes AngII-induced vascular dysfunction and arterial hypertension and might serve as both an inflammatory diagnostic marker and a drug target to tackle the risk of death and incident heart failure in hypertensive patients.

## Graphical Abstract

**Graphical Abstract:**
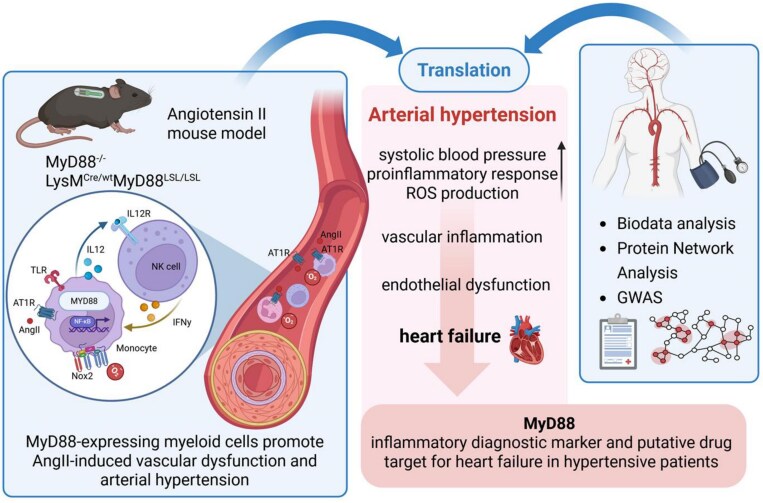

Translational perspective Inflammation is a pathophysiological driver of hypertension and heart failure, but mechanisms are multifactorial and incompletely understood. Furthermore, inflammatory pathways that would work both for risk stratification and as potential therapeutic targets are not well established. Here, we decipher the role of myeloid factor of differentiation 88 (MyD88) expressed by myeloid cells as a driving force for the development of vascular dysfunction and inflammation in experimental hypertension. In various genetic mouse models, we establish that this pathophysiology is not dependent on a single toll-like receptor signalling pathway, but rather intrinsic to bone marrow–derived myeloid cells. MyD88 determines their capability to infiltrate and accumulate in the vasculature, to transdifferentiate into vascular BM derived macrophages, and to promote vascular inflammation and remodelling in hypertension. In individuals with hypertension of the population-based cohort study, MyD88 expression in monocytes highly correlates with all-cause mortality and prevalent heart failure and by trend also predicts incident heart failure at follow-up. Collectively, these findings suggest that MyD88 may work as a biomarker for risk stratification in individuals with hypertension and that it may serve as therapeutic target to treat or prevent heart failure as a sequel of hypertension.

## Introduction

Adaptive and innate immunity have been implicated in endothelial dysfunction, as well as in the development and perpetuation of arterial hypertension and hypertensive end-organ damage. RAG-1^−/−^ mice lack lymphocytes and have diminished hypertensive responses to both angiotensin II (AngII) infusion and deoxycorticosterone acetate (DOCA)-salt challenge. Adoptive transfer of T cells completely restored hypertension in these animals,^1^ highlighting that T-cell co-stimulation by B7 ligands is an important mechanism in the development of hypertension.^2^ As cells of the innate immune response precede T cell-mediated immunity, we and others addressed the role of myeloid cells in the aetiology of hypertension. T-cell activation is driven by isoketal protein adducts accumulated in dendritic cells,^3^ which are preferably monocyte-derived.^4^ Accordingly, mice with macrophage colony stimulating factor deficiency (op/op mice) limit AngII-induced vascular dysfunction and hypertension.^5^ Ablation of myelomonocytic cells from the circulation of LysM^iDTR^ mice attenuated AngII-induced arterial hypertension, vascular dysfunction, and oxidative stress, and monocyte reconstitution re-established the pathophysiological response to AngII in depleted mice.^6^ AngII, the major effector peptide of the renin–angiotensin system (RAS), plays a central role in regulating blood pressure and electrolyte homeostasis and is involved in the key events of vascular inflammation.^7,8^ It increases vascular permeability and contributes to the recruitment of inflammatory cells into tissue through the regulation of cell adhesion molecules (CAMs) and their ligands^9^ and by stimulating the production of cytokines and chemokines in resident vascular and renal cells. AngII directly activates infiltrating immune-competent cells, including their chemotaxis, differentiation, and proliferation.^10^

Myeloid factor of differentiation 88 (MyD88) is crucial for the innate immune response. Originally, MyD88 was identified as a myeloid differentiation primary response gene activated in murine M1 myeloid precursors following IL-6-induced terminal differentiation.^11^ This immediate–early activation profile suggested that MyD88 functions in the regulated progression of myeloid differentiation.^12^ Long-term survival of macrophages is MyD88-dependent and requires autocrine signalling via tumour necrosis factor alpha (TNF-α).^13^ Macrophages from MyD88-deficient mice fail to produce any detectable levels of the inflammatory cytokines TNF-α, IL-12, IL-1, and IL-6 in response to several ligands,^14–16^ and MyD88^−/−^ mice are partially protected from atherosclerosis via decreased macrophage recruitment to the arterial wall paralleled by reduced chemokine levels.^17,18^

Sequence variation studies in healthy individuals have shown that MyD88 has evolved under purifying selection, indicating that deleterious single nucleotide polymorphisms (SNPs) within these genes would impact survival.^19^ In fact, MyD88-deficient humans suffer from severe pyogenic bacterial infections,^20^ and gain-of-function mutations are associated with malignancies.^21,22^ Several reports have linked increased MyD88 expression levels in peripheral blood mononuclear cells (PBMCs) with obesity,^23^ acute myocardial infarction,^24^ unstable angina,^25^ as well as diabetes type 2.^26^ It is unclear if MyD88 contributes to the regulation of vascular tone, AngII-induced inflammation, and arterial hypertension *in vivo*. Furthermore, it is unknown if there is a link between MyD88 expression and hypertension—a major risk factor for mortality and for the development of heart failure—in humans. We hypothesized that MyD88 facilitates vascular accumulation of innate immune cells and a pro-inflammatory phenotype of myelomonocytic cells in response to AngII, impacting the development of heart failure and mortality in humans with hypertension.

## Material and methods

Data, analytical methods, and study material are made available to other researchers for purposes of reproducing the results or replicating the procedure upon request to the corresponding author.

All animal experiments were carried out in accordance with the guide for the care and use of laboratory animals and approved by the ‘Landesuntersuchungsamt Rheinland-Pfalz’ (approval numbers A23-177-07/G11-1-018, G12-1-002, G18-1-080). Mice were bred in the Translational Animal Research Center of the University Medical Center Mainz. MyD88^−/−^ were also commercially available (JAX, Bar Harbor, Maine, USA).

Male C57BL/6J mice as well as MyD88^−/−^, LysM^Cre/wt^MyD88^LSL/LSL^, LysM^Cre/wt^, TLR2^−/−^, TLR4^−/−^, TLR7^−/−^, and TLR9^−/−^ were investigated in the AngII-model of hypertension (1 mg/kg/d for 7 days). Blood pressure was recorded telemetrically and by tail-cuff. Vascular endothelial function of aortas and mesenteric arteries was assessed analysing concentration–relaxation curves of isolated vessel rings mounted on force transducers (organ bath). Vascular immune cell accumulation was analysed using flow cytometry and immunohistochemistry. Nitric oxide (NO) was measured by electron paramagnetic resonance spectroscopy. Reactive oxygen formation was measured by oxidative fluorescent microtopography. mRNA expression analysis was performed by real-time PCR. Protein expression was analysed by Western blot. Data were analysed for normal distribution with the Kolmogorov–Smirnov test. When a normal distribution was given, the one-way ANOVA test with Bonferroni or Tukey’s *post hoc* test or two-way-ANOVA was applied. If the distribution was not normal, Kruskal–Wallis test with Dunn’s multiple comparison or comparison of selected columns was used as appropriate. Biodata was analysed in the Gutenberg Health Study (GHS), a prospective, population-based cohort study (https://pubmed.ncbi.nlm.nih.gov/22736163/). Linear, robust Poisson, and Cox regression models as well as Kaplan–Meier analyses for cumulative incidence were conducted. We performed computational analyses of known candidate genes from genome-wide association studies to investigate the human protein interaction network of MyD88 in hypertension. A detailed material and methods section can be found in the Supplementary material.

## Results

### MyD88 deficiency attenuates angiotensin II-induced endothelial dysfunction and vascular inflammation

**Figure 1:**
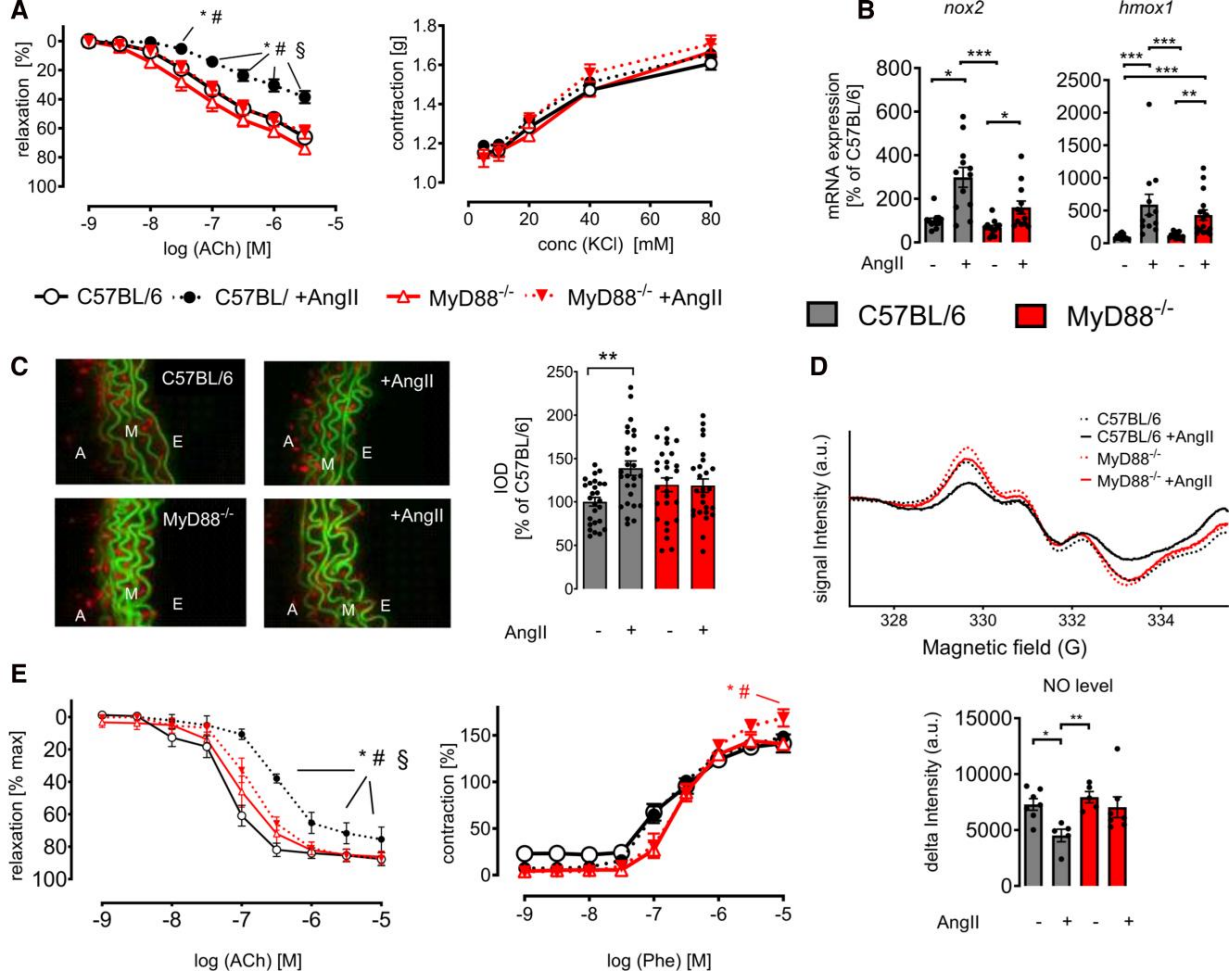
MyD88 deficiency attenuates AngII-induced vascular dysfunction and arterial hypertension. (*A*) Cumulative concentration–relaxation (left) or concentration–restriction curves (right) of isolated aortic rings from sham-treated and AngII-treated C57BL/6 and MyD88^−/−^ mice in response to endothelium dependent vasodilator acetylcholine (ACh) or the vasoconstrictor potassium chloride (KCl). Data are mean ± SEM of *n* = 6–11 animals per group; two-way ANOVA; *P* < 0.05; *, vs. C57BL/6; #, vs. MyD88^−/−^; §, vs. MyD88^−/−^+AngII). (*B*) mRNA expression of the phagocytic NADPH oxidase (*nox2*) and of heme oxygenase-1 (*hmox1*) was assessed by qRT-PCR. Ordinary one-way ANOVA, data are displayed as percentage of C57BL/6 controls; *n* = 12. Data are mean ± SEM; ***P < 0.001; **P < 0.01; *P < 0.05. (*C*) Oxidative fluorescence microtopography. Left: Representative photomicrographs of isolated aortic segments incubated with DHE (1 mM, 30 min at 37°C). Green, laminae (autofluorescence); red fluorescence, superoxide formation; E, endothelium; M, media; A, adventitia. Right: Densitometric analysis, integrated optical density (IOD). *P < 0.05; **P < 0.01; ***P < 0.001, one-way ANOVA and Bonferroni’s multiple comparison test; *n* = 5 independent experiments. Data are mean ± SEM. (*D*) Quantification of the NO signal detected by electron paramagnetic resonance Fe(DECT)2 spin trapping. Top: Mean NO traces of mice. Bottom: Quantification of the signal intensities. Data are mean ± SEM; Kruskal–Wallis test; ***P < 0.001; **P < 0.01; *P < 0.05; *n* = 5–8 animals per group. (*E*) Cumulative dose–response curve of isolated mesenteric arteries in response to either the vasodilator ACh or the vasoconstrictor phenylephrine (Phe). Data are means ± SEM, *n* = 7 mesenteric rings; two-way ANOVA; *P* < 0.05; *, vs. C57BL/6; #, vs. MyD88^−/−^; §, vs. MyD88^−/−^+AngII.

AngII infusion (1 mg/kg/d for 7 days) induced endothelial dysfunction (impaired response to the endothelium dependent vasodilator, acetylcholine) and increased vascular reactive oxygen species (ROS) formation in C57BL/6 mice. MyD88 deficiency significantly attenuated endothelial dysfunction (*Figure 1A*). MyD88 is the major adapter protein in toll-like receptor (TLR) signalling. AngII and ROS might induce the formation of endogenous TLR ligands also under sterile conditions.^27^ Especially TLR4 has been implicated in hypertension and cardiovascular disease (CVD), but also other TLRs like TLR2,^28^ TLR7,^29^ and TLR9^30,31^ have been discussed to be of relevance in this context. Importantly, vascular relaxation studies revealed no significant differences between C57BL/6 and TLR2^−/−^, TLR4^−/−^, TLR7^−/−^, and TLR9^−/−^ in response to AngII (see Supplementary material online, *Figure S1*). This indicates that MyD88 *per se* is critical for vascular inflammation, endothelial dysfunction, and arterial remodelling induced by AngII, independently of one single TLR-signalling pathway.

Furthermore, MyD88^−/−^ mice were protected from AngII-induced NADPH oxidase (Nox)2 expression on the mRNA and protein level, a major source of superoxide within the vessel wall. mRNA expression of heme oxygenase-1 (*hmox1*), an oxidative stress response gene, was decreased in AngII-infused MyD88^−/−^ mice compared to C57BL/6 controls (*Figure 1B*; Supplementary material online, *Figure S2*). Consequently, AngII-induced vascular superoxide formation was increased in C57BL/6 but not in MyD88^−/−^ mice (*Figure 1C*). Nitric oxide bioavailability as assessed by electron paramagnetic resonance spectroscopy was preserved in the vasculature of AngII-infused MyD88^−/−^ mice (*Figure 1D*), implying protection from superoxide-mediated scavenging of NO. Interestingly, vascular endothelial function was preserved in the microvasculature as well, indicating that resistance vessels as an important contributor to blood pressure regulation were also protected from AngII-induced endothelial dysfunction in MyD88^−/−^ mice (*Figure 1E*). This data implies that MyD88 is a critical determinant of superoxide-induced endothelial dysfunction.

**Figure 2:**
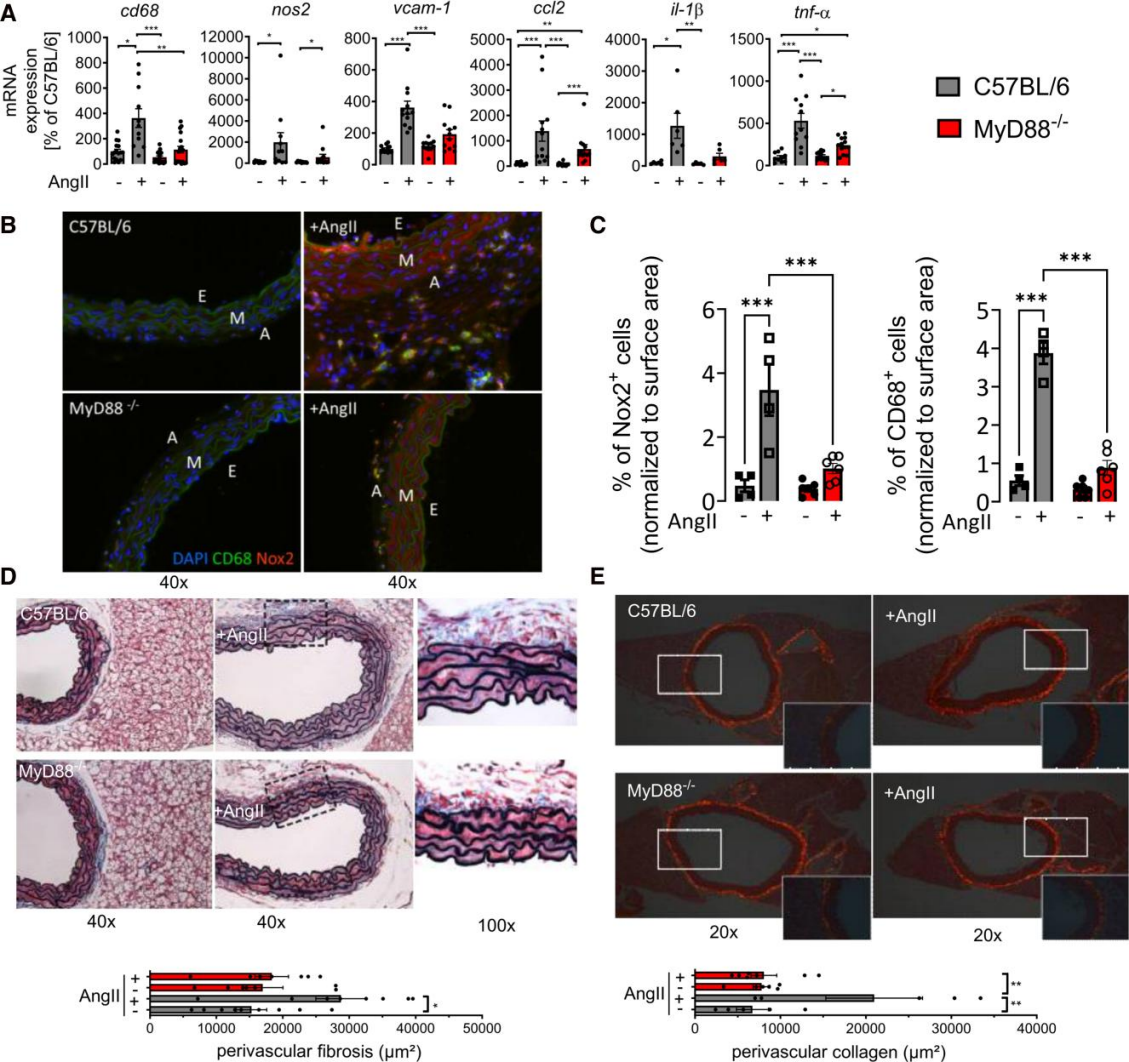
Angiotensin II-induced vascular inflammation depends on MyD88. Inflammation of the aortic wall was investigated by different methods. (*A*) Aortic mRNA expression levels of *cd68* (*n* = 16–18), inducible NO synthase (*nos2*, *n* = 10–12), vascular cell adhesion protein 1 (*vcam-1*, *n* = 12), monocyte chemoattractant protein-1 (*ccl2*, *n* = 12) and interleukin 1 beta (*il-1β*, *n* = 5–6), and tumour necrosis factor alpha (*tnf-α*, *n* = 11–13) were assessed by qRT-PCR. Data are mean ± SEM displayed as percentage of C57BL/6 controls; Kruskal–Wallis test; ***P < 0.001; **P < 0.01; *P < 0.05. (*B* and *C*) Infiltration of phagocytic cells into the aortic wall was detected by immunofluorescence staining of paraffin-embedded aortic sections using specific antibodies for the phagocytic NADPH oxidase (Nox2) or CD68 as a macrophage cells marker. 40-fold magnification; 40-fold magnification in addition for the AngII groups. Representative images (*B*) and quantification of Nox2^+^ or CD68^+^ cells normalized to surface area of aorta section (*C*). Green, CD68; red, Nox2; blue, DAPI; E, endothelium; M, media; A, adventitia. Data are mean ± SEM, *n* = 4–6 animals, one-way ANOVA, and Tukey’s multiple comparison test; ***P < 0.001. (*D* and *E*) Histochemical stainings of aortic paraffin sections. (*D*) Verhoeff’s elastic (VES)–Masson’s trichrome (MTC) stain was used to simultaneously visualize elastic fibres (black), muscular tissue (red), and extracellular matrix (blue). (*E*) Sirius red staining was employed to visualize interstitial collagen fibres. Sections were photographed under polarized light. For both, results are expressed as µm² positive area per optical field at 200× magnification. Data are mean ± SEM, *n* = 5–6 animals, Kruskal–Wallis test; ***P < 0.001; **P < 0.01; *P < 0.05.

Vascular inflammation driven by myelomonocytic cells is pivotal for the development of arterial hypertension.^6,32^ To examine if the improved vascular phenotype in MyD88^−/−^ mice is causally connected to reduced inflammation of the vessel wall, we measured mRNA expression levels of specific pro-inflammatory markers known to be increased in response to AngII (*Figure 2A*). *In vivo* treatment with AngII significantly increased aortic mRNA expression of the macrophage marker cluster of differentiation 68 (*cd68*), vascular cellular adhesion molecule 1 (*vcam-1*), important for leukocyte adhesion and the pro-inflammatory cytokine Interleukin-1β (*il-1β*) in wild type but not in MyD88^−/−^ mice. mRNA expression of inducible NO synthase (*nos2*) and *ccl2*, encoding for monocyte chemoattractant protein 1 (MCP-1), the most relevant chemokine for monocyte attraction in hypertension^33^ and tumour necrosis factor alpha (*tnf-α*) were upregulated in in response to AngII infusion in both wild type and MyD88^−/−^ mice, but to a lesser extent in the MyD88 knockouts.

Immunofluorescence staining analysis revealed an accumulation of CD68^+^ and Nox2^+^ inflammatory cells in aortas of AngII-treated control mice, which was blunted in MyD88^−/−^ mice (*Figure 2B* and *C*). Likewise, AngII induced vascular remodelling as assessed by perivascular fibrosis and collagen deposition in the aortas of C57BL/6, but not of MyD88^−/−^ mice (*Figure 2D* and *E*). Collectively, our results demonstrate that vascular inflammation strongly depends on intact MyD88-mediated signalling.

**Figure 3:**
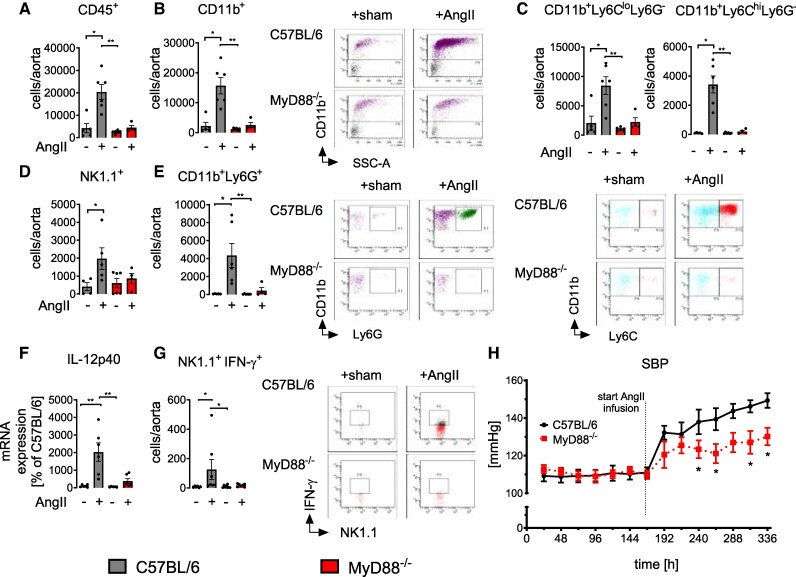
MyD88 deficiency attenuates ATII-induced myeloid and NK cell aortic influx and blood pressure increase. (*A–E*) Flow cytometric quantification (cells/aorta) of overall CD45^+^ leukocytes (*A*), CD11b^+^ myeloid cells (*B*), CD11b^+^Ly6G^−^ Ly6C^hi^ and ^lo^ monocytes (*C*), NK1.1^+^ NK cells (*D*), and CD11b^+^Ly6G^+^ neutrophils (*E*). Kruskal–Wallis test; *n* = 5–6 animals per group; C57BL/6 vs. C57BL/6 +AngII selective pair testing for NK1.1 and CD11b^+^Ly6G^+^ cells. Representative dot plots are shown for selective groups. (*F*) Aortic mRNA expression levels of Interleukin-12 (IL-12p40, *n* = 5–6) assessed by qRT-PCR. Data are displayed as percentage of C57BL/6 controls; Kruskal–Wallis test. (*G*) Analysis of the influx of interferon-γ (IFN-γ)-competent NK cells into the aortic wall in sham or AngII-treated MyD88^−/−^ in comparison to C57BL/6 controls. Left, quantification and right, representative dot plots. *n* = 6–8, Kruskal–Wallis test. (*H*) Telemetrically recorded systolic blood pressure (SBP) of C57BL/6 (*n* = 5) and MyD88^−/−^ (*n* = 5) mice. Summary of 336 h measurement. AngII infusion started after 168 h. Two-way ANOVA; *P* < 0.05; *, vs. C57BL/6. (*A–H*) Data are mean ± SEM; ***P < 0.001; **P < 0.01; *P < 0.05.

MyD88 deficiency dampens reciprocal innate immune cell activation of NK cells and myelomonocytic cells. To study the nature of inflammatory cells infiltrating into the aortas more specifically, we performed flow cytometric analysis of vascular tissue. AngII-infused C57BL/6 mice revealed a significant accumulation of CD45^+^ leukocytes (more specifically CD11b^+^ myelomonocytic cells; *Figure 3A* and *B*) and of CD11b^+^Ly6G^−^ monocytes (both Ly6C^lo^ and Ly6C^hi^; *Figure 3C*) as well as NK1.1^+^ natural killer (NK) cells (*Figure 3D*) and CD11b^+^Ly6G^+^ neutrophils (*Figure 3E*) in the aorta, which was significantly lower in MyD88^−/−^ mice. Based on our previous findings that AngII-induced vascular inflammation depends on mutual activation of NK cells and monocytes,^34^ we measured levels of the two primarily involved cytokines IL-12 and interferon (IFN)-γ. AngII infusion significantly increased *il-12p40* mRNA expression (*Figure 3F*) and drastically increased the number of IFN-γ competent NK1.1^+^ NK cells in aortas of C57BL/6 mice, but not in MyD88^−/−^ mice (*Figure 3G*), suggesting that the pro-inflammatory circuit of NK cells and monocytes in hypertension requires MyD88.

In addition to attenuated inflammatory cell infiltration, endothelial dysfunction, and vascular remodelling, AngII-induced systolic blood pressure increase was significantly reduced in MyD88^−/−^ mice compared to C57BL/6 mice (*Figure 3H*; Supplementary material online, *Table S5*). These findings indicate that MyD88 is required for the AngII-induced cellular immune reaction, the amplification of myeloid cell inflammatory responses, and the ensuing development of arterial hypertension in mice.

### MyD88 expressed by haematopoietic cells drives AngII-induced vascular inflammation and endothelial dysfunction

**Figure 4:**
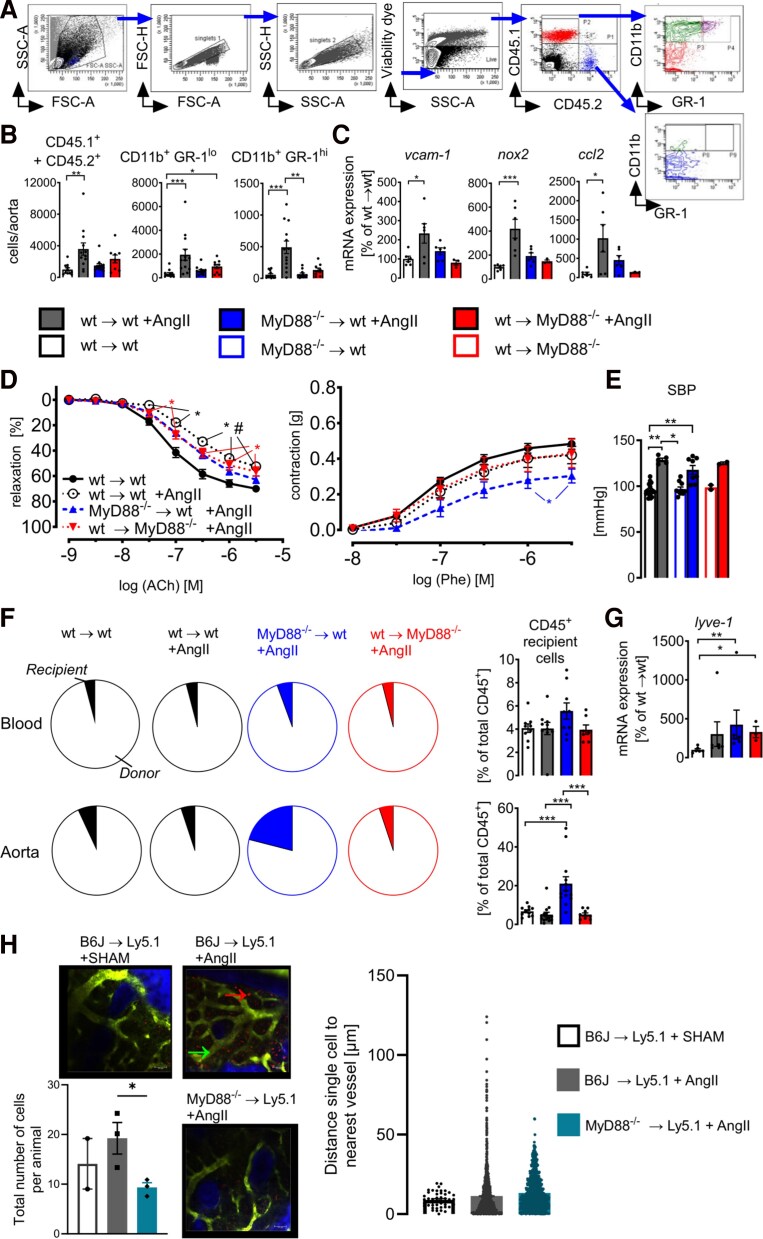
MyD88-dependent vascular inflammation is driven by infiltrating immune cells originating from the bone marrow. Wild-type (wt) or MyD88^−/−^ mice were lethally irradiated and reconstituted with bone marrow from wt or MyD88^−/−^ mice. (*A* and *B*) Flow cytometric analysis of the influx of CD45.1^+^ and CD45.2^+^ leukocyte subpopulations into the aortic wall of chimeric mice. (*A*) Gating strategy. Exemplary the gating of an aortic sample of an AngII-infused CD45.2^+^ C57BL/6 wild-type mouse which was lethally irradiated and reconstituted with CD45.1 bone marrow of a Ly5.1 mouse is shown. (*B*) Quantification (living cells/aorta) of overall CD45+ leukocytes (CD45.1^+^ and CD45.2^+^), CD11b^+^GR-1^lo^ monocytes, and CD11b^+^GR-1^hi^ cells. (*C*) Aortic mRNA expression levels of vascular cell adhesion protein-1 (*vcam-*1, *n* = 3–6), phagocytic NADPH oxidase (*nox2*, *n* = 3–6), and monocyte chemoattractant potein-1 (*ccl-2*, *n* = 3–6) were assessed by qRT-PCR. Data are displayed as percentage of wt→wt controls; Kruskal–Wallis test. (*D*) Assessment of the vascular function of sham or AngII-treated chimeric mice by isometric tension studies of isolated aortic segments. Cumulative concentration relaxation curves in response to acetylcholine (ACh) or constriction curves in response to phenylephrine (Phe). Data are mean ± SEM of *n* = 13–23 (ACh) and *n* = 8–13 (Phe) per group. Two-way ANOVA; *, *P* < 0. 05 vs. wt→wt; #, *P* < 0.05 vs. MyD88^−/−^→wt + AngII; ). (*E*) Tail-cuff measurement of systolic blood pressure (SBP). Data of *n* = 5 (wt→wt + AngII), *n* = 18 (wt→wt) *n* = 8 (MyD88^−/−^→wt ± AngII), *n* = 2 (wt→MyD88^−/−^ ±AngII) animals. Kruskal–Wallis test. (*F*) Comparison of chimerism in blood and aorta. Ratio of donor or recipient derived CD45^+^ cells calculated as percentage of total CD45^+^ cells. Data of *n* = 9–12 animals per group; Kruskal–Wallis test. (*G*) mRNA expression of arterial macrophage marker lymphatic vessel endothelial hyaluronic acid receptor-1 (*lyve-1*), *n* = 3–6 animals per group, Kruskal–Wallis test. (*H*) *In vivo* microscopy of the calvarium 48 h after bone marrow (CMTMR-stained) transplantation and AngII infusion. The bone (blue), migrated cells (red), and endothelium (green) were visualized. The number of migrated cells from vessels to calvarium and distance to endothelium in the acquired images were then analysed. (*B*, *C*, and *E–G*) Data are mean ± SEM; ***P < 0.001; **P < 0.01; *P < 0.05.

MyD88 is ubiquitously expressed in both immune and nonimmune cells. To investigate whether the protection from AngII-induced vascular injury conveyed by global MyD88 deficiency is related to the function of MyD88 within inflammatory cells, we generated bone marrow (BM)-chimeric mice. AngII infusion significantly increased vascular accumulation of CD11b^+^GR-1^hi^ and CD11b^+^GR-1^lo^ myelomonocytic cells in irradiated C57BL/6 mice, control-reconstituted with BM cells isolated from C57BL/6 mice (wt→wt vs. wt→wt +AngII, *Figure 4A* and *B*). AngII-infused wt→wt mice revealed vascular dysfunction, increased systolic blood pressure, and mRNA expression of leukocyte infiltration markers *vcam-1*, *nox2*, and *ccl2* (*Figure 4C–E*). In contrast, AngII-infused chimeric mice, transplanted with BM cells of MyD88^−/−^ mice (MyD88^−/−^→wt +AngII), had reduced vascular infiltration of CD11b^+^Gr-1^hi^ myelomonocytic cells, a partial protection from vascular dysfunction compared to wt→wt +AngII mice. While systolic blood pressure was not significantly increased compared to MyD88^−/−^→wt mice. AngII infusion of irradiated wt mice restored with the BM of MyD88^−/−^ (MyD88^−/−^→wt) did not increase systolic blood pressure compared to sham-infused MyD88^−/−^→wt mice. wt→MyD88^−/−^ mice +AngII showed an intermediate phenotype between MyD88^−/−^→wt +AngII and wt→wt +AngII mice (*Figure 4B* and *E*).

In circulating blood, donor chimerism level was stable around 95 ± 2% among all groups. The chimerism of wt→wt ± AngII and wt→MyD88^−/−^ +AngII mice in the aortas was comparable to blood, meaning that >90% of accumulated aortic CD45^+^ immune cells originated from the donor and were of BM origin. Interestingly, in aortas of mice with MyD88 deficiency in the haematopoietic compartment (MyD88^−/−^→wt), we found relative upregulation of CD45^+^ immune cells with non-donor origin (*Figure 4F*; percentage of cells with recipient origin in aorta: 5 ± 1% wt→wt +AngII; 21 ± 4% MyD88^−/−^→wt +AngII; 5 ± 1% wt→MyD88^−/−^ +AngII, respectively).

To study whether this phenomenon was caused by expansion of a tissue-resident lineage of BM-independent myeloid cells or by preferred infiltration of remnant BM cells of the irradiated host, we performed mRNA expression analysis of genes marking arterial macrophages.^35^ mRNA expression of the arterial macrophage marker lymphatic vessel endothelial hyaluronic acid receptor 1 (*lyve-1*) was increased in AngII-infused mice. MyD88^−/−^ → wt +AngII mice showed the highest and significant upregulation, compatible with a compensatory reaction of resident MyD88-competent macrophages in the recipient (*Figure 4G*).

In order to better understand the mechanism of MyD88-dependent mobilization of immune cells from the BM, we investigated wt control mice (C57BL/6J) that had been adoptively transferred with fluorescent dye-labelled BM-derived cells from either wt or MyD88^−/−^. The egress of inflammatory cells from the BM as well as the migratory capacity, assessed by intravital microscopy of the calvarium, was increased by AngII infusion *in vivo* and depended on the presence of MyD88 (*Figure 4H*).

Together, these results indicate that MyD88 expressed in BM-derived inflammatory cells plays an important role in determining the extent of vascular inflammation. MyD88-dependent signalling in non-haematopoietic/tissue-resident cells contributes in part to AngII-induced vascular injury.

### MyD88 within myelomonocytic cells drives AngII-induced monocyte proliferation and vascular inflammation

**Figure 5:**
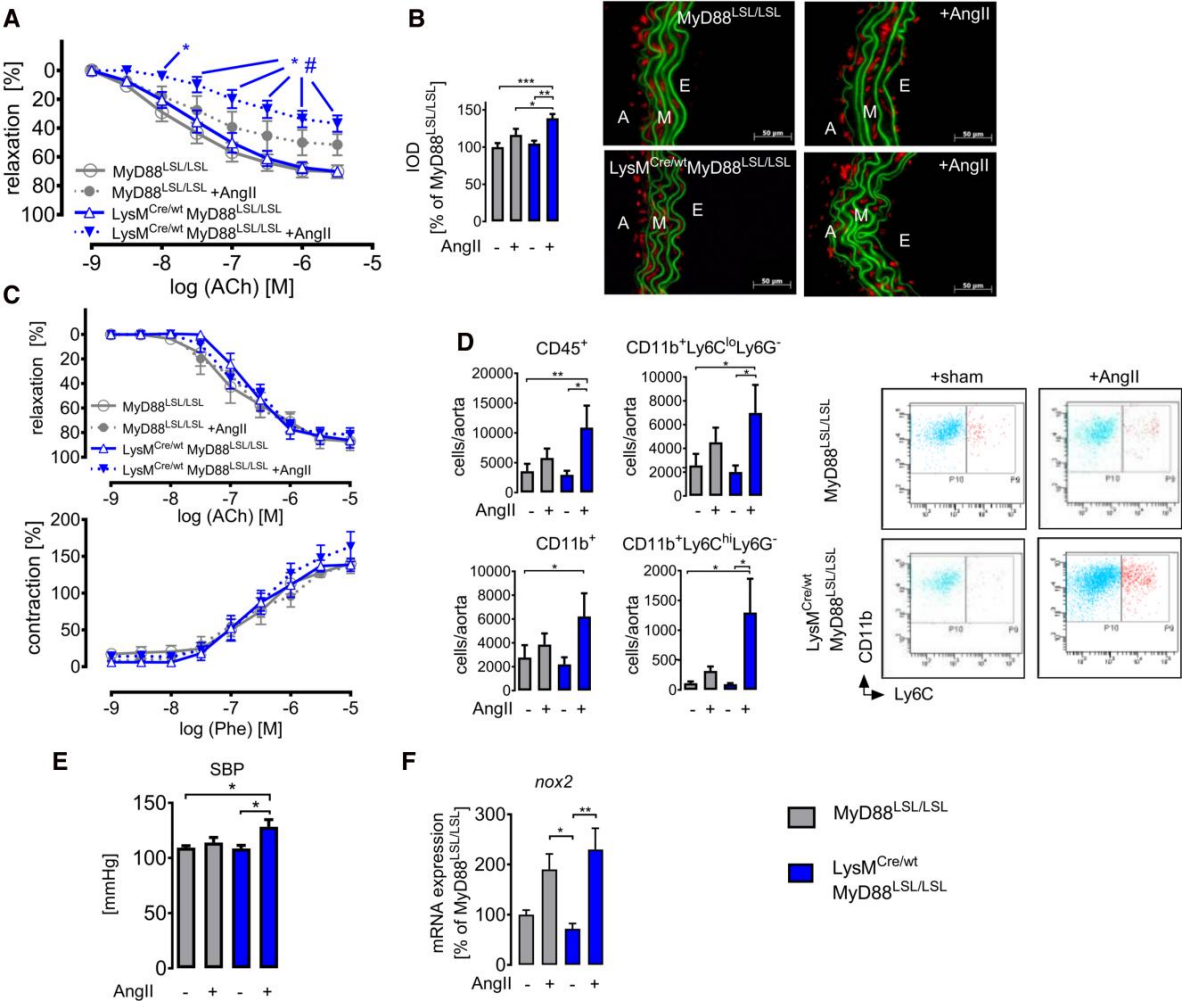
MyD88 expression in LysM^+^ cells drives endothelial dysfunction, vascular inflammation, and arterial hypertension in AngII-infused mice. (*A*) Cumulative concentration–relaxation curves of isolated aortic rings from sham-treated and AngII-treated MyD88^LSL/LSL^ and LysM^Cre/wt^ MyD88^LSL/LSL^ mice in response to endothelium dependent vasodilator acetylcholine (ACh). Data are mean ± SEM of *n* = 6–10 animals per group; two-way ANOVA; *P* < 0.05; *, vs. MyD88^LSL/LSL^; #, vs. LysM^Cre/wt^MyD88^LSL/LSL^. (*B*) Oxidative fluorescence microtopography. Right: Representative photomicrographs of isolated aortic segments incubated with DHE (1 mM, 30 min at 37°C). Green, laminae (autofluorescence); red fluorescence, superoxide formation; E, endothelium; M, media; A, adventitia. Left: Densitometric analysis, integrated optical density (IOD); *n* = 3 independent experiments, one-way ANOVA and Bonferroni’s multiple comparison test. (*C*) Cumulative dose–response curve of isolated mesenteric arteries in response to either the vasodilator ACh or the vasoconstrictor phenylephrine (Phe). Data are means ± SEM, *n* = 6–8 mesenteric rings; two-way ANOVA. (*D*) Flow cytometric analysis of influx of CD45^+^ leukocytes and leukocyte subpopulations into the aortic wall. Left: Quantification (living cells/aorta) of overall CD45^+^ leukocytes, CD11b^+^ myeloid cells, and CD11b^+^Ly6C^hi^Ly6G^−^ and CD11b^+^Ly6C^lo^Ly6G^−^ cells. *n* = 7–11 animals per group, Kruskal–Wallis test, and selected pairs of columns (LysM^Cre/wt^MyD88^LSL/LSL^ vs. LysM^Cre/wt^MyD88^LSL/LSL^ +AngII) were tested in addition. Right: Selected representative dot plots. (*E*) Tail-cuff measurement of systolic blood pressure (SBP). *n* = 10 animals per group, one-way ANOVA. (*F*) Aortic mRNA expression levels of phagocytic NADPH oxidase (*nox2*) were assessed by qRT-PCR. Data are displayed as percentage of MyD88^LSL/LSL^ controls; *n* = 5–6 animals per group, Kruskal–Wallis test. (*B* and *D–F*) Data are mean ± SEM; ***P < 0.001; **P < 0.01; *P < 0.05.

Pro-inflammatory/classically activated monocytes are characterized as Ly6C^hi^CCR2^+^CX_3_CR1^lo^L-selectin^+^.^36–38^ AngII-infused C57BL/6 mice showed a significantly increased expression of CD62L (L-selectin) in PBMCs as well as aortic lysates, which was blunted in MyD88^−/−^ mice (see Supplementary material online, *Figure S3*). To pinpoint the role of MyD88 expressed by myelomonocytic cells *in vivo*, we investigated mice with a LysM^+^ cell–specific rescue of MyD88. AngII-infused MyD88^LSL/LSL^ mice harbouring the silenced stop-codon flanked MyD88 gene did not show significant vascular dysfunction (in both aorta and mesenteric arteries) and had attenuated vascular superoxide formation (*Figure 5A–C*), vascular inflammation, and blood pressure increase (*Figure 5D* and *E*; Supplementary material online, *Figure S4*), comparable to the MyD88^−/−^ strain.

In contrast, LysM^Cre/wt^MyD88^LSL/LSL^ mice selectively rescuing MyD88 expression exclusively in myelomonocytic cells developed markedly impaired vascular function (more pronounced in the aorta than in mesenteric arteries) and vascular superoxide formation in response to AngII, as well as higher *nox2* mRNA levels (*Figure 5A*–*C* and *F*). Furthermore, flow cytometric analysis revealed a significantly increased infiltration with CD45^+^ leukocytes, especially CD11b^+^Ly6G^−^ Ly6C^hi^ and Ly6C^lo^ myeloid cells (*Figure 5D*), comparable to C57BL/6 mice. Consistent with the increased vascular inflammation and dysfunction, systolic blood pressure increased significantly in AngII-infused LysM^Cre/wt^MyD88^LSL/LSL^ mice (*Figure 5E*). These results clearly demonstrate that LysM^+^ myeloid cells are required and sufficient to convey MyD88-dependent vascular inflammation and blood pressure increase in response to AngII.

### MyD88 protein significantly interacts with hypertension-associated gene products

**Figure 6:**
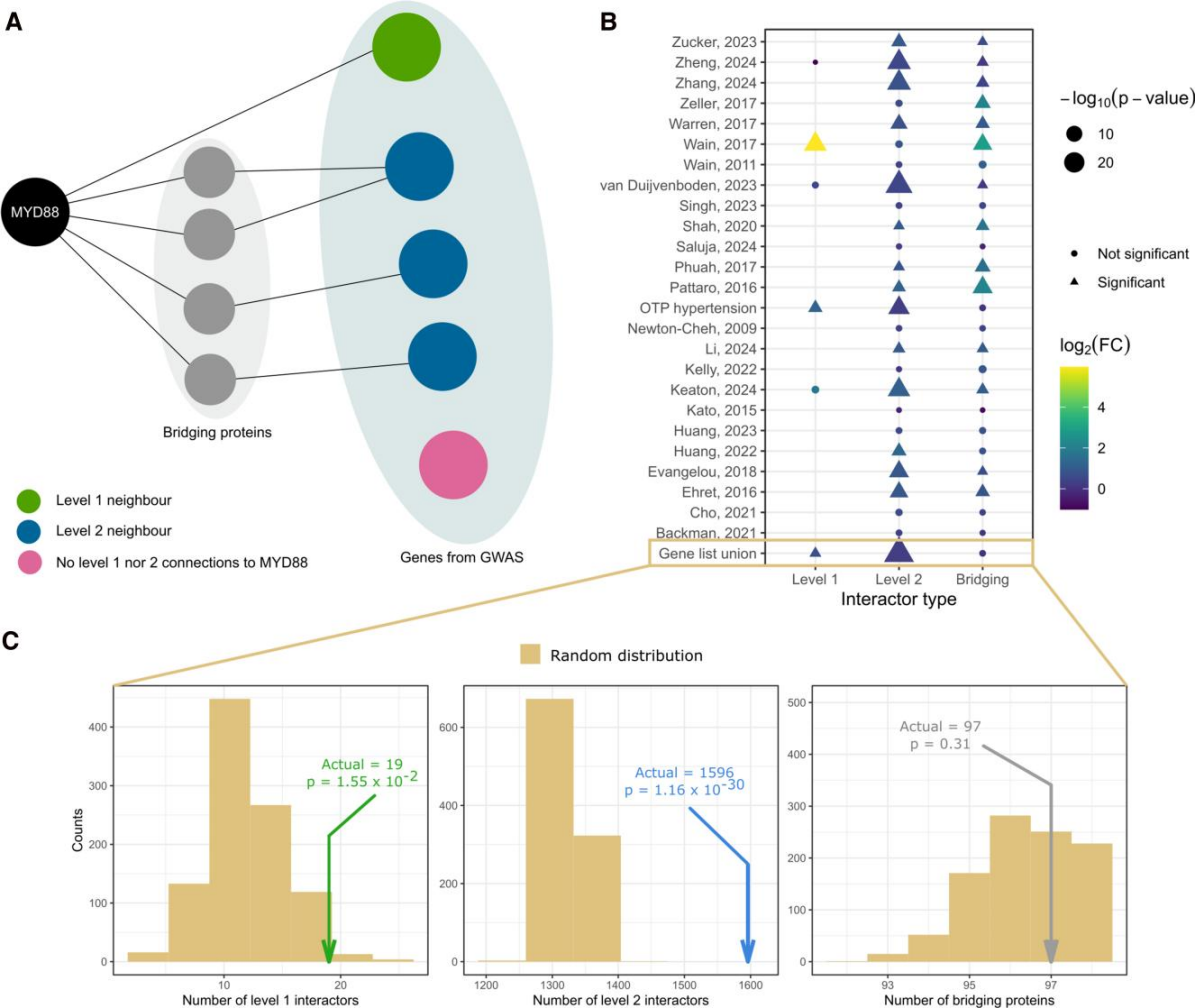
MYD88 interacts with hypertension-associated genes via a significant number of intermediaries. (*A*) We investigated the connections in the human protein interaction network between MyD88 and proteins encoded by hypertension-associated genes according to GWASs or meta-analyses. We compared the number of direct interactors (level-1 neighbours, green), level-2 interactors (blue), and the proteins bridging the latter with MyD88 (grey) with a reference null model to compute fold changes and *P*-values. (*B*) The list of GWASs or meta-analyses^39–64^ reporting hypertension-associated loci with the summary statistics described in (*A*) for level-1, level-2, and bridging interactors of MYD88. The union of the genes reported by the studies highlighted in boldface was used to compute the statistics presented in (*C*). (*C*) Detailed comparison between the number of level-1, level-2, and bridging interactors of MYD88 and the respective reference null models constructed with 1000 randomly sampled sets of gene products of the same size as in the union of the selected studies (boldface in panel (*B*)) and with the same degree distribution. The colour-code is the same as in (*A*).

To corroborate our findings, we explored published datasets from a multitude of GWASs and meta-analyses thereof to study whether MyD88 is of putative significance with regard to genetic risk of arterial hypertension traits. Using the HIPPIE database, we investigated the connections in the human protein interaction network between MyD88 and proteins encoded by the sets of genes from 25 GWAS-based studies reporting hypertension-associated loci and compared these numbers with a reference null model (see Material and methods for details, *Figure 6*, *Table 1*, and Supplementary material online, *Table S1*). Sixteen of the 25 studies provided significant interactions of MyD88 with these proteins, with Wain *et al.* (2017) being particularly significant (*Figure 6B*). This latter GWAS set includes a gene encoding one level-1 (direct) interactor of MyD88, the NFKBIA gene (encoding for nuclear factor kappa B inhibitor alpha), and three genes encoding level-2 interactors of MyD88, the AKT2, SEPTIN9, and EBF2 genes (encoding for the serine/threonine kinase Akt2, septin9, and EBF transcription factor 2, respectively). The latter were interconnected via 20 bridging proteins, all of which are included in pathophysiologically relevant regulatory pathways of inflammation like phosphatidylinositol 3-kinase signalling (AKT1), nuclear factor kappa B signalling (IKBKG, IKBKB, IKBKE, TBK1), interleukin-1b signalling (IRAK1, IRAK4), or tumour necrosis factor signalling (NGFR, TRAF2, TRAF6), in stress response pathways of proliferation and differentiation (MAPK14, MAP3K7, MAP3K1, SYK), in activation states of myeloid cells (CD14) or to danger associated molecular patterns (TLR2). Importantly, the set of bridging proteins was significantly larger than those obtained from a reference null model network (see Material and methods for details). This was not the case for the number of level-2 interactors (see Supplementary material online, *Table S1*).

**Table 1:** Level-1 MyD88 interactors from genome-wide association study

Myd88 interactor	Gene description	UniProtAcc	Study
TRAF3	TRAF3 interacting protein 3	B1AJU2	^41^
NFKBIA	NFKB inhibitor alpha	P25963	^41^
IKBKB	Inhibitor of nuclear factor kappa B kinase subunit beta	O14920	^41^
TLR9	Toll-like receptor 9	Q9NR96	^41^
TLR7	Toll-like receptor 7	B2R9N9	^41^
MAP3K1	Mitogen-activated protein kinase kinase kinase 15	Q6ZN16	^42^
RAC1	PRAC1 small nuclear protein	Q96KF2	^43^
UBAP1	Ubiquitin associated protein 1	Q9NZ09	^44^
SARM1	Sterile alpha and TIR motif containing 1	Q05B42	^41^
BANK1	B cell scaffold protein with ankyrin repeats 1	Q8NDB2	^44^
SMAD6	SMAD family member 6	O43541	^41^
IL1B	Interleukin 1 beta	P01584	^41^
TLR2	Toll-like receptor 2	A0A0S2Z4S4	^41^
TLR10	Toll-like receptor 10	A0A024R9W4	^41^
IL1R1	Interleukin 1 receptor type 1	P14778	^41^
TXN	Thioredoxin	P10599	^41^
FADD	Fas associated via death domain	Q13158	^41^
IRF5	Interferon regulatory factor 5	Q13568	^44^
SMAD3	SMAD family member 3	P84022	^44^

Nineteen level-1 Myd88 interactors from the union of all studies with significant results.

We also took the union of the hypertension-associated genes from the 16 studies where MyD88 has a significant number of connections with at least one interactor type (i.e. level-1, level-2, or bridging interactors; *Figure 6B*). With this set of genes, we repeated the above analysis and found that MyD88 is connected to 19 level-1 and 1596 level-2 hypertension-associated gene products via 97 bridging nodes with these numbers being greater than expected by chance (*Figure 6C*; Supplementary material online, *Table S1*). The direct interactors are listed in *Table 1* and are all linked to Toll-like receptor signalling and inflammatory responses (adjusted *P* < 1 × 10^−8^, enrichment analysis using the biological process aspect of the Gene Ontology).

In all, this analysis indicates that MyD88 is linked to established genes that are associated with various hypertension traits that are all related to inflammation or immune activation. This result underlines the importance of studying network connectivity patterns to interpret the significance of GWAS datasets beyond direct gene–trait associations.

### High levels of MyD88 expression in circulating leukocytes are associated with increased all-cause mortality and chronic heart failure in individuals with arterial hypertension

Next, we investigated whether our findings would have implications for humans with arterial hypertension. We assessed mRNA expression in monocytes as well as PBMCs of 1274 (and 419, respectively) participants of the Gutenberg Health Study (see *Table 2* for baseline characteristics and Supplementary material online, *Table S4* for grouping by the risk of high (>10.7) and low (≤10.7) expression of MyD88) and found that expression patterns followed a Gaussian distribution (see Supplementary material online, *Figure S5A* and *B*). In a multivariate linear regression analysis adjusted for age, sex, storage time, RIN number, and plate layout, monocytic MyD88 mRNA levels were significantly associated with CD14 mRNA in monocytes, marking classically activated monocytes (*Table 3*).

**Table 2:** Cohort characteristics of the participating individuals from Gutenberg Health Study

	All (5000)	Men (2540)	Women (2460)
Sex (women)	49.2% (2460)	0% (0)	100.0% (2460)
Age (y)	55.5 ± 10.9	56.0 ± 10.9	55.0 ± 11.0
CVRFs			
Diabetes (yes)	9.9% (493)	12.1% (306)	7.7% (187)
Obesity (yes)	24.1% (1204)	25.4% (645)	22.7% (559)
Smoking (yes)	19.2% (959)	20.8% (527)	17.6% (432)
Hypertension (yes)	51.3% (2564)	56.1% (1426)	46.3% (1138)
Incident hypertension (yes)	21.9% (465)	25.3% (249)	18.9% (216)
Dyslipidaemia (yes)	45.0% (2245)	54.9% (1391)	34.8% (854)
FH of MI/stroke (yes)	23.3% (1166)	22.1% (561)	24.6% (605)
BMI (kg/m^2^)	27.2 ± 4.8	27.7 ± 4.1	26.7 ± 5.4
Height (cm)	170 ± 9	177 ± 7	164 ± 7
WHtR	0.551 ± 0.079	0.562 ± 0.071	0.538 ± 0.085
SBP (mmHg)	133 ± 18	136 ± 17	130 ± 18
DBP (mmHg)	83.2 ± 9.5	84.5 ± 9.6	81.8 ± 9.2
HR (b.p.m.)	68.9 ± 10.9	67.8 ± 11.1	69.9 ± 10.5
Cholesterol (mmol/L)	223 ± 41	218 ± 41	229 ± 41
HDL (mg/dL)	56.5 ± 15.8	49.7 ± 13.0	63.4 ± 15.4
LDL (mg/dL)	142 ± 36	141 ± 35	143 ± 36
LDL/HDL	2.68 ± 0.95	2.97 ± 0.96	2.39 ± 0.84
Triglycerides (mg/dL)	107.0 (79.4/149.4)	118.0 (86.4/165.0)	99.0 (74.0/134.0)
HbA1c (%)	5.40 (5.10/5.80)	5.40 (5.10/5.80)	5.30 (5.00/5.70)
Diseases			
MI (yes)	3.1% (156)	4.7% (119)	1.5% (37)
Stroke (yes)	1.9% (95)	2.3% (59)	1.5% (36)
AF (yes)	2.7% (136)	3.9% (98)	1.6% (38)
PAD (yes)	4.1% (203)	4.3% (109)	3.8% (94)
CAD (yes)	4.6% (226)	6.9% (173)	2.2% (53)
CHF (yes)	1.5% (77)	1.6% (40)	1.5% (37)
Heart failure (HF) (yes)	5.4% (259)	5.1% (125)	5.6% (134)
Diastolic dysfunction (DDo) (yes)	23.2% (1027)	23.5% (522)	22.8% (505)
DVT (yes)	4.0% (198)	2.9% (73)	5.1% (125)
PE (yes)	0.2% (11)	0.2% (5)	0.2% (6)
COPD (yes)	4.8% (239)	4.6% (116)	5.0% (123)
CKD (yes)	4.5% (225)	4.3% (108)	4.8% (117)
CLD (yes)	0.7% (37)	0.5% (13)	1.0% (24)
Cancer (yes)	9.0% (448)	8.0% (202)	10.0% (246)
Acute infection (yes)	18.3% (908)	17.5% (441)	19.2% (467)
Endothelial and vascular function			
Baseline RI	70.0 (57.0/78.0)	75.0 (66.0/82.0)	62.0 (52.0/73.0)
Hyperaemic RI	68.0 (53.0/78.0)	75.0 (66.0/81.0)	58.0 (46.0/70.0)
RI difference	2.00 (−4.00/9.00)	1.00 (−3.08/5.00)	4.00 (−4.00/13.00)
fRHI	0.607 (0.282/0.888)	0.445 (0.172/0.752)	0.757 (0.462/1.007)
Mean BL pre FMD (mm)	4.34 (3.69/4.97)	4.93 (4.53/5.31)	3.72 (3.30/4.13)
FMD (%)	7.15 (4.43/10.72)	6.00 (3.77/8.63)	8.98 (5.59/13.07)
SI (m/s)	8.76 (6.62/11.20)	10.11 (7.48/12.28)	7.61 (6.09/9.65)
ABI	0.976 ± 0.127	0.980 ± 0.143	0.971 ± 0.108
IMT (mm)	0.652 ± 0.129	0.670 ± 0.138	0.633 ± 0.117
Genetic			
MYD88	10.22 ± 0.33	10.22 ± 0.33	10.21 ± 0.33
CD14	14.5 ± 0.1	14.5 ± 0.2	14.6 ± 0.1
Storage time	314 ± 92	311 ± 91	318 ± 92
RIN	9.36 ± 0.43	9.38 ± 0.41	9.33 ± 0.46
rs7744			
A/A	70.9% (2959)	70.4% (1501)	71.4% (1458)
A/G	26.6% (1109)	26.7% (569)	26.4% (540)
G/G	2.6% (107)	2.9% (62)	2.2% (45)
rs4988453			
C/C	90.2% (3765)	90.5% (1929)	89.9% (1836)
C/A	9.6% (402)	9.3% (199)	9.9% (203)
A/A	0.2% (8)	0.2% (4)	0.2% (4)

CVRFs, cardiovascular risk factors; FH of MI/stroke, familial history of myocardial infarction/stroke; BMI, body mass index; WHtR, waist height ratio; SBP, systolic blood pressure; DBP, diastolic blood pressure; HR, heart rate; HDL, high-density lipoprotein; LDL, low-density lipoprotein; HbA1c, haemoglobin A1c; AF, arterial fibrillation; PAD, peripheral artery disease; CAD, carotid artery disease; CHF, chronic heart failure; DVT, deep vein thrombosis; PE, pulmonary embolism; COPD, chronic obstructive pulmonary disease; CKD, chronic kidney disease; CLD, chronic lung disease; RI, resistance index; fRHI, Framingham reactive hyperaemia index; BL, baseline; FMD, flow-mediated dilation; SI, stiffness index (m/s); ABI, ankle brachial index; IMT, intima-media thickness; RIN, RNA integrity number.

**Table 3:** Association of MyD88 expression with CD14 mRNA expression in monocytes

	Estimate	L95% CI	U95% CI	*P*-value
*N*: 1274				
MYD88 (MYD88 > 10.7)	−0.0454	−0.0740	−0.0169	**0**.**0019**
Sex (women)	0.0199	0.00446	0.0353	**0**.**012**
Age (5 y)	0.0016	−0.0019	0.0052	0.36

Linear regression: estimated effects of MyD88 expression on CD14 expression in 1274 individuals of the GHS, adjusted for sex and age as well as storage time, RIN number, and plate layout. *P*-values (<0.05) are highlighted in bold.

Monocytic CD14 and MyD88 mRNA expression, respectively, were not correlated with markers of vascular function, e.g. flow-mediated dilation, vascular stiffness (see Supplementary material online, *Table S2*) or peripheral or carotid artery disease (*Table 4*). Likewise, neither MyD88 SNPs nor monocytic MyD88 mRNA expression was associated with systolic blood pressure (see Supplementary material online, *Table S3*). Interestingly, in the robust Poisson regression models, monocytic MyD88 mRNA was significantly associated with prevalent chronic heart failure in individuals with hypertension (prevalence ratio 8.49, *Table 4*).

**Table 4:** Monocytic MyD88 mRNA expression and prevalence of heart failure

	PR	L95% CI	U95% CI	*P*-value	*N* (events)
Effect of MYD88 (MYD88 > 10.7) on MI	0.52	0.13	2.18	0.37	1270 (26)
Effect of MYD88 (MYD88 > 10.7) on stroke	1.55	0.62	3.87	0.35	1270 (21)
Effect of MYD88 (MYD88 > 10.7) on AF	1.24	0.44	3.48	0.68	1262 (40)
Effect of MYD88 (MYD88 > 10.7) on PAD	1.36	0.64	2.87	0.42	1263 (60)
Effect of MYD88 (MYD88 > 10.7) on CAD	1.00	0.39	2.57	0.99	1259 (58)
Effect of MYD88 (MYD88 > 10.7) on HF (stage C/D)	0.81	0.26	2.49	0.71	1223 (61)
Effect of MYD88 (MYD88 > 10.7) on CHF (self-report)	8.49	2.71	26.58	**0**.**00024**	1273 (17)
Effect of MYD88 (MYD88 > 10.7) on CKD	1.25	0.60	2.61	0.55	1273 (59)
Effect of MYD88 (MYD88 > 10.7) on hypertension	0.99	0.83	1.17	0.86	1274 (628)

Robust Poisson regression models for prevalences. Prevalence ratio (PR) for myocardial infarction (MI), stroke, arterial fibrillation (AF), peripheral artery disease (PAD), carotid artery disease (CAD), heart failure (HF), chronic heart failure (CHF), and chronic kidney disease (CKD) in 1274 individuals of the GHS with available MyD88 mRNA expression data. *P*-values (<0.05) are highlighted in bold. MYD88 is adjusted for sex, age, diabetes, obesity, smoking, dyslipidaemia, FH family history of MI/stroke, storage time, RIN number, and plate layout. Lower limit of the 95% confidence interval (L95% CI) and upper limit of the 95% confidence interval (U95% CI), which is 95% range of values calculated from sample data that is likely to contain the true value of a population parameter.

**Figure 7:**
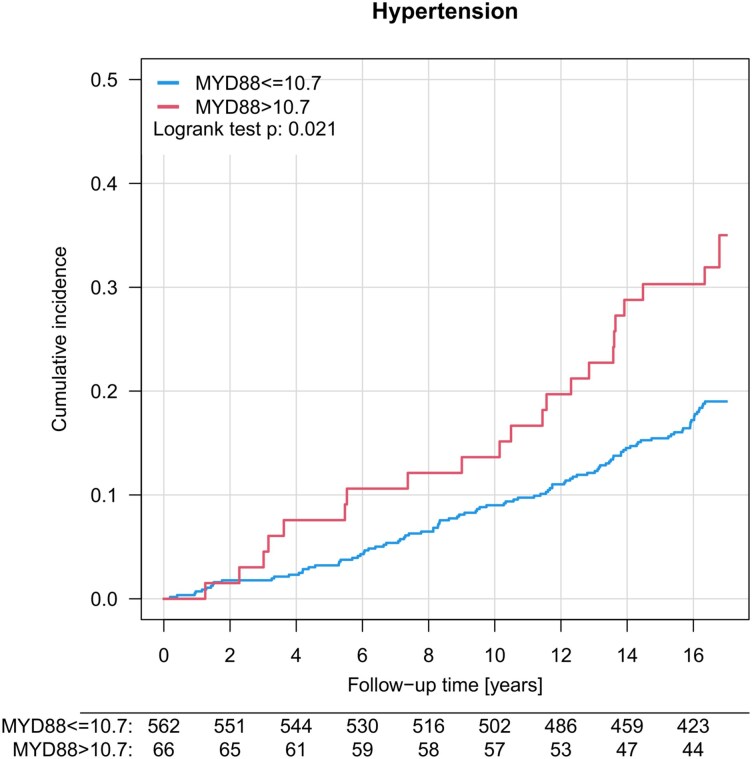
MyD88 mRNA expression in circulating monocytes and all-cause mortality in individuals with arterial hypertension. Kaplan–Meier curves of cumulative all-cause mortality of hypertensive individuals from the Gutenberg Health Study. Individuals were stratified according to MyD88 mRNA expression levels. Median follow-up was 16.5 years; hypertension and MyD88≤10.7 (*n* = 562) with 18.8% deaths (*n* = 103); vs. hypertension and MyD88 > 10.7 (*n* = 66) with 33.33% deaths (*n* = 22). Subjects were tested for differences in cumulative all-cause mortality by log-rank test.

To assess the impact of arterial hypertension on the risk of death, we performed Kaplan–Meier analysis for cumulative incidence of all-cause mortality (median follow-up 16.5 years). Prevalent hypertension significantly increased the risk for all-cause mortality (see Supplementary material online, *Figure S6*). To elucidate a potential impact of MyD88 in this population more specifically, we added the information on MyD88 expression from our biodata bank to our Kaplan–Meier analysis. Hypertensive individuals within the lower level of MyD88 expression (<10.7) had a significantly lower all-cause mortality compared to hypertensive individuals with monocytic levels of MyD88 expression >10.7, (*P* = 0.021; *Figure 7* and *Table 5*) during the 16.5-year follow-up. The value 10.7 corresponds to the 90th percentile (i.e. 10% of participants express MyD88 > 10.7). With this dichotomization, MyD88 expression can best discriminate all-cause mortality in hypertensive patients. These results were recapitulated in principle using PBMCs (*N*: 187 hypertensive individuals, 30 events; Supplementary material online, *Figure S7*). Here, hypertensive individuals with MyD88 mRNA below <9.3 (tertile T1) had significantly less events in the follow-up period of 16.5 years compared to individuals with high MyD88 expression (tertile T3 > 9.6) (*P* = 0.011).

**Table 5:** Effect of MyD88 mRNA expression in monocytes in hypertensive subjects and the risk of death

	HR	L95% CI	U95% CI	*P*-value	C-index
*N*: 628 (125 events)					0.777
MYD88 (MYD88 > 10.7)	1.5245	0.9565	2.4296	0.076	
Sex (women)	0.5250	0.3580	0.7699	0.00097	
Age (5 y)	1.8836	1.6412	2.1617	<0.0001	

Cox regression: estimated effects of high MyD88 expression (MyD88 > 10.7) on all-cause mortality in 1274 individuals with arterial hypertension from the GHS, adjusted for sex and age. Compare *Figure 6*.

Additionally, we included a Cox regression analysis giving information about the interaction of anti-hypersensitive medication in GHS patients with MyD88 > 10.7 to all-cause death (see Supplementary material online, *Table S6*). We could not detect a statistically significant effect of taking antihypertensive drugs (for categorization, see Supplementary material online, *Table S6A*) on the association of MyD88 expression with all-cause mortality.

Next, we explored whether monocytic MyD88 expression might be a useful biomarker to predict incident HF at follow-up. Prevalent arterial hypertension robustly predicted HF at a follow-up of 5 years (*P* = 0.023), and in the subsample of hypertensive patients, MyD88 mRNA levels above 10.7 were not associated with higher rate of incident HF (*P* = 0.45, Supplementary material online, *Figure S8*).

## Discussion

Here, we demonstrate the crucial role of MyD88 expressed by myeloid cells for vascular inflammation, endothelial dysfunction, and blood pressure increase in arterial hypertension. In our study, MyD88 propelled vascular inflammation driven by CD11b^+^ myelomonocytic cells independent of a specific TLR pathway. Selective expression of MyD88 in LysM^+^ cells reinstalled the phenotype of AngII-induced vascular injury, inflammation, and hypertension. In a prospective population-based cohort study, hypertensive individuals had a 20% increased risk for all-cause mortality when monocytes expressed high levels of MyD88 mRNA. Importantly, high expression of MyD88 in myeloid cells was strongly associated with chronic heart failure and may predict incident heart failure in individuals with arterial hypertension at baseline.

In our study, the absence of MyD88 attenuated arterial hypertension and vascular dysfunction in mice exposed to AngII infusion for 1 week (1 mg/kg/d). These protective effects are mediated by inhibiting the accumulation of inflammatory myelomonocytic cells in the vasculature. Our data indicate that MyD88 is important for cytokine production and AngII-induced differentiation of monocytes into an inflammatory phenotype. MyD88 deficient mice showed impaired infiltration with IFN-γ competent NK cells and reduced aortic *il-12p40* mRNA levels.^65^ MyD88-dependent IL-12 secretion by dendritic cells is critical for natural killer cell-mediated IFN-γ production and innate resistance to *Toxoplasma gondii*, and MyD88 may mediate signals within natural killer cells important for IL-12-dependent IFN-γ production and innate resistance to this parasite.^66^ Our data indicate that MyD88 deficiency interrupts the pro-inflammatory circuit of mutual activation of NK cells and myelomonocytic cells, which plays a particular role in AngII-induced vascular dysfunction.^34^

The activation mechanism of MyD88 through AngII remains unclear. MyD88 is also an adapter molecule necessary for all TLR3 and for IL-1 and IL-18 signalling.^67^ Chronic AngII elevation leads to oxidative stress and vascular inflammation. Oxidative stress and associated mitochondrial dysfunction in endothelial cells can trigger the activation of the NLRP3 inflammasome.^68^ NLRP3 inflammasome, a protein complex expressed by macrophages, triggers the immune system via pattern recognition receptors (PRR) and damage-activated molecular patterns (DAMPs)/pathogen-activated molecular patterns (PAMPs). It is regulated by reactive oxygen species.^69^ In AngII-treated mice, increased expression of NLRP3 and its downstream effectors (IL-1β, IL-18, caspase-1) has been detected in the vasculature, heart, and kidney and contributes to vascular inflammation and endothelial dysfunction. In turn, Nlrp3 deficiency reduced AngII-induced blood pressure elevation and endothelial dysfunction.^70^

In the context of our observations, MyD88-dependent signalling is important for the priming step of NLRP3 inflammasome activation. TLRs or IL1 receptors detect cytokines resulting in MyD88-dependent upstream activation of NFƘB pathway. Both MyD88 and NLRP3 can be considered to target AngII-induced vascular dysfunction.

MyD88 is critical for the TLR-dependent production of inflammatory cytokines. MyD88 and TLRs are pivotal in mediating cardiac injury following ischaemia/reperfusion.^71^ Under sterile conditions, TLRs are likely to be activated by endogenous ligands. Slight elevation of blood pressure and oxidative stress may generate neo-antigens and DAMPs or PAMPs, which can trigger TLRs on innate effector cells. Several studies provide evidence for endogenous TLR ligands released from damaged tissue like breakdown products of the extracellular matrix, heat shock proteins, or oxidized lipids.^72^

In our study, however, neither TLR4, TLR2, TLR7, nor TLR9 deficiency alone dampened AngII-induced vascular dysfunction, supporting a role of MyD88 beyond the above-mentioned vascular TLR-pathways.^73,74^ TLR-independent roles of MyD88 are, for example, the signalling via TACI (transmembrane activator) and CAML (calcium modulator and cyclophilin ligand) involved in B cell class-switching^74^ or the association of MyD88 with the IFN-γ receptor, resulting in the formation of a ‘signalosome’ which mediates the stabilization of IFN-γ induced cytokine and chemokine mRNA.^73^ MyD88 has also been implicated in early and late haematopoietic events that occur independently of the presence of an antigen.^75^

In line with our data, Owens *et al.* demonstrated that MyD88 deficiency reduced vascular injury and inflammation in a TLR2 and TLR4 independent mechanism in AngII-induced abdominal aortic aneurysm (AAA) formation, with the exact cell type driving these effects remaining unclear.^76^ Our data now clearly show that AngII-induced vascular injury is driven by MyD88 in myeloid cells with definite implications for the development of heart failure.

mRNA expression analysis of aortas and PBMCs of MyD88 deficient mice revealed an inhibition of the pro-inflammatory phenotypic switch of monocytes, which may contribute to partial protection from vascular injury. Singh *et al.*^77^ reported that arterial hypertension, cardiac hypertrophy, and pro-inflammatory gene expression in the heart and kidney in response to very high doses of AngII (4.32 mg/kg/d) were MyD88-independent. In contrast to that, and in accordance with our data, Wang *et al.* reported a reduction in cardiac hypertrophy in MyD88^−/−^ mice after 2 weeks of AngII infusion with 0.6 mg/kg/day, which is more comparable to our experimental protocol.^78^ These divergent findings indicate fine-tuned mechanisms of immune cell activation in hypertension that may be modulated by the release of different levels of endogenous ligands in response to varying levels of blood pressure hormones.

Using a mouse model of obesity-associated inflammatory disease, Yu *et al.*^79^ reported that deficiency of MyD88, specifically in myeloid cells, inhibited macrophage recruitment to adipose and arterial tissue and their switch to an M1-like phenotype, resulting in decreased susceptibility to atherosclerosis. Therefore, our study supports the original findings that MyD88 is a differentiation factor in myeloid precursors essential for both progressive and terminal differentiation.^11^  ^12^

Our BM transfer experiments revealed that the effects observed in global MyD88 deficient mice were at least in part mediated by BM-derived cells, reminiscent of earlier studies in which chimeric wild-type mice transplanted with MyD88 deficient BM cells showed reduced myocardial infarction sizes.^80,81^ The observed shift in aortic chimerism level to more cells of the recipient origin in mice that were reconstituted with MyD88 deficient haematopoietic cells (MyD88^−/−^→wt +AngII, *Figure 4*) could indicate a compensatory mechanism regulating the vascular immune response. Aortic lysates of MyD88^−/−^→wt +AngII mice showed a significant increase of mRNA expression of arterial macrophage marker *lyve-1*, which was slightly higher than other AngII-infused groups. The increasing population of these cells in the MyD88^+/+^ recipients partially antagonized the vascular protective effects transferred by the MyD88^−/−^ BM cells. These cells may represent resident cells’ progeny like self-renewing proliferating arterial macrophages.^35^ In summary, MyD88 drives both infiltrations of inflammatory cells into the aorta a cell proliferation and survival in our model of arterial hypertension.

MyD88 gain-of-function mutations are associated with malignancies in humans. The most frequent gain-of-function mutation L265P is found in over 90% of patients affected by Waldenström’s macroglobulinaemia, a malignancy affecting B cells.^21^ The mutation is linked with increased cell survival, nuclear factor kappa-light-chain-enhancer of activated B cell (NFκB) activity, and production of pro-inflammatory cytokines such as IL-6, IL-10, and IFN-β in human lymphoma.^22^ This brings up the intriguing notion that gain-of-function mutations of MyD88 might drive cardiovascular events in cardio-oncology. In our translational study, high monocytic MyD88 expression in combination with hypertension in individuals of the GHS study cohort nearly doubled the rate of all-cause mortality compared to hypertension alone. This complements our recent finding that length polymorphisms reducing heme oxygenase-1 expression in monocytes may increase mortality in the population, mainly by expanding the prevalence of the odd’s ratio for arterial hypertension.^32^ In the current study, we collected evidence, using computational analyses of the human protein interaction network, that MyD88 is significantly related to known risk genes for various hypertensive traits (*Figure 6*). Of note, the MyD88 protein is connected to the protein products of these genes by level-1 (direct) or level-2 interactions through proteins involved in various immune and stress response pathways that are all pathophysiologically meaningful in vascular inflammation. The reasons why there were no associations between markers of hypertension or vascular (dys)function with MyD88 expression in our study remain elusive. Putative explanations may be rooted in variations between self-reported history of hypertension and manifest hypertension at the time of assessing MyD88 mRNA expression, bidirectional control of expression with respect to individual circadian variations of blood pressure, and subclinical immune responses that cannot entirely be controlled for in the GHS. Future studies would have to address this issue, e.g. by providing information on longitudinal levels of MyD88 expression.

In summary, our study identifies MyD88 in myeloid cells as a key driver of vascular inflammation, endothelial dysfunction, and arterial hypertension. MyD88 deficiency reduces AngII-induced hypertension by limiting inflammatory monocyte accumulation. Clinically, high monocyte MyD88 expression is associated with increased mortality in individuals with hypertension. It was strongly associated with prevalent heart failure; the viability of MyD88 expression to predict incident heart failure remains to be corroborated in future studies. Beyond TLR signalling, MyD88 mediates immune activation, highlighting its role in cardiovascular disease and its potential as a therapeutic target for hypertension and heart failure.

## Supplementary Material

oeag031_Supplementary_Data
