# Supplementary material for: MyD88 in myeloid cells drives angiotensin II-induced vascular inflammation, is associated with prevalent heart failure, and predicts all-cause mortality in arterial hypertension
Source: Eur Heart J Open. 2026 Feb 27;6(2):oeag031. doi: 10.1093/ehjopen/oeag031 (PMC13080514; doi:10.1093/ehjopen/oeag031)
Supplement: oeag031_Supplementary_Data [file oeag031_supplementary_data.docx]

**Supplementary material**

**Material and methods**

**Reagents**: Chemicals and reagents of analytical grade were purchased from Sigma-Aldrich, Carl Roth or Merck.

**Animals and in vivo treatment protocol**: Animal studies were in accordance with the Guide for the Care and Use of Laboratory Animals as adopted and promulgated by the U.S. National Institutes of Health and approved by the Ethics Commission according to the German Law on the Protection of Animals (Landesuntersuchungsamt Rheinland-Pfalz, Koblenz, Germany: AZ 23 177-07/G11-1-02 and AZ 23-177-07/G15-1-051). MyD88^−/−^ ^1^, TLR2^−/−^ (B6.129-Tlr2^tm1Kir^/J, strain #004650 (The Jackson Laboratory)) ^2^, TLR4^−/− 3^, TLR7^−/− 4^, TLR9^−/− 5^ and Ly5.1 (B6.SJL*Ptprc^a^ Pepc^b^*/BoyJ, strain #002014 (The Jackson Laboratory)) mice all on a C57BL/6J background and C57BL/6J mice as corresponding wild-type controls were used. LysM^Cre/wt^MyD88^LSL/LSL^ mice that express MyD88 exclusively in lysozyme M positive cells were generated by crossing LysM^Cre 6^ mice to a conditional mutant mouse strain (MyD88^LSL/LSL^) ^7^ harboring a floxed transcriptional termination element between exons 1 and 2 of the MyD88 gene. MyD88^LSL/LSL^ littermates negative for LysM^Cre^ were used as controls. Mice were treated with AngII either for 7 d with 1 mg/kg/d or for 28 d with 0.7 mg/kg/d by subcutaneously implanted osmotic minipumps (model 1007D/2004, Alzet, Cupertino, CA) and killed under isoflurane anesthesia. Thereafter, the thoracic and abdominal aorta, mesenteric arteries, blood, bone marrow and spleen were collected for analysis.

**Vascular reactivity studies in conductance and resistance vessels**: To assess vasodilator properties of isolated vascular segments, aortas were cut into 3 mm segments and mounted on force transducers (Kent scientific corporation, Torrington, CT; Powerlab, ADInstruments, Spechbach, Germany) in organ chambers filled with Krebs-Henseleit solution (37°C, pH 7.35, containing 118 mmol/L NaCl, 4.69 mmol/L KCl, 1.87 mmol/L CaCl_2_, 1.2 mmol/L MgSO_4_, 1.03 mmol/L K_2_HPO_4_, 25 mmol/L NaHCO_3_, 11.1 mmol/L D-Glucose) bubbled with carbogen gas (95% O_2_, 5% CO_2_) and containing 1 μmol/L indomethacin to prevent endogenous synthesis of prostaglandins. Following precontraction with phenylephrine (0.15 μmol/L) or prostaglandin F_2α_ (3 nmol/L) to reach 50% to 80% of maximal tone induced by KCl, concentration-relaxation curves were recorded in response to increasing concentrations of the endothelium-dependent vasodilator acetylcholine (ACh, 1 nmol/L -3 μmol/L).

Second branches of mesenteric arteries were cut into 2 mm segments and mounted in a wire myograph (DMT620, Aarhus, Denmark) and vascular reactivity was measured as described ^8^. Acetylcholine-induced vasodilation was assessed on arteries pre-tensioned with phenylephrine. Nitric oxide (NO)-mediated vasodilation was measured in the presence of the cyclooxygenase inhibitor (diclofenac, 10 µmol/L) and Krebs-Henseleit solution containing 25mmol/L KCl to inhibit hyperpolarization.

**Blood pressure recordings**: C57BL/6, MyD88^-/-^, MyD88^LSL/LSL^, LysM^Cre/wt^MyD88^LSL/LSL^ and LysM^Cre/wt^ mice were equipped with carotid catheter implants for telemetric blood pressure measurements (TA-PA11C10, Data Science International (DSI), Tilburg, Netherlands). For anesthesia and analgesia mice received intraperitoneal injections of midazolam (5 mg/kg body weight; Ratiopharm GmbH, Ulm, Germany), medetomidine (0.5 mg/kg body weight; Pfizer Deutschland GmbH, Berlin, Germany), and fentanyl (0.05 mg/kg body weight; Janssen-Cilag GmbH, Neuss, Germany). After the surgical procedure, the animals were administered subcutaneously atipamezole (0.05 mg/kg) and flumazenil (0.01 mg/kg) to antagonize anesthesia. Postoperative analgesia was carried out with buprenorphine (0.075 mg/kg). The implantation of the catheters was performed under sterile conditions. After surgery, mice recovered for 1-2 weeks until the recording was started. Blood pressure was continuously recorded in freely moving animals using receiver platforms (DSI) for 2 weeks. First week with basal recording, second week with AngII infusion. In case of 4-week AngII infusion, pumps were implanted after 3 days of basal recording. Measurements were taken using the DataQuest system (DSI, Tilburg, Netherlands).

For non-invasive blood pressure measurements the tail cuff Coda Monitor System (Kent Scientific, Torrington, CT) was used. AngII and sham treated mice were measured 1 week after pump implantation.

**Quantification of mRNA levels:** For quantification of mRNA levels, RNA from snap-frozen aortas or isolated peripheral mononuclear cells was extracted by the modified guanidine isothiocyanate method of Chomczynski and Sacchi as applied previously in our group ^9^. Peripheral blood mononuclear cells (PBMCs) were isolated by 1077/1119 Histopaque double-gradient density centrifugation (Histopaque; Sigma-Aldrich, St. Louis, MO).

Quantitative real-time PCR (qRT-PCR) was performed on a Bio-Rad CFX96 real-time PCR detection system (BioRad, Munich, Germany). For qRT-PCR analysis 0.1 - 0.2 μg of total RNA were used with the QuantiTect Probe RT-PCR kit (Qiagen, Hilden, Germany). TaqMan Gene Expression assays were used as probe and primer sets (Applied Biosystems, Foster City, CA) for TATA-box binding protein (TBP, Mm00446973_m1), iNOS (Nos2, Mm00440485_m1), VCAM-1 (Mm00449197_m1), CD62L (L‑Sel, Mm00441291_m1), CX_3_CR1 (Mm02620111_s1), IL1-beta (Mm00434228_m1), TNF-alpha (Mm00443260_g1), Ccl2 (Mm00441242_m1), CD68 (Mm03047340_m1), IL12-p40 (Mm00434174_m1), Lyve1 (Mm00475056-m1), Hmox-1 (Mm00516005_m1), Nox1 (Mm00549170_m1) and Nox2 (Mm00432775_m1). Results were quantified with the comparative delta delta Ct method ^9^ and normalized to TBP.

**Flow cytometric analysis**: Mouse blood and aortic single-cell suspensions were analyzed as described previously ^10, 11^. Briefly, aortic vessels were cleaned of fatty tissue, minced and digested with collagenase II (1 mg/ml) and DNase I (50 μg/ml) or were indicated with liberase^TM^ (1 mg/ml, Roche, Basel, Switzerland) for 30 min at 37°C. By passing the lysed aortic fragments through a cell strainer (70 μm), a single-cell suspension was obtained. Single-cell suspensions were treated with Fc block, washed and surface-stained with CD45 APC-eFluor 780 (30-F11), NK1.1 PE-Cy7 (Pk136), CD11b PE (M1/70, BD Biosciences, San Jose, CA), Ly6G FITC (1A8, BD Biosciences, San Jose, CA), F4/80 APC (BM8), Ly6C PerCP-Cy.5.5 (AL-21) or for analysis of chimerism with CD45.1 (A20, BD Biosciences, San Jose, CA), CD45.2 (104), GR-1 V450 (RB6-8C5), CD11b PE-Cy7 (M1/70), F4/80 APC (BM8) and TCRβ-PE-Cy5 (H57-579) (all antibodies from eBioscience (San Diego, CA) unless stated otherwise). Dead cells were excluded by staining with Fixable Viability Dye eFluor506 (dead cell marker, eBioscience, San Diego, CA).

For detection of intracellular IFN-γ levels aortic lysates were ex vivo stimulated with 20 ng/ml IL-12 (R&D, Minneapolis, MN) and 20 ng/ml IL-18 (MBL, Aichi, Japan) for 4-5 hours. After 30 minutes 1.4 µl (1:10)/ 200 µl sample volume BD GolgiStop was added. After stimulation and surface staining, cells were fixed and stained for intracellular IFN-γ (eBioscience, San Diego, CA) with the BD Cytofix/Cytoperm Kit as indicated in the manufacturer’s instructions (BD Pharmingen, San Diego, CA).

Based on a live gate, events were acquired and analyzed using a BD FACS CANTO II flow cytometer (Becton Dickinson, Franklin Lakes, NJ) and FACS Diva software (Becton Dickinson, Franklin Lakes, NJ), respectively.

**Generation of BM chimera**: Bone marrow chimeras were generated by lethal irradiation of recipient C57BL/6 or MyD88^-/-^ mice with 9.5 Gy from Cs^137^ source (OB58-BA; Buchler, Braunschweig, Germany) and i.v. transfer of 5 × 10^6^ bone marrow cells harvested from femurs and tibias from animals of the indicated donor strain within 24 hours. One week before irradiation and during the first 2 weeks after transplantation, mice were given Borgal antibiotic (Hoechst Roussel Vet, Wiesbaden, Germany) in the drinking water. Mice were rested 7-8 weeks before use in experiments. Blood, aortic and bone marrow chimerism was verified by flow cytometric analysis using CD45.1 (A20, BD Biosciences, San Jose, CA) and CD45.2 (104, eBioscience, San Diego, CA).

Four experimental groups were included in the study: C57BL/6 wild type (wt) mice transplanted with wt BM and sham or AngII-treated (wtwt ±AngII), Ly5.1 wt mice transplanted with MyD88^-/-^ BM and infused with AngII (MyD88^-/-^wt +AngII) and MyD88^-/-^ mice reconstituted with Ly5.1 BM and infused with AngII (wtMyD88^-/-^ +AngII).

**Histology:** Aortas were harvested, and a 3 mm long ring from the distal half of descending thoracic aorta was fixed overnight in paraformaldehyde and embedded in paraffin wax. To examine the presence of perivascular fibrosis, 5 µm-thick cross sections were stained with a combination of Verhoeff`s elastic (VES) and Masson’s trichrome (MTC) stain to simultaneously visualize elastic fibers (black), muscular tissue (red) and extracellular matrix (blue). Images were taken on an Olympus BX51 microscope and morphometrically quantified using image analysis software (Image-Pro Plus; Media Cybernetics; version 7.0). To visualize interstitial collagen fibers, picro-sirius red staining was performed and sections photographed under polarized light. For both, results are expressed as µm² positive area per optical field at 200X magnification.

**Immunoflourescence staining**: Cryosections of aorta (8µm) were fixed with 4% paraformaldehyde, permeabilized with 0.25% Triton X-100, blocked with 5% BSA and immunostained with anti-CD68 (14-0681-82, eBioscience™) and anti-Nox2 (sc-130543, Santa Cruz) to determine infiltration of myelomonocytic cells and oxidative stress in vascular tissue. Fluorescence-labeled secondary antibodies (A11006, Invitrogen and 150116, Abcam) and 4′,6-diamidino-2-phenylindole (DAPI) were used together to stain cells nuclei and visualise immunostained cells with a fluorescence microscope.

Images were taken on fluorescence microscope (Keyence, Neu-Isenburg, Germany; model BZ-X810). Immunoflourescence staining was quantified by measuring the percentage of fluorescence area and surface area of the aorta section using Fiji/ Image J software (NIH). Section areas without signal were measured as background signal and substracted from the selected area of fluorescence. Data are expressed as percentage of immunopositive cells normalized to the surface area.

**Oxidative fluorescent microtopography:** Isolated aorta was cut into 3 mm rings, incubated in Krebs-Hepes-solution for 15 min at 37°C in the presence or absence of the eNOS inhibitor Nω-Nitro-L-arginine methyl ester (L-NAME, 10 µmol/L) and embedded in aluminum cups of about 1 ml of a polymeric resin (Tissue Tek®, Sakura Finetek, Alphen aan den Rijn, Netherlands) and frozen in liquid nitrogen. Cryosections (6 µm) were stained with the superoxide-sensitive dye dihydroethidium (DHE, 1µmol/L in PBS) and incubated for 30 min at 37°C. Green and red fluorescence was detected using a Zeiss Axiovert 40 CFL Camera (Zeiss, Oberkochen, Germany). Sections of all 4 study arms were analyzed in parallel with identical imaging parameters.

**NO measurement by electron paramagnetic resonance spectroscopy:** Aortic NO formation was measured using EPR-based spin trapping with iron-diethyldithiocarbamate [Fe(DETC)_2_] colloid as described in general previously ^12^. Whole aortas were cleaned of fat and connective tissue and cut into 3 mm rings. For iNOS stimulation aortic rings were incubated in RPMI containing 10% FCS +1 % PenStrep+ 10 μg/ml LPS (from E. coli, Sigma-Aldrich, Saint Louis, MO) for 19 - 24 h at 37°C, 5% CO_2_. LPS pre-treated or freshly prepared rings were transferred in 1 ml Krebs-Hepes buffer on a 24 well plate. In selected experiments 10 μmol/L iNOS inhibitor N-[3(aminomethyl)benzyl]-acetamidine, dihydrochloride (1400W) was added. For eNOS stimulation, samples were incubated with 10 μmol/L calcium ionophore (A23187) for 2 min on ice before the colloid solution was added. NaDETC (5.4 mg) and FeSO_4_·7H_2_O (3.4 mg) were separately dissolved under argon gas bubbling in two 15 ml volumes of ice-cold PBS with Ca^2+^/Mg^2+^. These solutions were rapidly mixed to obtain a colloid Fe(DETC)_2_ solution (0.4 mmol/L), which was added immediately to the rings (1 ml). After 60 min of incubation at 37°C, aortic rings were placed at a fixed position in a 1 ml syringe with removed top in Krebs-Hepes buffer and frozen in liquid nitrogen (in the way that the entire aortic sample was placed within a 100 µl volume of the syringe). For measurement, the frozen cylinder with the aortic sample was pressed out of the syringe and placed in a special Dewar vessel (Magnettech, Berlin, Germany) filled with liquid nitrogen. The localization of the aortic sample was adjusted to the middle of the resonator. EPR conditions: B_0_ = 3274 G, sweep = 110 G, sweep time = 60 s, modulation=7000mG, MW power=10mW, using a Miniscope MS400 (Magnettech, Berlin, Germany).

**Western Blot Analysis**: Isolated aortic tissue was cleaned of fatty tissue, shock-frozen and homogenized in liquid nitrogen. Protein was isolated like described previously ^13^. The tissue homogenates were adjusted for protein content, separated by SDS-PAGE, blotted onto a nitrocellulose membrane and blocked. Immunoblotting (Biorad, Hercules, CA) was performed with the following antibodies: monoclonal mouse anti α-actinin antibody (Sigma-Aldrich Seelze, Germany) as control for loading and transfer as well as polyclonal goat anti Nox1 antibody (Mox-1 H-15, BD Transduction Laboratories, Lexington, KY), monoclonal rabbit anti heme oxygenase-1 antibody (Epitomics, Burlingame, CA), monoclonal mouse anti Nox2 (53/gp91[phox], BD Transduction Laboratories, Lexington, KY), alpha-actinin (rabbit polyclonal, Sigma-Aldrich, Seelze, Germany). For detection, horseradish peroxidase-labelled secondary antibodies against mouse/rabbit (Vector Laboratories, Burlingame, CXA) together with ECL reagent (Amersham, Piscataway, NJ) were used. The ECL signal was detected with a ChemiLux Imager CsX-1400M (Intas, Göttingen, Germany) while bands were quantified with the Gel-Pro Analyzer software (Media Cybernetics, Bethesday, MD).

**In vivo microscopy of scull:** Total bone marrow cells were isolated from the femur of Ly5.1 (B6.SJL-Ptprca Pepcb/BoyJ; JAX stain #002014or MyD88 KO mice (B6.129P2(SJL)-MyD88tm1.1Defr/J; JAX strain #009088). The isolated cells were stained with CellTracker™ Orange CMTMR Dye (1µmol, #C2927 - Thermo Fisher Scientific) for 30 min at 37°C in a water bath, then washed with PBS. 1 mio stained bone marrow cells were then transplanted, intravenously, into non-irradiated Ly5.1 recipient mice. 4 hours (latency period) after the bone marrow transfer, the animals underwent an osmotic minipump (ALZET®, MODEL 1007D) implantation according to the protocol for the infusion of angiotensin II for 2-days (1 mg/d/kg body weight). The intra-vital microscopy (IVM) of the recipient mice was performed forty-eight hours post transplantation and angiotensin pump installation. Thirty minutes prior to performing the microscopy, mice were injected with fluorescein isothiocyanate-dextran (#46945, Sigma-Aldrich, St. Louis, MO) to visualize the vasculature. The mice were then anesthetized by xylene/ketamine and the scalp was removed. The calvarium was visualized using multi-photon intravital microscopy Leica MP DIVE, at 20X magnification in a 37 °C chamber (PMID: 32439895). The bone flouresces blue, while cells are visibly red, and the vasculature is stained green. The number of cells which had migrated from the vessels into the bone marrow cavity and the distance to the vasculature in the acquired images were then analyzed using ImageJ (Fiji) and Imaris software (Oxford Instruments Andor Ltd, Belfast, UK) ^14^.

**Gutenberg Health Study:** The Gutenberg Health Study (GHS) is designed as a population-based, prospective, observational, single-center cohort study in the Rhine-Main region in western mid-Germany and includes a total of 15,010 individuals ^15, 16^. The primary aim of the GHS is to evaluate and improve cardiovascular risk stratification. The sample was drawn randomly from the governmental local registry offices in the City of Mainz and the district of Mainz-Bingen. The sample was stratified 1:1 for sex and residence (urban and rural) and in equal strata for decades of age. Individuals between 35 and 74 years of age were enrolled, and written informed consent was obtained from all participants. Every participant underwent a comprehensive, standardized 5-hour clinical investigation. In addition to the clinical assessment, a large biobank has been established for biochemical and genetic analyses. All participants gave informed written consent to laboratory analyses, clinical examinations, sampling of biomaterial and the use of data records for research purposes. Details of the study protocol and the further purposes of the study are discussed elsewhere. Exclusion criteria were insufficient knowledge of German language and physical or psychological inability to participate in the examinations at the study centre. The study was designed according to the tenets of the revised Helsinki protocol and protocol and sampling design were approved by the local ethics committee and by the local and federal data safety commissioners.

**Blood pressure measurement within GHS:** Blood pressure was measured in each study participant in an upright sitting position (room temperature 22° centigrade) using the Omron 705CP-II (Omron, Japan) device and upper arm cuffs adapted to upper arm circumference (17-22cm, 22-32cm an 32-42cm, respectively). The upper part of the body was undressed and measurements were taken according to the following flow chart:

Application of

blood pressure cuff

1st

2nd

3rd measurement

5 Min.

3 Min.

3 Min.

The first measurement was taken on both the left and right arm. If differences between systolic and diastolic blood pressure between the two arms were below 20 mmHg or 15 mmHg, respectively, the 2^nd^ and 3^rd^ measurement were taken only on the left upper arm. For each individual, the mean of the 2^nd^ and 3^rd^ measurement was calculated and included in the study.

**Preparation peripheral blood mononuclear cells (PBMCs) and of PBMC mRNA:** Immediately following blood collection with BD Vacutainer® CPT™, the tubes were centrifuged at 22°C and 1850 x g for 20 min. Afterwards, PBMCs were washed twice in 2 mM EDTA-PBS, each centrifuged at 4°C and 500 g for 10 min. Cells were lysed in 1.5 ml TRIzol™ reagent (Life Technologies Corporation) and stored over night at -20°C. After thawing and homogenizing, samples were transferred in 2 ml Safe-Lock tubes and 0.3 ml of chloroform was added. The samples were mixed vigorously, incubated for 3 minutes at room temperature and then centrifuged at 12,000 × g for 15 min at 4°C. The RNA was precipitated from the aqueous phase by mixing with 0.7 ml of isopropanol. The samples were incubated at room temperature for 10 min and centrifuged at 12,000 × g for 10 min at RT. The supernatant was removed and the RNA pellet was washed once with 1.5 ml 75% ethanol. The pellet was air dried and dissolved in RNase-free water.

**Separation of monocytes and preparation of monocyte mRNA:** In the GHS, monocytes were separated within 60 min after blood collection and RNA extraction was performed at the same day. Isolation, purification and quality control procedure was described in detail previously ^17^.

**Gene expression associations:** In the GHS, transcriptome-wide gene expression profiles in monocytes and PBMCs were assessed using Illumina HT-12 v3 BeadChips (http://www.Illumina.com) ^18^. For Pre-processing of expression data, the summary probe-level data delivered by the Illumina scanner (mean and SD computed over all beads for a particular probe) was loaded in Beadstudio. The pre-processing done by the Illumina software, at the level of the scanner and by Beadstudio included: correction for local background effects, removal of outlier beads, computation of average bead signal and SD for each probe and gene, calculation of detection P-values using negative controls present on the array, quantile normalization across arrays, check of outlier samples using a clustering algorithm, check of positive controls. Analyses were carried out on the mean level for all probes in each gene. To stabilize variance across expression levels, we applied an arcsinh transformation to the expression data ^15^. Compared to a log transformation, this transformation has the advantage not to discard negative expression values which can occur in Illumina data. The Illumina HT-12 BeadChip included 37,804 genes (some probes being not assigned to RefSeq genes). A gene was declared significantly expressed in the dataset, i.e. expressed above background (as measured by the negative controls present on each array), when the detection P-value calculated by Beadstudio was < 0.05 in more than 5% of the samples. This resulted in 22,305 genes considered as being significantly expressed in our dataset. In total, 1,274 individuals with valid expression data were used for gene expression association analyses.

MyD88 or CD14 expression values are measured with Illumina HT-12 v3 as arbitrary fluorescence intensity (dimensionless) and represented as numerical intensity data for each probe. The final, processed values represent the unitless relative abundance or expression level of MyD88 or CD14 transcripts in the sample being analysed. RIN indicates RNA Integrity Number. It is a dimensionless quality control value of RNA samples without unit scored between 0 and 10. 10 indicates the most intact and the least degraded RNA. Values < 7 were excluded from the GHS study. Storage time reports time period [d] between RNA isolation and RNA amplification.

**Statistics:** Statistical analysis of the animal studies was performed with GraphPad Prism software (version 7; GraphPad Software, Inc., La Jolla, CA). Data were analyzed for normal distribution with the Kolmogorow–Smirnow test. When normal distribution was given, the 1‐way ANOVA test with Bonferroni post‐hoc test or 2‐way‐ANOVA were applied. If there was no normal distribution, Kruskal–Wallis test with Dunn's multiple comparison or comparison of selected columns were used as appropriate and indicated in the figure legends.

Data are presented as mean ± SEM; P values of <0.001, <0.01, and <0.05 were considered statistically significant and marked by 3, 2, and 1 asterisks, respectively.

In the population based cohort studies, linear, logistic and cox regression models as well as Kaplan–Meier analyses for cumulative incidence of all-cause mortality were performed. Values of p<0.05 were considered significant and exact p-values are given only in these cases; non-significant associations are not explicitly stated in the figures. Results of regression analyses are reported as hazard ratios (HR) with their 95 % confidence intervals (CIs). All statistical analyses within GHS were performed using R version 3.3.1 software (http://www.r-project.org).

**Computational analysis**: To investigate whether MyD88 preferentially interacts with known hypertension-associated genes in the human protein network, we considered loci reported in eight different publications using information from multiple genome-wide association studies (GWASs) or meta-analyses thereof: The Global BPgen consortium and the CHARGE consortium^19^; the International Consortium of Blood Pressure Genome-Wide Association Studies ^20^; the studies by Kato et al. and Ehret et al. reporting on the trans-ancestry genome-wide association and replication study of blood pressure phenotypes based und multiple GWAS ^21^ ^22^; the meta-analysis of Pattaro et al. based on multiple GWAS discovery and replication analyses ^23^; the GHS, KORA4F, SHIP-Trend and MESA Study ^24^; the 1000 Genomes study ^25^; and the METASTROKE, NIND-SiGN Consortium and International Stroke Genetics Consortium ^26^. Then, using the human protein interaction network from release 2 of the Human Integrative Protein Protein Interaction rEference (HIPPIE) ^27^, we computed the number of level 1 (direct interaction), level 2 (interaction with a neighbor of the target protein, i.e., a bridging protein) and bridging protein interactors between MyD88 and the gene products from each one of the GWAS candidate genes. Finally, we compared these counts with the number of level 1, level 2 and bridging protein interactors that MyD88 has with 1000 randomly sampled sets of gene products of the same size as in each GWAS and with the same degree (number of protein interactions) distribution. Reported p-values were calculated using a z-test, which compares each actual value with the mean of the corresponding random distribution (one-sample location test). If this difference is large, we reject the null hypothesis at the level of significance α = 0.05.

**Supplementary References**

1. Adachi O, Kawai T, Takeda K, Matsumoto M, Tsutsui H, Sakagami M, Nakanishi K, Akira S. Targeted disruption of the MyD88 gene results in loss of IL-1- and IL-18-mediated function. *Immunity*. 1998;9:143-150.

2. Wooten RM, Ma Y, Yoder RA, Brown JP, Weis JH, Zachary JF, Kirschning CJ, Weis JJ. Toll-like receptor 2 is required for innate, but not acquired, host defense to Borrelia burgdorferi. *Journal of immunology*. 2002;168:348-355.

3. Hoshino K, Takeuchi O, Kawai T, Sanjo H, Ogawa T, Takeda Y, Takeda K, Akira S. Cutting edge: Toll-like receptor 4 (TLR4)-deficient mice are hyporesponsive to lipopolysaccharide: evidence for TLR4 as the Lps gene product. *Journal of immunology*. 1999;162:3749-3752.

4. Hemmi H, Kaisho T, Takeuchi O, Sato S, Sanjo H, Hoshino K, Horiuchi T, Tomizawa H, Takeda K, Akira S. Small anti-viral compounds activate immune cells via the TLR7 MyD88-dependent signaling pathway. *Nat Immunol*. 2002;3:196-200.

5. Hemmi H, Takeuchi O, Kawai T, Kaisho T, Sato S, Sanjo H, Matsumoto M, Hoshino K, Wagner H, Takeda K, Akira S. A Toll-like receptor recognizes bacterial DNA. *Nature*. 2000;408:740-745.

6. Clausen BE, Burkhardt C, Reith W, Renkawitz R, Forster I. Conditional gene targeting in macrophages and granulocytes using LysMcre mice. *Transgenic Res*. 1999;8:265-277.

7. Gais P, Reim D, Jusek G, Rossmann-Bloeck T, Weighardt H, Pfeffer K, Altmayr F, Janssen KP, Holzmann B. Cutting edge: Divergent cell-specific functions of MyD88 for inflammatory responses and organ injury in septic peritonitis. *Journal of immunology*. 2012;188:5833-5837.

8. Randriamboavonjy V, Kyselova A, Elgheznawy A, Zukunft S, Wittig I, Fleming I. Calpain 1 cleaves and inactivates prostacyclin synthase in mesenteric arteries from diabetic mice. *Basic Res Cardiol*. 2017;112:10.

9. Livak KJ, Schmittgen TD. Analysis of relative gene expression data using real-time quantitative PCR and the 2(-Delta Delta C(T)) Method. *Methods*. 2001;25:402-408.

10. Wenzel P, Knorr M, Kossmann S, Stratmann J, Hausding M, Schuhmacher S, Karbach SH, Schwenk M, Yogev N, Schulz E, Oelze M, Grabbe S, Jonuleit H, Becker C, Daiber A, Waisman A, Munzel T. Lysozyme M-Positive Monocytes Mediate Angiotensin II-Induced Arterial Hypertension and Vascular Dysfunction. *Circulation*. 2011;124:1370-1381.

11. Kossmann S, Schwenk M, Hausding M, Karbach SH, Schmidgen MI, Brandt M, Knorr M, Hu H, Kroller-Schon S, Schonfelder T, Grabbe S, Oelze M, Daiber A, Munzel T, Becker C, Wenzel P. Angiotensin II-Induced Vascular Dysfunction Depends on Interferon-gamma-Driven Immune Cell Recruitment and Mutual Activation of Monocytes and NK-Cells. *Arterioscler Thromb Vasc Biol*. 2013;33:1313-1319.

12. Kossmann S, Hu H, Steven S, Schonfelder T, Fraccarollo D, Mikhed Y, Brahler M, Knorr M, Brandt M, Karbach SH, Becker C, Oelze M, Bauersachs J, Widder J, Munzel T, Daiber A, Wenzel P. Inflammatory Monocytes Determine Endothelial Nitric-oxide Synthase Uncoupling and Nitro-oxidative Stress Induced by Angiotensin II. *The Journal of biological chemistry*. 2014;289:27540-27550.

13. Schuhmacher S, Foretz M, Knorr M, Jansen T, Hortmann M, Wenzel P, Oelze M, Kleschyov AL, Daiber A, Keaney JF, Jr., Wegener G, Lackner K, Munzel T, Viollet B, Schulz E. alpha1AMP-activated protein kinase preserves endothelial function during chronic angiotensin II treatment by limiting Nox2 upregulation. *Arterioscler Thromb Vasc Biol*. 2011;31:560-566.

14. Zanetti C, Kumar R, Ender J, Godavarthy PS, Hartmann M, Hey J, Breuer K, Weissenberger ES, Minciacchi VR, Karantanou C, Gu Z, Roberts KG, Metzler M, Stock W, Mullighan CG, Bloomfield CD, Filmann N, Bankov K, Hartmann S, Hasserjian RP, Cousins AF, Halsey C, Plass C, Lipka DB, Krause DS. The age of the bone marrow microenvironment influences B-cell acute lymphoblastic leukemia progression via CXCR5-CXCL13. Blood 2021;138:1870–1884.

15. Zeller T, Wild P, Szymczak S, Rotival M, Schillert A, Castagne R, Maouche S, Germain M, Lackner K, Rossmann H, Eleftheriadis M, Sinning CR, Schnabel RB, Lubos E, Mennerich D, Rust W, Perret C, Proust C, Nicaud V, Loscalzo J, Hubner N, Tregouet D, Munzel T, Ziegler A, Tiret L, Blankenberg S, Cambien F. Genetics and beyond - the transcriptome of human monocytes and disease susceptibility. *PLoS One*. 2010;5:e10693.

16. Wild PS, Zeller T, Beutel M, Blettner M, Dugi KA, Lackner KJ, Pfeiffer N, Munzel T, Blankenberg S. [The Gutenberg Health Study]. *Bundesgesundheitsblatt, Gesundheitsforschung, Gesundheitsschutz*. 2012;55:824-829.

17. Wenzel P, Rossmann H, Muller C, Kossmann S, Oelze M, Schulz A, Arnold N, Simsek C, Lagrange J, Klemz R, Schonfelder T, Brandt M, Karbach SH, Knorr M, Finger S, Neukirch C, Hauser F, Beutel ME, Kroller-Schon S, Schulz E, Schnabel RB, Lackner K, Wild PS, Zeller T, Daiber A, Blankenberg S, Munzel T. Heme oxygenase-1 suppresses a pro-inflammatory phenotype in monocytes and determines endothelial function and arterial hypertension in mice and humans. *European heart journal*. 2015;36:3437-3446.

18. Schurmann C, Heim K, Schillert A, Blankenberg S, Carstensen M, Dörr M, Endlich K, Felix SB, Gieger C, Grallert H, Herder C, Hoffmann W, Homuth G, Illig T, Kruppa J, Meitinger T, Muller C, Nauck M, Peters A, Rettig R, Roden M, Strauch K, Volker U, Völzke H, Wahl S, Wallaschofski H, Wild PS, Zeller T, Teumer A, Prokisch H, Ziegler A. Analyzing Illumina Gene Expression Microarray Data from Different Tissues: Methodological Aspects of Data Analysis in the MetaXpress Consortium. *PLoS One*. 2012;7:e50938.

19. Newton-Cheh C, Larson MG, Vasan RS, Levy D, Bloch KD, Surti A, Guiducci C, Kathiresan S, Benjamin EJ, Struck J, Morgenthaler NG, Bergmann A, Blankenberg S, Kee F, Nilsson P, Yin X, Peltonen L, Vartiainen E, Salomaa V, Hirschhorn JN, Melander O, Wang TJ. Association of common variants in NPPA and NPPB with circulating natriuretic peptides and blood pressure. *Nat Genet*. 2009;41:348-353.

20. Wain LV, Verwoert GC, O'Reilly PF, Shi G, Johnson T, Johnson AD, Bochud M, Rice KM, Henneman P, Smith AV, Ehret GB, Amin N, Larson MG, Mooser V, Hadley D, Dorr M, Bis JC, Aspelund T, Esko T, Janssens AC, Zhao JH, Heath S, Laan M, Fu J, Pistis G, Luan J, Arora P, Lucas G, Pirastu N, Pichler I, Jackson AU, Webster RJ, Zhang F, Peden JF, Schmidt H, Tanaka T, Campbell H, Igl W, Milaneschi Y, Hottenga JJ, Vitart V, Chasman DI, Trompet S, Bragg-Gresham JL, Alizadeh BZ, Chambers JC, Guo X, Lehtimaki T, Kuhnel B, Lopez LM, Polasek O, Boban M, Nelson CP, Morrison AC, Pihur V, Ganesh SK, Hofman A, Kundu S, Mattace-Raso FU, Rivadeneira F, Sijbrands EJ, Uitterlinden AG, Hwang SJ, Vasan RS, Wang TJ, Bergmann S, Vollenweider P, Waeber G, Laitinen J, Pouta A, Zitting P, McArdle WL, Kroemer HK, Volker U, Volzke H, Glazer NL, Taylor KD, Harris TB, Alavere H, Haller T, Keis A, Tammesoo ML, Aulchenko Y, Barroso I, Khaw KT, Galan P, Hercberg S, Lathrop M, Eyheramendy S, Org E, Sober S, Lu X, Nolte IM, Penninx BW, Corre T, Masciullo C, Sala C, Groop L, Voight BF, Melander O, O'Donnell CJ, Salomaa V, d'Adamo AP, Fabretto A, Faletra F, Ulivi S, Del Greco F, Facheris M, Collins FS, Bergman RN, Beilby JP, Hung J, Musk AW, Mangino M, Shin SY, Soranzo N, Watkins H, Goel A, Hamsten A, Gider P, Loitfelder M, Zeginigg M, Hernandez D, Najjar SS, Navarro P, Wild SH, Corsi AM, Singleton A, de Geus EJ, Willemsen G, Parker AN, Rose LM, Buckley B, Stott D, Orru M, Uda M, LifeLines Cohort S, van der Klauw MM, Zhang W, Li X, Scott J, Chen YD, Burke GL, Kahonen M, Viikari J, Doring A, Meitinger T, Davies G, Starr JM, Emilsson V, Plump A, Lindeman JH, Hoen PA, Konig IR, EchoGen c, Felix JF, Clarke R, Hopewell JC, Ongen H, Breteler M, Debette S, Destefano AL, Fornage M, AortaGen C, Mitchell GF, Group CCHFW, Smith NL, KidneyGen c, Holm H, Stefansson K, Thorleifsson G, Thorsteinsdottir U, consortium CK, Cardiogenics c, CardioGram, Samani NJ, Preuss M, Rudan I, Hayward C, Deary IJ, Wichmann HE, Raitakari OT, Palmas W, Kooner JS, Stolk RP, Jukema JW, Wright AF, Boomsma DI, Bandinelli S, Gyllensten UB, Wilson JF, Ferrucci L, Schmidt R, Farrall M, Spector TD, Palmer LJ, Tuomilehto J, Pfeufer A, Gasparini P, Siscovick D, Altshuler D, Loos RJ, Toniolo D, Snieder H, Gieger C, Meneton P, Wareham NJ, Oostra BA, Metspalu A, Launer L, Rettig R, Strachan DP, Beckmann JS, Witteman JC, Erdmann J, van Dijk KW, Boerwinkle E, Boehnke M, Ridker PM, Jarvelin MR, Chakravarti A, Abecasis GR, Gudnason V, Newton-Cheh C, Levy D, Munroe PB, Psaty BM, Caulfield MJ, Rao DC, Tobin MD, Elliott P, van Duijn CM. Genome-wide association study identifies six new loci influencing pulse pressure and mean arterial pressure. *Nat Genet*. 2011;43:1005-1011.

21. Kato N, Loh M, Takeuchi F, Verweij N, Wang X, Zhang W, Kelly TN, Saleheen D, Lehne B, Leach IM, Drong AW, Abbott J, Wahl S, Tan ST, Scott WR, Campanella G, Chadeau-Hyam M, Afzal U, Ahluwalia TS, Bonder MJ, Chen P, Dehghan A, Edwards TL, Esko T, Go MJ, Harris SE, Hartiala J, Kasela S, Kasturiratne A, Khor CC, Kleber ME, Li H, Yu Mok Z, Nakatochi M, Sapari NS, Saxena R, Stewart AFR, Stolk L, Tabara Y, Teh AL, Wu Y, Wu JY, Zhang Y, Aits I, Da Silva Couto Alves A, Das S, Dorajoo R, Hopewell JC, Kim YK, Koivula RW, Luan J, Lyytikainen LP, Nguyen QN, Pereira MA, Postmus I, Raitakari OT, Bryan MS, Scott RA, Sorice R, Tragante V, Traglia M, White J, Yamamoto K, Zhang Y, Adair LS, Ahmed A, Akiyama K, Asif R, Aung T, Barroso I, Bjonnes A, Braun TR, Cai H, Chang LC, Chen CH, Cheng CY, Chong YS, Collins R, Courtney R, Davies G, Delgado G, Do LD, Doevendans PA, Gansevoort RT, Gao YT, Grammer TB, Grarup N, Grewal J, Gu D, Wander GS, Hartikainen AL, Hazen SL, He J, Heng CK, Hixson JE, Hofman A, Hsu C, Huang W, Husemoen LLN, Hwang JY, Ichihara S, Igase M, Isono M, Justesen JM, Katsuya T, Kibriya MG, Kim YJ, Kishimoto M, Koh WP, Kohara K, Kumari M, Kwek K, Lee NR, Lee J, Liao J, Lieb W, Liewald DCM, Matsubara T, Matsushita Y, Meitinger T, Mihailov E, Milani L, Mills R, Mononen N, Muller-Nurasyid M, Nabika T, Nakashima E, Ng HK, Nikus K, Nutile T, Ohkubo T, Ohnaka K, Parish S, Paternoster L, Peng H, Peters A, Pham ST, Pinidiyapathirage MJ, Rahman M, Rakugi H, Rolandsson O, Ann Rozario M, Ruggiero D, Sala CF, Sarju R, Shimokawa K, Snieder H, Sparso T, Spiering W, Starr JM, Stott DJ, Stram DO, Sugiyama T, Szymczak S, Tang WHW, Tong L, Trompet S, Turjanmaa V, Ueshima H, Uitterlinden AG, Umemura S, Vaarasmaki M, van Dam RM, van Gilst WH, van Veldhuisen DJ, Viikari JS, Waldenberger M, Wang Y, Wang A, Wilson R, Wong TY, Xiang YB, Yamaguchi S, Ye X, Young RD, Young TL, Yuan JM, Zhou X, Asselbergs FW, Ciullo M, Clarke R, Deloukas P, Franke A, Franks PW, Franks S, Friedlander Y, Gross MD, Guo Z, Hansen T, Jarvelin MR, Jorgensen T, Jukema JW, Kahonen M, Kajio H, Kivimaki M, Lee JY, Lehtimaki T, Linneberg A, Miki T, Pedersen O, Samani NJ, Sorensen TIA, Takayanagi R, Toniolo D, consortium B, GRAMplusCD CA, LifeLines Cohort S, InterAct C, Ahsan H, Allayee H, Chen YT, Danesh J, Deary IJ, Franco OH, Franke L, Heijman BT, Holbrook JD, Isaacs A, Kim BJ, Lin X, Liu J, Marz W, Metspalu A, Mohlke KL, Sanghera DK, Shu XO, van Meurs JBJ, Vithana E, Wickremasinghe AR, Wijmenga C, Wolffenbuttel BHW, Yokota M, Zheng W, Zhu D, Vineis P, Kyrtopoulos SA, Kleinjans JCS, McCarthy MI, Soong R, Gieger C, Scott J, Teo YY, He J, Elliott P, Tai ES, van der Harst P, Kooner JS, Chambers JC. Trans-ancestry genome-wide association study identifies 12 genetic loci influencing blood pressure and implicates a role for DNA methylation. *Nat Genet*. 2015;47:1282-1293.

22. Ehret GB, Ferreira T, Chasman DI, Jackson AU, Schmidt EM, Johnson T, Thorleifsson G, Luan J, Donnelly LA, Kanoni S, Petersen AK, Pihur V, Strawbridge RJ, Shungin D, Hughes MF, Meirelles O, Kaakinen M, Bouatia-Naji N, Kristiansson K, Shah S, Kleber ME, Guo X, Lyytikainen LP, Fava C, Eriksson N, Nolte IM, Magnusson PK, Salfati EL, Rallidis LS, Theusch E, Smith AJP, Folkersen L, Witkowska K, Pers TH, Joehanes R, Kim SK, Lataniotis L, Jansen R, Johnson AD, Warren H, Kim YJ, Zhao W, Wu Y, Tayo BO, Bochud M, consortium CH-E, consortium C-H, Wellcome Trust Case Control C, Absher D, Adair LS, Amin N, Arking DE, Axelsson T, Baldassarre D, Balkau B, Bandinelli S, Barnes MR, Barroso I, Bevan S, Bis JC, Bjornsdottir G, Boehnke M, Boerwinkle E, Bonnycastle LL, Boomsma DI, Bornstein SR, Brown MJ, Burnier M, Cabrera CP, Chambers JC, Chang IS, Cheng CY, Chines PS, Chung RH, Collins FS, Connell JM, Doring A, Dallongeville J, Danesh J, de Faire U, Delgado G, Dominiczak AF, Doney ASF, Drenos F, Edkins S, Eicher JD, Elosua R, Enroth S, Erdmann J, Eriksson P, Esko T, Evangelou E, Evans A, Fall T, Farrall M, Felix JF, Ferrieres J, Ferrucci L, Fornage M, Forrester T, Franceschini N, Duran OHF, Franco-Cereceda A, Fraser RM, Ganesh SK, Gao H, Gertow K, Gianfagna F, Gigante B, Giulianini F, Goel A, Goodall AH, Goodarzi MO, Gorski M, Grassler J, Groves C, Gudnason V, Gyllensten U, Hallmans G, Hartikainen AL, Hassinen M, Havulinna AS, Hayward C, Hercberg S, Herzig KH, Hicks AA, Hingorani AD, Hirschhorn JN, Hofman A, Holmen J, Holmen OL, Hottenga JJ, Howard P, Hsiung CA, Hunt SC, Ikram MA, Illig T, Iribarren C, Jensen RA, Kahonen M, Kang H, Kathiresan S, Keating BJ, Khaw KT, Kim YK, Kim E, Kivimaki M, Klopp N, Kolovou G, Komulainen P, Kooner JS, Kosova G, Krauss RM, Kuh D, Kutalik Z, Kuusisto J, Kvaloy K, Lakka TA, Lee NR, Lee IT, Lee WJ, Levy D, Li X, Liang KW, Lin H, Lin L, Lindstrom J, Lobbens S, Mannisto S, Muller G, Muller-Nurasyid M, Mach F, Markus HS, Marouli E, McCarthy MI, McKenzie CA, Meneton P, Menni C, Metspalu A, Mijatovic V, Moilanen L, Montasser ME, Morris AD, Morrison AC, Mulas A, Nagaraja R, Narisu N, Nikus K, O'Donnell CJ, O'Reilly PF, Ong KK, Paccaud F, Palmer CD, Parsa A, Pedersen NL, Penninx BW, Perola M, Peters A, Poulter N, Pramstaller PP, Psaty BM, Quertermous T, Rao DC, Rasheed A, Rayner N, Renstrom F, Rettig R, Rice KM, Roberts R, Rose LM, Rossouw J, Samani NJ, Sanna S, Saramies J, Schunkert H, Sebert S, Sheu WH, Shin YA, Sim X, Smit JH, Smith AV, Sosa MX, Spector TD, Stancakova A, Stanton A, Stirrups KE, Stringham HM, Sundstrom J, Swift AJ, Syvanen AC, Tai ES, Tanaka T, Tarasov KV, Teumer A, Thorsteinsdottir U, Tobin MD, Tremoli E, Uitterlinden AG, Uusitupa M, Vaez A, Vaidya D, van Duijn CM, van Iperen EPA, Vasan RS, Verwoert GC, Virtamo J, Vitart V, Voight BF, Vollenweider P, Wagner A, Wain LV, Wareham NJ, Watkins H, Weder AB, Westra HJ, Wilks R, Wilsgaard T, Wilson JF, Wong TY, Yang TP, Yao J, Yengo L, Zhang W, Zhao JH, Zhu X, Bovet P, Cooper RS, Mohlke KL, Saleheen D, Lee JY, Elliott P, Gierman HJ, Willer CJ, Franke L, Hovingh GK, Taylor KD, Dedoussis G, Sever P, Wong A, Lind L, Assimes TL, Njolstad I, Schwarz PE, Langenberg C, Snieder H, Caulfield MJ, Melander O, Laakso M, Saltevo J, Rauramaa R, Tuomilehto J, Ingelsson E, Lehtimaki T, Hveem K, Palmas W, Marz W, Kumari M, Salomaa V, Chen YI, Rotter JI, Froguel P, Jarvelin MR, Lakatta EG, Kuulasmaa K, Franks PW, Hamsten A, Wichmann HE, Palmer CNA, Stefansson K, Ridker PM, Loos RJF, Chakravarti A, Deloukas P, Morris AP, Newton-Cheh C, Munroe PB. The genetics of blood pressure regulation and its target organs from association studies in 342,415 individuals. *Nat Genet*. 2016;48:1171-1184.

23. Pattaro C, Teumer A, Gorski M, Chu AY, Li M, Mijatovic V, Garnaas M, Tin A, Sorice R, Li Y, Taliun D, Olden M, Foster M, Yang Q, Chen MH, Pers TH, Johnson AD, Ko YA, Fuchsberger C, Tayo B, Nalls M, Feitosa MF, Isaacs A, Dehghan A, d'Adamo P, Adeyemo A, Dieffenbach AK, Zonderman AB, Nolte IM, van der Most PJ, Wright AF, Shuldiner AR, Morrison AC, Hofman A, Smith AV, Dreisbach AW, Franke A, Uitterlinden AG, Metspalu A, Tonjes A, Lupo A, Robino A, Johansson A, Demirkan A, Kollerits B, Freedman BI, Ponte B, Oostra BA, Paulweber B, Kramer BK, Mitchell BD, Buckley BM, Peralta CA, Hayward C, Helmer C, Rotimi CN, Shaffer CM, Muller C, Sala C, van Duijn CM, Saint-Pierre A, Ackermann D, Shriner D, Ruggiero D, Toniolo D, Lu Y, Cusi D, Czamara D, Ellinghaus D, Siscovick DS, Ruderfer D, Gieger C, Grallert H, Rochtchina E, Atkinson EJ, Holliday EG, Boerwinkle E, Salvi E, Bottinger EP, Murgia F, Rivadeneira F, Ernst F, Kronenberg F, Hu FB, Navis GJ, Curhan GC, Ehret GB, Homuth G, Coassin S, Thun GA, Pistis G, Gambaro G, Malerba G, Montgomery GW, Eiriksdottir G, Jacobs G, Li G, Wichmann HE, Campbell H, Schmidt H, Wallaschofski H, Volzke H, Brenner H, Kroemer HK, Kramer H, Lin H, Leach IM, Ford I, Guessous I, Rudan I, Prokopenko I, Borecki I, Heid IM, Kolcic I, Persico I, Jukema JW, Wilson JF, Felix JF, Divers J, Lambert JC, Stafford JM, Gaspoz JM, Smith JA, Faul JD, Wang JJ, Ding J, Hirschhorn JN, Attia J, Whitfield JB, Chalmers J, Viikari J, Coresh J, Denny JC, Karjalainen J, Fernandes JK, Endlich K, Butterbach K, Keene KL, Lohman K, Portas L, Launer LJ, Lyytikainen LP, Yengo L, Franke L, Ferrucci L, Rose LM, Kedenko L, Rao M, Struchalin M, Kleber ME, Cavalieri M, Haun M, Cornelis MC, Ciullo M, Pirastu M, de Andrade M, McEvoy MA, Woodward M, Adam M, Cocca M, Nauck M, Imboden M, Waldenberger M, Pruijm M, Metzger M, Stumvoll M, Evans MK, Sale MM, Kahonen M, Boban M, Bochud M, Rheinberger M, Verweij N, Bouatia-Naji N, Martin NG, Hastie N, Probst-Hensch N, Soranzo N, Devuyst O, Raitakari O, Gottesman O, Franco OH, Polasek O, Gasparini P, Munroe PB, Ridker PM, Mitchell P, Muntner P, Meisinger C, Smit JH, Consortium I, Consortium A, Cardiogram, Group CH-HF, Consortium EC, Kovacs P, Wild PS, Froguel P, Rettig R, Magi R, Biffar R, Schmidt R, Middelberg RP, Carroll RJ, Penninx BW, Scott RJ, Katz R, Sedaghat S, Wild SH, Kardia SL, Ulivi S, Hwang SJ, Enroth S, Kloiber S, Trompet S, Stengel B, Hancock SJ, Turner ST, Rosas SE, Stracke S, Harris TB, Zeller T, Zemunik T, Lehtimaki T, Illig T, Aspelund T, Nikopensius T, Esko T, Tanaka T, Gyllensten U, Volker U, Emilsson V, Vitart V, Aalto V, Gudnason V, Chouraki V, Chen WM, Igl W, Marz W, Koenig W, Lieb W, Loos RJ, Liu Y, Snieder H, Pramstaller PP, Parsa A, O'Connell JR, Susztak K, Hamet P, Tremblay J, de Boer IH, Boger CA, Goessling W, Chasman DI, Kottgen A, Kao WH, Fox CS. Genetic associations at 53 loci highlight cell types and biological pathways relevant for kidney function. *Nat Commun*. 2016;7:10023.

24. Zeller T, Schurmann C, Schramm K, Muller C, Kwon S, Wild PS, Teumer A, Herrington D, Schillert A, Iacoviello L, Kratzer A, Jagodzinski A, Karakas M, Ding J, Neumann JT, Kuulasmaa K, Gieger C, Kacprowski T, Schnabel RB, Roden M, Wahl S, Rotter JI, Ojeda F, Carstensen-Kirberg M, Tregouet DA, Dorr M, Meitinger T, Lackner KJ, Wolf P, Felix SB, Landmesser U, Costanzo S, Ziegler A, Liu Y, Volker U, Palmas W, Prokisch H, Guo X, Herder C, Blankenberg S, Homuth G. Transcriptome-Wide Analysis Identifies Novel Associations With Blood Pressure. *Hypertension*. 2017;70:743-750.

25. Wain LV, Vaez A, Jansen R, Joehanes R, van der Most PJ, Erzurumluoglu AM, O'Reilly PF, Cabrera CP, Warren HR, Rose LM, Verwoert GC, Hottenga JJ, Strawbridge RJ, Esko T, Arking DE, Hwang SJ, Guo X, Kutalik Z, Trompet S, Shrine N, Teumer A, Ried JS, Bis JC, Smith AV, Amin N, Nolte IM, Lyytikainen LP, Mahajan A, Wareham NJ, Hofer E, Joshi PK, Kristiansson K, Traglia M, Havulinna AS, Goel A, Nalls MA, Sober S, Vuckovic D, Luan J, Del Greco MF, Ayers KL, Marrugat J, Ruggiero D, Lopez LM, Niiranen T, Enroth S, Jackson AU, Nelson CP, Huffman JE, Zhang W, Marten J, Gandin I, Harris SE, Zemunik T, Lu Y, Evangelou E, Shah N, de Borst MH, Mangino M, Prins BP, Campbell A, Li-Gao R, Chauhan G, Oldmeadow C, Abecasis G, Abedi M, Barbieri CM, Barnes MR, Batini C, Beilby J, Blake T, Boehnke M, Bottinger EP, Braund PS, Brown M, Brumat M, Campbell H, Chambers JC, Cocca M, Collins F, Connell J, Cordell HJ, Damman JJ, Davies G, de Geus EJ, de Mutsert R, Deelen J, Demirkale Y, Doney ASF, Dorr M, Farrall M, Ferreira T, Franberg M, Gao H, Giedraitis V, Gieger C, Giulianini F, Gow AJ, Hamsten A, Harris TB, Hofman A, Holliday EG, Hui J, Jarvelin MR, Johansson A, Johnson AD, Jousilahti P, Jula A, Kahonen M, Kathiresan S, Khaw KT, Kolcic I, Koskinen S, Langenberg C, Larson M, Launer LJ, Lehne B, Liewald DCM, Lin L, Lind L, Mach F, Mamasoula C, Menni C, Mifsud B, Milaneschi Y, Morgan A, Morris AD, Morrison AC, Munson PJ, Nandakumar P, Nguyen QT, Nutile T, Oldehinkel AJ, Oostra BA, Org E, Padmanabhan S, Palotie A, Pare G, Pattie A, Penninx B, Poulter N, Pramstaller PP, Raitakari OT, Ren M, Rice K, Ridker PM, Riese H, Ripatti S, Robino A, Rotter JI, Rudan I, Saba Y, Saint Pierre A, Sala CF, Sarin AP, Schmidt R, Scott R, Seelen MA, Shields DC, Siscovick D, Sorice R, Stanton A, Stott DJ, Sundstrom J, Swertz M, Taylor KD, Thom S, Tzoulaki I, Tzourio C, Uitterlinden AG, Volker U, Vollenweider P, Wild S, Willemsen G, Wright AF, Yao J, Theriault S, Conen D, Attia J, Sever P, Debette S, Mook-Kanamori DO, Zeggini E, Spector TD, van der Harst P, Palmer CNA, Vergnaud AC, Loos RJF, Polasek O, Starr JM, Girotto G, Hayward C, Kooner JS, Lindgren CM, Vitart V, Samani NJ, Tuomilehto J, Gyllensten U, Knekt P, Deary IJ, Ciullo M, Elosua R, Keavney BD, Hicks AA, Scott RA, Gasparini P, Laan M, Liu Y, Watkins H, Hartman CA, Salomaa V, Toniolo D, Perola M, Wilson JF, Schmidt H, Zhao JH, Lehtimaki T, van Duijn CM, Gudnason V, Psaty BM, Peters A, Rettig R, James A, Jukema JW, Strachan DP, Palmas W, Metspalu A, Ingelsson E, Boomsma DI, Franco OH, Bochud M, Newton-Cheh C, Munroe PB, Elliott P, Chasman DI, Chakravarti A, Knight J, Morris AP, Levy D, Tobin MD, Snieder H, Caulfield MJ, Ehret GB. Novel Blood Pressure Locus and Gene Discovery Using Genome-Wide Association Study and Expression Data Sets From Blood and the Kidney. *Hypertension*. epub ahead of print july 24th 2017; doi: 10.1161/HYPERTENSIONAHA.117.09438

26. Phuah CL, Dave T, Malik R, Raffeld MR, Ayres AM, Goldstein JN, Viswanathan A, Greenberg SM, Jagiella JM, Hansen BM, Norrving B, Jimenez-Conde J, Roquer J, Pichler A, Enzinger C, Montaner J, Fernandez-Cadenas I, Lindgren A, Slowik A, Schmidt R, Biffi A, Rost N, Langefeld CD, Markus HS, Mitchell BD, Worrall BB, Kittner SJ, Woo D, Dichgans M, Rosand J, Anderson CD, Metastroke, Consortium NI-S, International Stroke Genetics C. Genetic variants influencing elevated myeloperoxidase levels increase risk of stroke. *Brain*. 2017;140:2663-2672.

27. Alanis-Lobato G, Andrade-Navarro MA, Schaefer MH. HIPPIE v2.0: enhancing meaningfulness and reliability of protein-protein interaction networks. *Nucleic Acids Res*. 2017;45:D408-D414.

**Supplementary figures and tables**


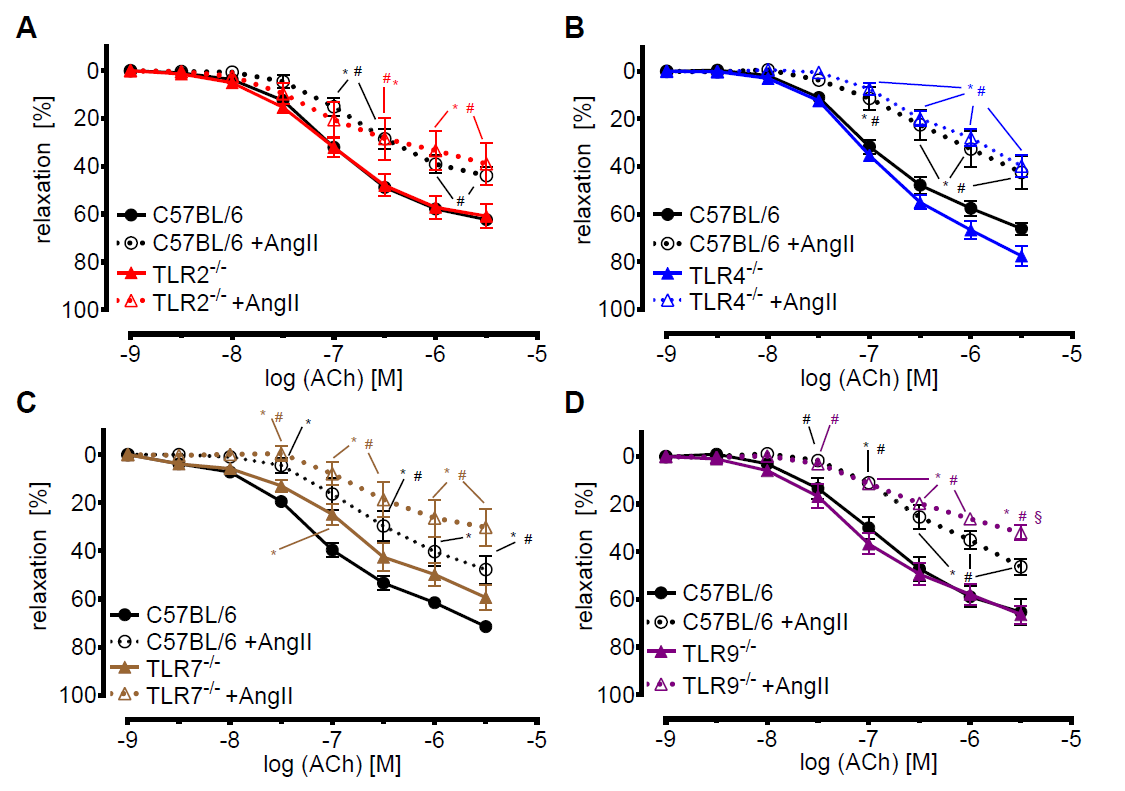


**Supplementary Figure 1: Genetic deletion of single TLRs is not equivalent to MyD88 deficiency with regards to endothelial (dys)function.** Relaxation of aortic ring segments of sham or AngII-infused C57BL/6, TLR2^-/-^ (A), TLR4^-/-^ (B), TLR7^-/-^ (C) or TLR9^-/-^ (D) mice in response to the endothelium dependent vasodilator (ACh) was measured by isometric tension recordings. Data are mean±SEM of n=8-10 (TLR4^-/-^), n=6-7 (TLR2^-/-^) or n=3-4 (TLR7^-/-^ and TLR9^-/-^) animals per group. Ordinary One-way ANOVA (TLR4^-/-^) or Kruskal Wallis test (TLR2^-/-^, TLR7^-/-^, TLR9^-/-^) of maximal relaxation; *, P<0.05 vs. C57BL/6; #, P<0.05 vs. TLR4^-/-^, TLR2^-/-^, TLR7^-/-^ or TLR9^-/-^; §, P<0.05 vs C57BL/6 +AngII.


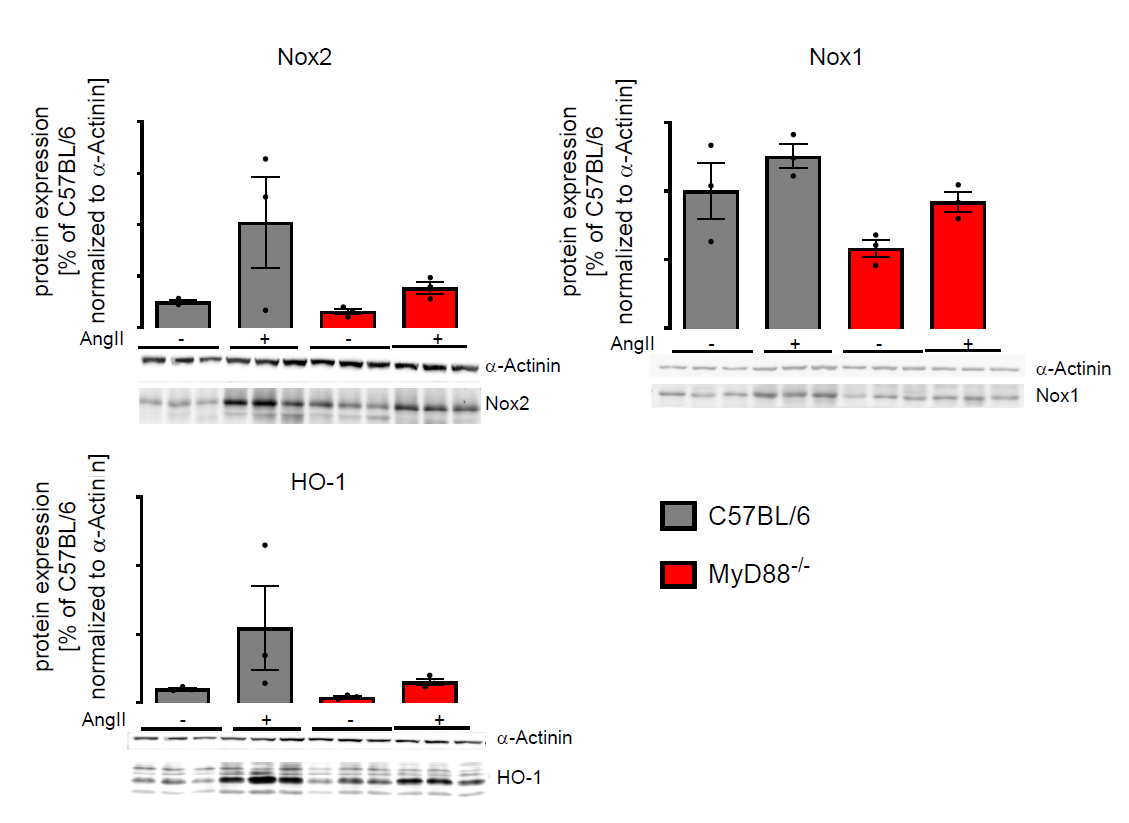


Supplementary Figure 2: Angiotensin II-induced increase in protein expression of oxidative stress marker proteins is ameliorated in MyD88 knockout mice. Aortic expression of phagocytic (Nox2) and vascular (Nox1) NADPH oxidase isoforms and Heme oxygenase-1 (HO-1) was assessed by western blot analysis and specific antibodies. Representative blots for all proteins are shown below the densitometric quantification. The data are displayed as percentage of C57BL/6 controls of 3 animals per group. Kruskal Wallis test. Data are mean±SEM. *, P<0.05.


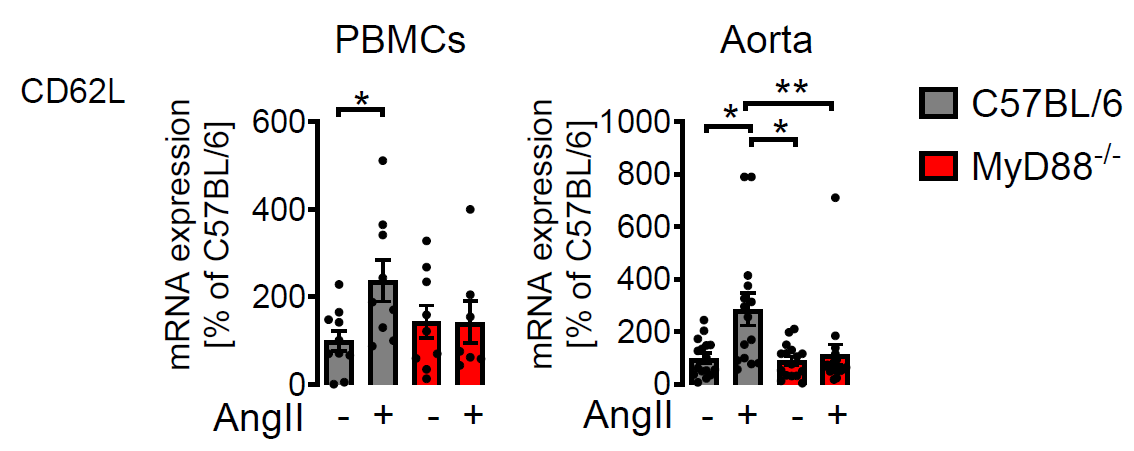


Supplementary Figure 3: MyD88 determines the phenotype of myelomonocytic cells in angiotensin II driven vascular inflammation. Quantification of mRNA levels by qRT-PCR of CD62L (=L-Selectin, inflammatory monocyte marker) in aortic lysates and peripheral blood mononuclear cells (PBMCs). Data are mean±SEM, shown as % of C57BL/6 control animals; Kruskal Wallis test; **, P<0.01; *, P<0.05.


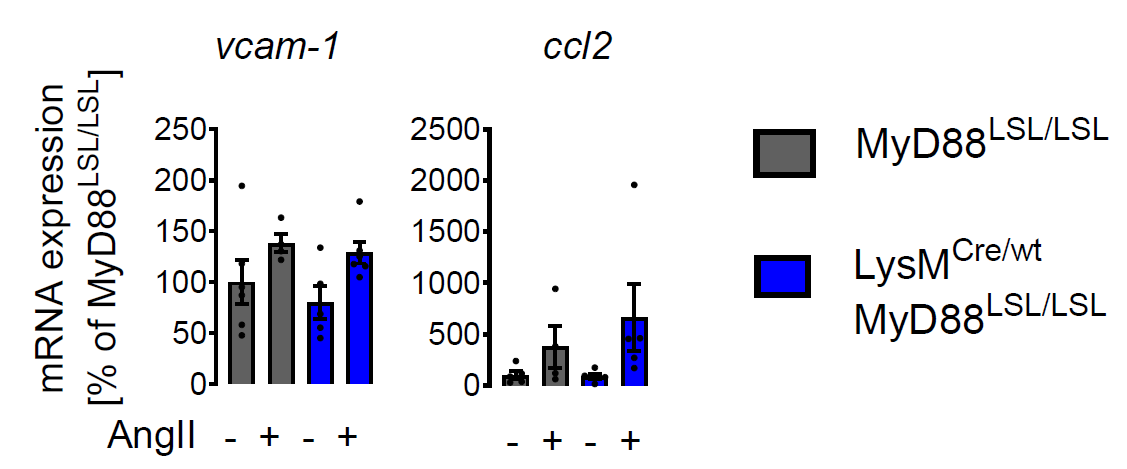


Supplementary Figure 4: MyD88 expression in LysM^+^ cell drives vascular inflammation. Aortic mRNA expression levels of vascular cell adhesion protein-1 (*vcam-*1) and monocyte chemoattractant potein-1 (*ccl-2*) were assessed by qRT-PCR. Data are displayed as percentage of MyD88^LSL/LSL^ controls; Kruskal Wallis test. Data are mean±SEM.


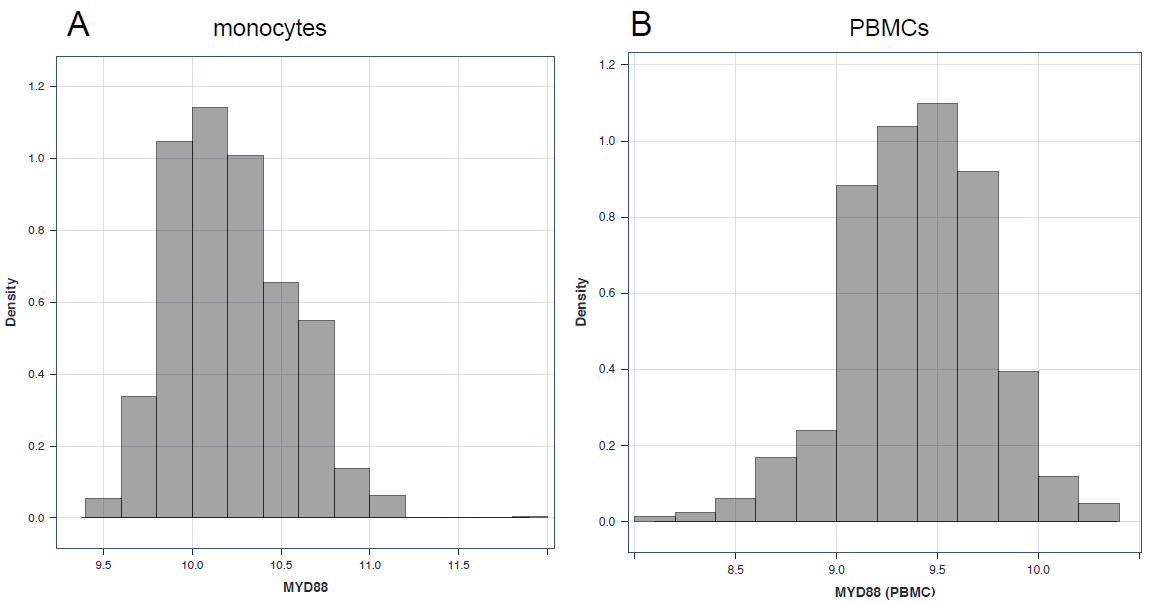


Supplementary Figure 5: Distribution of MyD88 mRNA expression in the GHS. Distribution of expression of MyD88 mRNA isolated from circulating monocytes (A, 1,274 individuals: 646 non-hypertensive, 628 hypertensive) and peripheral blood mononuclear cells (PBMCs, B, 419 individuals: 232 non-hypertensive, 187 hypertensive) of the GHS was analyzed by array and by qRT-PCR.


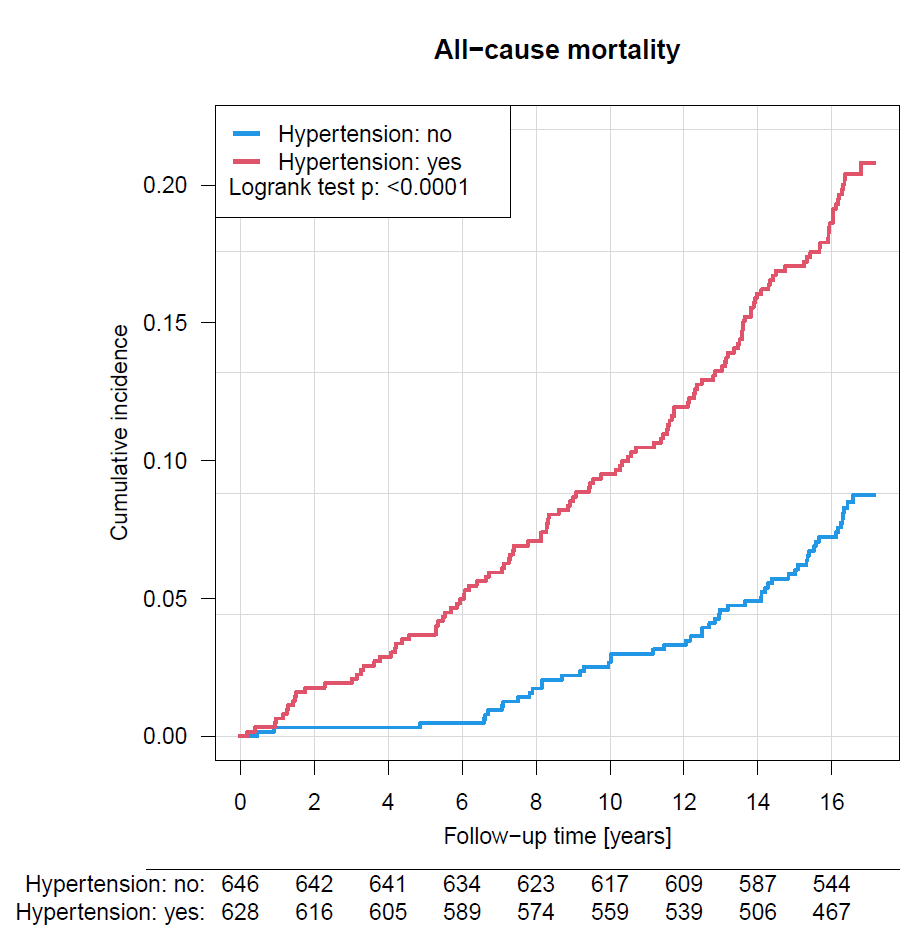


Supplementary Figure 6: All-cause mortality in individuals with arterial hypertension. Kaplan–Meier curves of cumulative all-cause mortality of hypertensive individuals from the Gutenberg Health Study. Median follow-up was 16.5 years. Subjects were tested for differences in cumulative all-cause mortality by log-rank test.


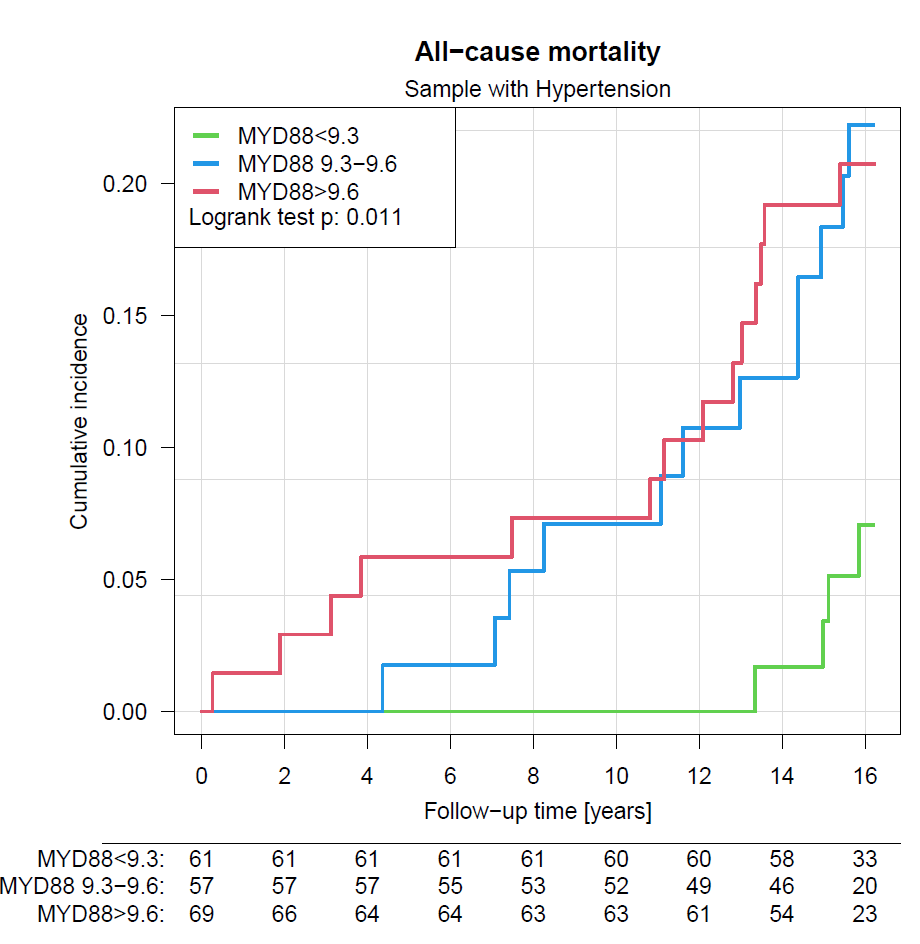


Supplementary Figure 7: MyD88 mRNA expression in PBMCs and all-cause mortality in individuals with arterial hypertension. Kaplan–Meier curves of cumulative all-cause mortality of hypertensive individuals from the Gutenberg Health Study. Data were sex and age weighted. Individuals were stratified according to tertiles MyD88 mRNA expression levels Median follow-up was 16.5 years; Subjects were tested for differences in cumulative all-cause mortality by log-rank test.


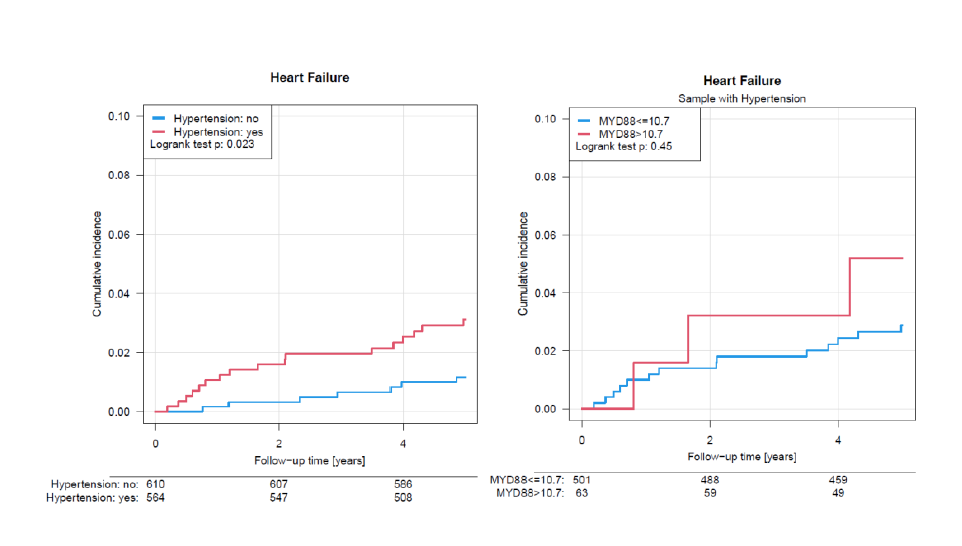


Supplementary Figure 8: Predictive value of Hypertension and monocytic MyD88 expression on the risk of HF. Effect of arterial hypertension and MyD88 mRNA expression in monocytic cells on incident heart failure at 5-year follow-up in 1,273 individuals of the GHS, in which mRNA expression as well as follow-up data were available. Left panel: Cumulative incidence of HF with vs. without prevalent Hypertension at baseline. Left: Cumulative incidence of HF with prevalent Hypertension at baseline, stratified in MyD88 mRNA >10.7 vs. <10.7.

**Supplementary Table 1**

| **myd88_interactor** | **type** | **source** | **pmid** |
| --- | --- | --- | --- |
| TRAF3 | Level 1 | Gene list union |  |
| NFKBIA | Level 1 |  |  |
| IKBKB | Level 1 |  |  |
| TLR9 | Level 1 |  |  |
| TLR7 | Level 1 |  |  |
| MAP3K1 | Level 1 |  |  |
| RAC1 | Level 1 |  |  |
| UBAP1 | Level 1 |  |  |
| SARM1 | Level 1 |  |  |
| BANK1 | Level 1 |  |  |
| SMAD6 | Level 1 |  |  |
| IL1B | Level 1 |  |  |
| TLR2 | Level 1 |  |  |
| TLR10 | Level 1 |  |  |
| IL1R1 | Level 1 |  |  |
| TXN | Level 1 |  |  |
| FADD | Level 1 |  |  |
| IRF5 | Level 1 |  |  |
| SMAD3 | Level 1 |  |  |
| HIVEP3 | Level 2 |  |  |
| PNPT1 | Level 2 |  |  |
| FGD5 | Level 2 |  |  |
| TBC1D1 | Level 2 |  |  |
| CSNK1G3 | Level 2 |  |  |
| ZC3HC1 | Level 2 |  |  |
| PSMD5 | Level 2 |  |  |
| PSMC3 | Level 2 |  |  |
| LRRC10B | Level 2 |  |  |
| SETBP1 | Level 2 |  |  |
| INSR | Level 2 |  |  |
| CRYAA | Level 2 |  |  |
| SIK1 | Level 2 |  |  |
| CASZ1 | Level 2 |  |  |
| MTHFR | Level 2 |  |  |
| CAPZA1 | Level 2 |  |  |
| MOV10 | Level 2 |  |  |
| MDM4 | Level 2 |  |  |
| NCAPH | Level 2 |  |  |
| GRB14 | Level 2 |  |  |
| ATG7 | Level 2 |  |  |
| SLC4A7 | Level 2 |  |  |
| ULK4 | Level 2 |  |  |
| MAP4 | Level 2 |  |  |
| MECOM | Level 2 |  |  |
| GUCY1A1 | Level 2 |  |  |
| GUCY1B1 | Level 2 |  |  |
| NPR3 | Level 2 |  |  |
| HFE | Level 2 |  |  |
| PRRC2A | Level 2 |  |  |
| ABHD16A | Level 2 |  |  |
| ZNF318 | Level 2 |  |  |
| PLEKHG1 | Level 2 |  |  |
| BLK | Level 2 |  |  |
| CACNB2 | Level 2 |  |  |
| PLCE1 | Level 2 |  |  |
| NT5C2 | Level 2 |  |  |
| LSP1 | Level 2 |  |  |
| TNNT3 | Level 2 |  |  |
| PLEKHA7 | Level 2 |  |  |
| SIPA1 | Level 2 |  |  |
| ARHGAP42 | Level 2 |  |  |
| PDE3A | Level 2 |  |  |
| ATP2B1 | Level 2 |  |  |
| TBX3 | Level 2 |  |  |
| ULK3 | Level 2 |  |  |
| FURIN | Level 2 |  |  |
| FES | Level 2 |  |  |
| GOSR2 | Level 2 |  |  |
| ZNF652 | Level 2 |  |  |
| JAG1 | Level 2 |  |  |
| GNAS | Level 2 |  |  |
| L2HGDH | Level 2 |  |  |
| PBX3 | Level 2 |  |  |
| KDM4B | Level 2 |  |  |
| YES1 | Level 2 |  |  |
| LRP4 | Level 2 |  |  |
| TERT | Level 2 |  |  |
| MARK3 | Level 2 |  |  |
| NSD3 | Level 2 |  |  |
| TOP3A | Level 2 |  |  |
| MSRA | Level 2 |  |  |
| FAM193A | Level 2 |  |  |
| INPP5A | Level 2 |  |  |
| WDR7 | Level 2 |  |  |
| CDKAL1 | Level 2 |  |  |
| NFATC2 | Level 2 |  |  |
| MEX3C | Level 2 |  |  |
| ARHGEF25 | Level 2 |  |  |
| TGFBR2 | Level 2 |  |  |
| KLF5 | Level 2 |  |  |
| PDE8A | Level 2 |  |  |
| IGF1 | Level 2 |  |  |
| SYT1 | Level 2 |  |  |
| BCL2 | Level 2 |  |  |
| THADA | Level 2 |  |  |
| XPR1 | Level 2 |  |  |
| ZMAT2 | Level 2 |  |  |
| MEF2A | Level 2 |  |  |
| PTPRD | Level 2 |  |  |
| FOXC1 | Level 2 |  |  |
| PLXNB2 | Level 2 |  |  |
| EPB41L2 | Level 2 |  |  |
| LRCH1 | Level 2 |  |  |
| PPP2R2D | Level 2 |  |  |
| AQP1 | Level 2 |  |  |
| CTNNB1 | Level 2 |  |  |
| ARL14EP | Level 2 |  |  |
| TNKS | Level 2 |  |  |
| CENPP | Level 2 |  |  |
| FARP2 | Level 2 |  |  |
| TSNARE1 | Level 2 |  |  |
| BMPR1B | Level 2 |  |  |
| NIBAN2 | Level 2 |  |  |
| MERTK | Level 2 |  |  |
| RAD52 | Level 2 |  |  |
| WNT4 | Level 2 |  |  |
| CITED2 | Level 2 |  |  |
| KAT2B | Level 2 |  |  |
| POM121C | Level 2 |  |  |
| BCAR3 | Level 2 |  |  |
| HSPA12A | Level 2 |  |  |
| BCAS3 | Level 2 |  |  |
| GABRA2 | Level 2 |  |  |
| ARHGAP29 | Level 2 |  |  |
| TRIP12 | Level 2 |  |  |
| ERBB4 | Level 2 |  |  |
| JAZF1 | Level 2 |  |  |
| IRF6 | Level 2 |  |  |
| DGKH | Level 2 |  |  |
| MLF1 | Level 2 |  |  |
| SPIB | Level 2 |  |  |
| ARIH2 | Level 2 |  |  |
| PRKD1 | Level 2 |  |  |
| SOX5 | Level 2 |  |  |
| RTN4 | Level 2 |  |  |
| DENND2B | Level 2 |  |  |
| SREK1 | Level 2 |  |  |
| TARS1 | Level 2 |  |  |
| RBFOX1 | Level 2 |  |  |
| RBM26 | Level 2 |  |  |
| CAPRIN1 | Level 2 |  |  |
| SKI | Level 2 |  |  |
| CCT6A | Level 2 |  |  |
| ZBTB20 | Level 2 |  |  |
| TASOR2 | Level 2 |  |  |
| GFPT1 | Level 2 |  |  |
| RBM6 | Level 2 |  |  |
| ZNF589 | Level 2 |  |  |
| UHRF1BP1 | Level 2 |  |  |
| NSUN6 | Level 2 |  |  |
| SFXN4 | Level 2 |  |  |
| LYZ | Level 2 |  |  |
| CDC16 | Level 2 |  |  |
| EXOSC6 | Level 2 |  |  |
| CRLF3 | Level 2 |  |  |
| ZNF100 | Level 2 |  |  |
| NFATC1 | Level 2 |  |  |
| ZBTB34 | Level 2 |  |  |
| YWHAQ | Level 2 |  |  |
| GHR | Level 2 |  |  |
| MBTPS1 | Level 2 |  |  |
| NUP93 | Level 2 |  |  |
| TENT5C | Level 2 |  |  |
| GADD45A | Level 2 |  |  |
| NR4A1 | Level 2 |  |  |
| TRPC4 | Level 2 |  |  |
| EIF4G3 | Level 2 |  |  |
| IL20RB | Level 2 |  |  |
| RAP1A | Level 2 |  |  |
| SUPT16H | Level 2 |  |  |
| FAT1 | Level 2 |  |  |
| ZNF227 | Level 2 |  |  |
| TFAP2A | Level 2 |  |  |
| CAP2 | Level 2 |  |  |
| SIPA1L1 | Level 2 |  |  |
| MEX3B | Level 2 |  |  |
| SENP8 | Level 2 |  |  |
| PDE4D | Level 2 |  |  |
| NFIL3 | Level 2 |  |  |
| OXNAD1 | Level 2 |  |  |
| ZNF250 | Level 2 |  |  |
| HIGD1C | Level 2 |  |  |
| UBE3C | Level 2 |  |  |
| ACTN3 | Level 2 |  |  |
| GPAT3 | Level 2 |  |  |
| DAB2 | Level 2 |  |  |
| TLE1 | Level 2 |  |  |
| NFIA | Level 2 |  |  |
| SMAD7 | Level 2 |  |  |
| RNF165 | Level 2 |  |  |
| SPTBN1 | Level 2 |  |  |
| TRIB2 | Level 2 |  |  |
| MIR138-1 | Level 2 |  |  |
| BIRC6 | Level 2 |  |  |
| UGGT1 | Level 2 |  |  |
| TEK | Level 2 |  |  |
| NAV2 | Level 2 |  |  |
| DHX57 | Level 2 |  |  |
| FOXP1 | Level 2 |  |  |
| MTF2 | Level 2 |  |  |
| CIT | Level 2 |  |  |
| BIN1 | Level 2 |  |  |
| NFIB | Level 2 |  |  |
| SH3YL1 | Level 2 |  |  |
| ABL1 | Level 2 |  |  |
| BCL6 | Level 2 |  |  |
| ARHGAP20 | Level 2 |  |  |
| MITF | Level 2 |  |  |
| GPC6 | Level 2 |  |  |
| SCAF11 | Level 2 |  |  |
| TRIM33 | Level 2 |  |  |
| CEP78 | Level 2 |  |  |
| SLC39A10 | Level 2 |  |  |
| RNF213 | Level 2 |  |  |
| FARP1 | Level 2 |  |  |
| ETV6 | Level 2 |  |  |
| TMPRSS6 | Level 2 |  |  |
| RERE | Level 2 |  |  |
| NUCKS1 | Level 2 |  |  |
| ARL14 | Level 2 |  |  |
| COPS4 | Level 2 |  |  |
| HBS1L | Level 2 |  |  |
| UHRF2 | Level 2 |  |  |
| CLNS1A | Level 2 |  |  |
| ALKBH5 | Level 2 |  |  |
| CDC27 | Level 2 |  |  |
| NRIP1 | Level 2 |  |  |
| BMPR2 | Level 2 |  |  |
| AGTR1 | Level 2 |  |  |
| EIF2AK4 | Level 2 |  |  |
| SARS2 | Level 2 |  |  |
| SMAD9 | Level 2 |  |  |
| CPS1 | Level 2 |  |  |
| CAV1 | Level 2 |  |  |
| PKD1 | Level 2 |  |  |
| ATP13A3 | Level 2 |  |  |
| ENG | Level 2 |  |  |
| CACNA1C | Level 2 |  |  |
| ADRB2 | Level 2 |  |  |
| CA2 | Level 2 |  |  |
| SCNN1A | Level 2 |  |  |
| ROCK2 | Level 2 |  |  |
| ROCK1 | Level 2 |  |  |
| NR3C1 | Level 2 |  |  |
| SCN8A | Level 2 |  |  |
| CHRM3 | Level 2 |  |  |
| GABRE | Level 2 |  |  |
| SLC7A1 | Level 2 |  |  |
| NISCH | Level 2 |  |  |
| PGR | Level 2 |  |  |
| NDUFA7 | Level 2 |  |  |
| SWAP70 | Level 2 |  |  |
| NDUFS4 | Level 2 |  |  |
| KDR | Level 2 |  |  |
| CELA2A | Level 2 |  |  |
| NPNT | Level 2 |  |  |
| NDUFAB1 | Level 2 |  |  |
| NDUFB4 | Level 2 |  |  |
| NDUFA6 | Level 2 |  |  |
| NDUFA8 | Level 2 |  |  |
| NDUFA4 | Level 2 |  |  |
| NDUFB10 | Level 2 |  |  |
| NDUFA9 | Level 2 |  |  |
| NDUFS3 | Level 2 |  |  |
| NDUFS6 | Level 2 |  |  |
| NDUFA5 | Level 2 |  |  |
| NDUFA12 | Level 2 |  |  |
| NDUFV3 | Level 2 |  |  |
| NDUFB5 | Level 2 |  |  |
| NDUFS2 | Level 2 |  |  |
| NDUFA13 | Level 2 |  |  |
| NDUFAF4 | Level 2 |  |  |
| NDUFC2 | Level 2 |  |  |
| NDUFS8 | Level 2 |  |  |
| GPD2 | Level 2 |  |  |
| NDUFS7 | Level 2 |  |  |
| NDUFV2 | Level 2 |  |  |
| NDUFA2 | Level 2 |  |  |
| NDUFB3 | Level 2 |  |  |
| NDUFB8 | Level 2 |  |  |
| NDUFS5 | Level 2 |  |  |
| NDUFB6 | Level 2 |  |  |
| NDUFS1 | Level 2 |  |  |
| NDUFB11 | Level 2 |  |  |
| NDUFA10 | Level 2 |  |  |
| NDUFV1 | Level 2 |  |  |
| CHRNB4 | Level 2 |  |  |
| VDR | Level 2 |  |  |
| PCDH18 | Level 2 |  |  |
| SVEP1 | Level 2 |  |  |
| PPARA | Level 2 |  |  |
| CMIP | Level 2 |  |  |
| COL4A1 | Level 2 |  |  |
| SH3PXD2A | Level 2 |  |  |
| ASXL1 | Level 2 |  |  |
| KIT | Level 2 |  |  |
| RUNX3 | Level 2 |  |  |
| DARS2 | Level 2 |  |  |
| PDGFRB | Level 2 |  |  |
| DNMT3A | Level 2 |  |  |
| PIK3CG | Level 2 |  |  |
| NOS3 | Level 2 |  |  |
| NGF | Level 2 |  |  |
| ZBTB10 | Level 2 |  |  |
| ADORA2A | Level 2 |  |  |
| FBN2 | Level 2 |  |  |
| CCDC170 | Level 2 |  |  |
| PLCH1 | Level 2 |  |  |
| ERAP1 | Level 2 |  |  |
| BCR | Level 2 |  |  |
| ABCC8 | Level 2 |  |  |
| FRMD3 | Level 2 |  |  |
| TNF | Level 2 |  |  |
| DPP4 | Level 2 |  |  |
| COL4A2 | Level 2 |  |  |
| ESR1 | Level 2 |  |  |
| PDE10A | Level 2 |  |  |
| PURG | Level 2 |  |  |
| ZNF423 | Level 2 |  |  |
| PPARG | Level 2 |  |  |
| IRAK1BP1 | Level 2 |  |  |
| PKD2 | Level 2 |  |  |
| SOS2 | Level 2 |  |  |
| SEPTIN9 | Level 2 |  |  |
| COL4A4 | Level 2 |  |  |
| PHIP | Level 2 |  |  |
| WT1 | Level 2 |  |  |
| TH | Level 2 |  |  |
| PKHD1 | Level 2 |  |  |
| MAOA | Level 2 |  |  |
| HIPK2 | Level 2 |  |  |
| TTC21B | Level 2 |  |  |
| CHDH | Level 2 |  |  |
| CDKN2B | Level 2 |  |  |
| HDAC7 | Level 2 |  |  |
| TENM3 | Level 2 |  |  |
| EBF2 | Level 2 |  |  |
| MEF2C | Level 2 |  |  |
| MTX2 | Level 2 |  |  |
| MME | Level 2 |  |  |
| COL4A5 | Level 2 |  |  |
| PLG | Level 2 |  |  |
| PDGFRA | Level 2 |  |  |
| CSF1R | Level 2 |  |  |
| GRK6 | Level 2 |  |  |
| CCND1 | Level 2 |  |  |
| F12 | Level 2 |  |  |
| ANO7 | Level 2 |  |  |
| SLC25A37 | Level 2 |  |  |
| PITX2 | Level 2 |  |  |
| MEN1 | Level 2 |  |  |
| DIPK1A | Level 2 |  |  |
| RPL5 | Level 2 |  |  |
| PMS2 | Level 2 |  |  |
| PPP2R2B | Level 2 |  |  |
| PKP4 | Level 2 |  |  |
| UFL1 | Level 2 |  |  |
| HIVEP2 | Level 2 |  |  |
| ADH1B | Level 2 |  |  |
| NAA16 | Level 2 |  |  |
| PREX1 | Level 2 |  |  |
| NOP58 | Level 2 |  |  |
| MFHAS1 | Level 2 |  |  |
| CACNA1H | Level 2 |  |  |
| PTGER3 | Level 2 |  |  |
| NCOR2 | Level 2 |  |  |
| ADGRG6 | Level 2 |  |  |
| TBC1D19 | Level 2 |  |  |
| MAPK4 | Level 2 |  |  |
| MYO9B | Level 2 |  |  |
| PLCB1 | Level 2 |  |  |
| PRKAG2 | Level 2 |  |  |
| HOXC4 | Level 2 |  |  |
| CCND2 | Level 2 |  |  |
| RAD51B | Level 2 |  |  |
| CNNM2 | Level 2 |  |  |
| KLHL21 | Level 2 |  |  |
| SOX6 | Level 2 |  |  |
| HSD17B12 | Level 2 |  |  |
| MYO6 | Level 2 |  |  |
| EFEMP1 | Level 2 |  |  |
| PAQR5 | Level 2 |  |  |
| HUWE1 | Level 2 |  |  |
| NFE2L2 | Level 2 |  |  |
| PTPRJ | Level 2 |  |  |
| PRDM1 | Level 2 |  |  |
| PALM2AKAP2 | Level 2 |  |  |
| SBF2 | Level 2 |  |  |
| ANKRD55 | Level 2 |  |  |
| ITGB5 | Level 2 |  |  |
| CXADR | Level 2 |  |  |
| ADCY9 | Level 2 |  |  |
| SHROOM3 | Level 2 |  |  |
| CA12 | Level 2 |  |  |
| GTF2I | Level 2 |  |  |
| CDKN2A | Level 2 |  |  |
| CDC25A | Level 2 |  |  |
| KEAP1 | Level 2 |  |  |
| MOCS2 | Level 2 |  |  |
| HSPA4 | Level 2 |  |  |
| TUBB1 | Level 2 |  |  |
| HAUS8 | Level 2 |  |  |
| GRIN1 | Level 2 |  |  |
| STXBP5 | Level 2 |  |  |
| SNX31 | Level 2 |  |  |
| ESR2 | Level 2 |  |  |
| VIM | Level 2 |  |  |
| FAM13A | Level 2 |  |  |
| ARL15 | Level 2 |  |  |
| MYH9 | Level 2 |  |  |
| TUBB6 | Level 2 |  |  |
| GRIN2A | Level 2 |  |  |
| TUBB3 | Level 2 |  |  |
| TUBB4A | Level 2 |  |  |
| TUBB2A | Level 2 |  |  |
| TUBB4B | Level 2 |  |  |
| TUBB8 | Level 2 |  |  |
| TUBB | Level 2 |  |  |
| GRIN3B | Level 2 |  |  |
| GRIN2D | Level 2 |  |  |
| TUBB2B | Level 2 |  |  |
| ARHGAP15 | Level 2 |  |  |
| LMO1 | Level 2 |  |  |
| USP28 | Level 2 |  |  |
| ARHGEF18 | Level 2 |  |  |
| LEF1 | Level 2 |  |  |
| FLT1 | Level 2 |  |  |
| GRIA2 | Level 2 |  |  |
| HSF2BP | Level 2 |  |  |
| ZBTB46 | Level 2 |  |  |
| GJB2 | Level 2 |  |  |
| INF2 | Level 2 |  |  |
| ZFP36L1 | Level 2 |  |  |
| F10 | Level 2 |  |  |
| CFDP1 | Level 2 |  |  |
| BCL2L11 | Level 2 |  |  |
| MGAM | Level 2 |  |  |
| GRIA1 | Level 2 |  |  |
| GRIK1 | Level 2 |  |  |
| GRIA3 | Level 2 |  |  |
| GRIK2 | Level 2 |  |  |
| GRIK5 | Level 2 |  |  |
| WNT3A | Level 2 |  |  |
| KDM2B | Level 2 |  |  |
| NIFK | Level 2 |  |  |
| MKLN1 | Level 2 |  |  |
| NHLRC2 | Level 2 |  |  |
| ID2 | Level 2 |  |  |
| USP8 | Level 2 |  |  |
| FOSL2 | Level 2 |  |  |
| DNAJC10 | Level 2 |  |  |
| YWHAE | Level 2 |  |  |
| ZSWIM6 | Level 2 |  |  |
| TET2 | Level 2 |  |  |
| RALGPS1 | Level 2 |  |  |
| TMEFF2 | Level 2 |  |  |
| PGPEP1 | Level 2 |  |  |
| GTF2B | Level 2 |  |  |
| PIK3R1 | Level 2 |  |  |
| DCAKD | Level 2 |  |  |
| SOX3 | Level 2 |  |  |
| ZNF268 | Level 2 |  |  |
| NFAT5 | Level 2 |  |  |
| GPATCH2 | Level 2 |  |  |
| KLHL24 | Level 2 |  |  |
| TRPC5 | Level 2 |  |  |
| CREB1 | Level 2 |  |  |
| PPP2R2A | Level 2 |  |  |
| NR2F2 | Level 2 |  |  |
| MAD1L1 | Level 2 |  |  |
| SLC2A4 | Level 2 |  |  |
| LRRC7 | Level 2 |  |  |
| DCP2 | Level 2 |  |  |
| CWF19L2 | Level 2 |  |  |
| PABPC4 | Level 2 |  |  |
| FBF1 | Level 2 |  |  |
| TCEA2 | Level 2 |  |  |
| DOCK3 | Level 2 |  |  |
| ZNF443 | Level 2 |  |  |
| MON2 | Level 2 |  |  |
| FBRSL1 | Level 2 |  |  |
| MEF2D | Level 2 |  |  |
| PRKD3 | Level 2 |  |  |
| SPRY4 | Level 2 |  |  |
| COL4A3 | Level 2 |  |  |
| GLI2 | Level 2 |  |  |
| IPO9 | Level 2 |  |  |
| MORC3 | Level 2 |  |  |
| STN1 | Level 2 |  |  |
| DLG4 | Level 2 |  |  |
| DNAJC1 | Level 2 |  |  |
| KIF23 | Level 2 |  |  |
| RNF126 | Level 2 |  |  |
| ANKS1A | Level 2 |  |  |
| EHD4 | Level 2 |  |  |
| SPI1 | Level 2 |  |  |
| TNRC6A | Level 2 |  |  |
| PEPD | Level 2 |  |  |
| PRDM16 | Level 2 |  |  |
| S1PR2 | Level 2 |  |  |
| EMSY | Level 2 |  |  |
| CSK | Level 2 |  |  |
| MCU | Level 2 |  |  |
| TSN | Level 2 |  |  |
| MS4A4A | Level 2 |  |  |
| TPPP | Level 2 |  |  |
| GGCX | Level 2 |  |  |
| NUMB | Level 2 |  |  |
| USP34 | Level 2 |  |  |
| CCDC88C | Level 2 |  |  |
| VEGFA | Level 2 |  |  |
| MTUS2 | Level 2 |  |  |
| RRP1B | Level 2 |  |  |
| ZNF407 | Level 2 |  |  |
| ERI1 | Level 2 |  |  |
| HMGA1 | Level 2 |  |  |
| NOTCH3 | Level 2 |  |  |
| CHMP1A | Level 2 |  |  |
| CEP170 | Level 2 |  |  |
| MADD | Level 2 |  |  |
| DPYSL2 | Level 2 |  |  |
| DNMT1 | Level 2 |  |  |
| IMMP2L | Level 2 |  |  |
| SLC1A3 | Level 2 |  |  |
| HOXA10 | Level 2 |  |  |
| IGF2BP2 | Level 2 |  |  |
| FGFR2 | Level 2 |  |  |
| ARID3C | Level 2 |  |  |
| H2BC4 | Level 2 |  |  |
| CENPV | Level 2 |  |  |
| MAPKAP1 | Level 2 |  |  |
| TP53INP1 | Level 2 |  |  |
| SDCCAG8 | Level 2 |  |  |
| CLINT1 | Level 2 |  |  |
| TRIM65 | Level 2 |  |  |
| MARCHF2 | Level 2 |  |  |
| EEFSEC | Level 2 |  |  |
| HOXB7 | Level 2 |  |  |
| NOTCH1 | Level 2 |  |  |
| PIP4K2B | Level 2 |  |  |
| EDA2R | Level 2 |  |  |
| PLCG1 | Level 2 |  |  |
| MYNN | Level 2 |  |  |
| AKR1B1 | Level 2 |  |  |
| NMT1 | Level 2 |  |  |
| RIN3 | Level 2 |  |  |
| ATXN2 | Level 2 |  |  |
| SMAD4 | Level 2 |  |  |
| ZZEF1 | Level 2 |  |  |
| CLDN7 | Level 2 |  |  |
| PKN2 | Level 2 |  |  |
| PIK3C2B | Level 2 |  |  |
| ZHX3 | Level 2 |  |  |
| PNO1 | Level 2 |  |  |
| ZDHHC18 | Level 2 |  |  |
| NUF2 | Level 2 |  |  |
| CCDC90B | Level 2 |  |  |
| FBXO38 | Level 2 |  |  |
| NEK10 | Level 2 |  |  |
| GEMIN2 | Level 2 |  |  |
| SEC23A | Level 2 |  |  |
| GMDS | Level 2 |  |  |
| CHP1 | Level 2 |  |  |
| TRAF3IP3 | Level 2 |  |  |
| METTL15 | Level 2 |  |  |
| SIK2 | Level 2 |  |  |
| TASOR | Level 2 |  |  |
| AKR1B15 | Level 2 |  |  |
| CLCNKA | Level 2 |  |  |
| SHISA4 | Level 2 |  |  |
| THAP3 | Level 2 |  |  |
| NOC3L | Level 2 |  |  |
| VARS1 | Level 2 |  |  |
| GFM1 | Level 2 |  |  |
| ZFP91 | Level 2 |  |  |
| RPL4 | Level 2 |  |  |
| ARHGEF16 | Level 2 |  |  |
| ILRUN | Level 2 |  |  |
| PRKAA2 | Level 2 |  |  |
| PODXL | Level 2 |  |  |
| BNIP3L | Level 2 |  |  |
| TRIM47 | Level 2 |  |  |
| WRN | Level 2 |  |  |
| BMPR1A | Level 2 |  |  |
| HIBADH | Level 2 |  |  |
| RHOC | Level 2 |  |  |
| SIDT2 | Level 2 |  |  |
| CYTH1 | Level 2 |  |  |
| MRPS12 | Level 2 |  |  |
| KSR2 | Level 2 |  |  |
| PHF6 | Level 2 |  |  |
| RBM23 | Level 2 |  |  |
| DCP1A | Level 2 |  |  |
| SMG6 | Level 2 |  |  |
| OPRL1 | Level 2 |  |  |
| CDKN1B | Level 2 |  |  |
| CEBPZ | Level 2 |  |  |
| ICA1L | Level 2 |  |  |
| SNAPC5 | Level 2 |  |  |
| MCC | Level 2 |  |  |
| SMAD1 | Level 2 |  |  |
| SPRYD7 | Level 2 |  |  |
| SGK1 | Level 2 |  |  |
| WDR11 | Level 2 |  |  |
| EPB41 | Level 2 |  |  |
| PAFAH1B2 | Level 2 |  |  |
| GFER | Level 2 |  |  |
| MAST2 | Level 2 |  |  |
| ALG9 | Level 2 |  |  |
| INO80 | Level 2 |  |  |
| FOLH1 | Level 2 |  |  |
| IL17RB | Level 2 |  |  |
| CARF | Level 2 |  |  |
| PPP3R1 | Level 2 |  |  |
| VKORC1 | Level 2 |  |  |
| AOC1 | Level 2 |  |  |
| CISD2 | Level 2 |  |  |
| CKB | Level 2 |  |  |
| SRRT | Level 2 |  |  |
| IL17A | Level 2 |  |  |
| FKBP1A | Level 2 |  |  |
| HEXIM1 | Level 2 |  |  |
| ARID5B | Level 2 |  |  |
| HOXB5 | Level 2 |  |  |
| IL6R | Level 2 |  |  |
| SLX4IP | Level 2 |  |  |
| ARF1 | Level 2 |  |  |
| PIM3 | Level 2 |  |  |
| H4C3 | Level 2 |  |  |
| NR2C2 | Level 2 |  |  |
| CDK6 | Level 2 |  |  |
| PTEN | Level 2 |  |  |
| MTNR1A | Level 2 |  |  |
| OPRM1 | Level 2 |  |  |
| SENP2 | Level 2 |  |  |
| MPO | Level 2 |  |  |
| MKRN2 | Level 2 |  |  |
| ZNF875 | Level 2 |  |  |
| KCNRG | Level 2 |  |  |
| ZAP70 | Level 2 |  |  |
| ZNF738 | Level 2 |  |  |
| RNF34 | Level 2 |  |  |
| MC4R | Level 2 |  |  |
| ERAP2 | Level 2 |  |  |
| BRD2 | Level 2 |  |  |
| PHETA1 | Level 2 |  |  |
| SUN1 | Level 2 |  |  |
| UBE2D3 | Level 2 |  |  |
| LXN | Level 2 |  |  |
| GET4 | Level 2 |  |  |
| CTDNEP1 | Level 2 |  |  |
| LOX | Level 2 |  |  |
| PCM1 | Level 2 |  |  |
| SNRPC | Level 2 |  |  |
| RPAP3 | Level 2 |  |  |
| EXD3 | Level 2 |  |  |
| PINX1 | Level 2 |  |  |
| ZNF646 | Level 2 |  |  |
| RPS16 | Level 2 |  |  |
| CYB5D1 | Level 2 |  |  |
| BRD4 | Level 2 |  |  |
| CHD3 | Level 2 |  |  |
| PTK2 | Level 2 |  |  |
| AXIN1 | Level 2 |  |  |
| HNRNPM | Level 2 |  |  |
| HLA-DPB1 | Level 2 |  |  |
| XBP1 | Level 2 |  |  |
| KLF3 | Level 2 |  |  |
| ACP2 | Level 2 |  |  |
| PGM2 | Level 2 |  |  |
| GATA2 | Level 2 |  |  |
| NAV1 | Level 2 |  |  |
| PPL | Level 2 |  |  |
| UTP25 | Level 2 |  |  |
| GRHL2 | Level 2 |  |  |
| DCAF16 | Level 2 |  |  |
| MCRS1 | Level 2 |  |  |
| TRIP6 | Level 2 |  |  |
| BRSK2 | Level 2 |  |  |
| HSPH1 | Level 2 |  |  |
| ADAR | Level 2 |  |  |
| TOP1 | Level 2 |  |  |
| XPO1 | Level 2 |  |  |
| GAB1 | Level 2 |  |  |
| ZWILCH | Level 2 |  |  |
| ANAPC5 | Level 2 |  |  |
| POC1B | Level 2 |  |  |
| KDM6B | Level 2 |  |  |
| IL6ST | Level 2 |  |  |
| ZNF708 | Level 2 |  |  |
| AHDC1 | Level 2 |  |  |
| DNM1L | Level 2 |  |  |
| ABCF3 | Level 2 |  |  |
| H4C8 | Level 2 |  |  |
| DVL2 | Level 2 |  |  |
| MRPL38 | Level 2 |  |  |
| FAF1 | Level 2 |  |  |
| MAP3K5 | Level 2 |  |  |
| BRD3 | Level 2 |  |  |
| MUC13 | Level 2 |  |  |
| BRDT | Level 2 |  |  |
| ZNF689 | Level 2 |  |  |
| ALG3 | Level 2 |  |  |
| MFF | Level 2 |  |  |
| GIT2 | Level 2 |  |  |
| CD80 | Level 2 |  |  |
| ARID1A | Level 2 |  |  |
| GEM | Level 2 |  |  |
| EFR3B | Level 2 |  |  |
| TRPA1 | Level 2 |  |  |
| NEDD9 | Level 2 |  |  |
| IGF2BP1 | Level 2 |  |  |
| ARPC3 | Level 2 |  |  |
| HLA-B | Level 2 |  |  |
| RBMXL1 | Level 2 |  |  |
| MAP2K1 | Level 2 |  |  |
| HOXB9 | Level 2 |  |  |
| DDB2 | Level 2 |  |  |
| U2AF1 | Level 2 |  |  |
| RAD51C | Level 2 |  |  |
| BCKDK | Level 2 |  |  |
| SNCAIP | Level 2 |  |  |
| ZFP64 | Level 2 |  |  |
| ACTR1B | Level 2 |  |  |
| MFN2 | Level 2 |  |  |
| VGLL2 | Level 2 |  |  |
| SRSF4 | Level 2 |  |  |
| SLC22A3 | Level 2 |  |  |
| FAM171B | Level 2 |  |  |
| RNF123 | Level 2 |  |  |
| ZNF140 | Level 2 |  |  |
| TMEM199 | Level 2 |  |  |
| SEC31A | Level 2 |  |  |
| IGF2R | Level 2 |  |  |
| WASF2 | Level 2 |  |  |
| SEPTIN4 | Level 2 |  |  |
| PPP2R1B | Level 2 |  |  |
| UBE2Z | Level 2 |  |  |
| ZNF84 | Level 2 |  |  |
| PLCB4 | Level 2 |  |  |
| DDX47 | Level 2 |  |  |
| IRS1 | Level 2 |  |  |
| COG2 | Level 2 |  |  |
| KCTD1 | Level 2 |  |  |
| PRMT5 | Level 2 |  |  |
| H1-6 | Level 2 |  |  |
| NCF1 | Level 2 |  |  |
| OAS2 | Level 2 |  |  |
| PLCB3 | Level 2 |  |  |
| HEXIM2 | Level 2 |  |  |
| TCHP | Level 2 |  |  |
| ACAP1 | Level 2 |  |  |
| CCNT2 | Level 2 |  |  |
| TMEM165 | Level 2 |  |  |
| SIGMAR1 | Level 2 |  |  |
| EIF4G1 | Level 2 |  |  |
| SNF8 | Level 2 |  |  |
| ECHDC1 | Level 2 |  |  |
| HNF4A | Level 2 |  |  |
| C7ORF50 | Level 2 |  |  |
| SLC22A4 | Level 2 |  |  |
| INTS12 | Level 2 |  |  |
| H2AC6 | Level 2 |  |  |
| LARP4 | Level 2 |  |  |
| HDLBP | Level 2 |  |  |
| OBSCN | Level 2 |  |  |
| DCAF1 | Level 2 |  |  |
| BAG4 | Level 2 |  |  |
| TRIM13 | Level 2 |  |  |
| WDR12 | Level 2 |  |  |
| ZNF107 | Level 2 |  |  |
| TBR1 | Level 2 |  |  |
| KLF2 | Level 2 |  |  |
| NUDT16L1 | Level 2 |  |  |
| MCM8 | Level 2 |  |  |
| PIK3R3 | Level 2 |  |  |
| PLEKHA1 | Level 2 |  |  |
| HOXA9 | Level 2 |  |  |
| LANCL1 | Level 2 |  |  |
| SIK3 | Level 2 |  |  |
| PDE4A | Level 2 |  |  |
| ALDH2 | Level 2 |  |  |
| RMDN3 | Level 2 |  |  |
| GPANK1 | Level 2 |  |  |
| KAT8 | Level 2 |  |  |
| PNMA2 | Level 2 |  |  |
| PRKCSH | Level 2 |  |  |
| ZFP36L2 | Level 2 |  |  |
| FDXACB1 | Level 2 |  |  |
| CAST | Level 2 |  |  |
| NQO1 | Level 2 |  |  |
| PPP2R3A | Level 2 |  |  |
| TRIM73 | Level 2 |  |  |
| TSHZ2 | Level 2 |  |  |
| PDE3B | Level 2 |  |  |
| GOLIM4 | Level 2 |  |  |
| FANCA | Level 2 |  |  |
| FST | Level 2 |  |  |
| ATE1 | Level 2 |  |  |
| RRM2B | Level 2 |  |  |
| BCL7A | Level 2 |  |  |
| LPIN3 | Level 2 |  |  |
| DNAAF5 | Level 2 |  |  |
| PLN | Level 2 |  |  |
| NUDC | Level 2 |  |  |
| NUCB2 | Level 2 |  |  |
| PPP1R7 | Level 2 |  |  |
| PRELID1 | Level 2 |  |  |
| RNF146 | Level 2 |  |  |
| PARP1 | Level 2 |  |  |
| PDK1 | Level 2 |  |  |
| STAG1 | Level 2 |  |  |
| SRCIN1 | Level 2 |  |  |
| STX4 | Level 2 |  |  |
| LMNB2 | Level 2 |  |  |
| IL37 | Level 2 |  |  |
| DNAJB8 | Level 2 |  |  |
| TNIP2 | Level 2 |  |  |
| RET | Level 2 |  |  |
| ANKRD13A | Level 2 |  |  |
| POLDIP2 | Level 2 |  |  |
| DNAJC11 | Level 2 |  |  |
| PSMB3 | Level 2 |  |  |
| MICU2 | Level 2 |  |  |
| MTAP | Level 2 |  |  |
| EIF4B | Level 2 |  |  |
| ARHGAP10 | Level 2 |  |  |
| MTMR4 | Level 2 |  |  |
| CNR2 | Level 2 |  |  |
| ZNF527 | Level 2 |  |  |
| NLRC3 | Level 2 |  |  |
| KCNN2 | Level 2 |  |  |
| THG1L | Level 2 |  |  |
| MRPL15 | Level 2 |  |  |
| MAPK11 | Level 2 |  |  |
| MINDY4 | Level 2 |  |  |
| RBM33 | Level 2 |  |  |
| FGFR1 | Level 2 |  |  |
| ZCCHC10 | Level 2 |  |  |
| IRF1 | Level 2 |  |  |
| ADCY3 | Level 2 |  |  |
| ADAMTS12 | Level 2 |  |  |
| FLT4 | Level 2 |  |  |
| SRD5A3 | Level 2 |  |  |
| CTSD | Level 2 |  |  |
| HAUS4 | Level 2 |  |  |
| PPP4R3A | Level 2 |  |  |
| SERTAD4 | Level 2 |  |  |
| MAPK3 | Level 2 |  |  |
| ITGA2 | Level 2 |  |  |
| MTRF1 | Level 2 |  |  |
| PDCD10 | Level 2 |  |  |
| ZNF793 | Level 2 |  |  |
| BAG6 | Level 2 |  |  |
| TKFC | Level 2 |  |  |
| HLA-C | Level 2 |  |  |
| C11ORF58 | Level 2 |  |  |
| RAB24 | Level 2 |  |  |
| RHEB | Level 2 |  |  |
| ALKBH3 | Level 2 |  |  |
| LAMB2 | Level 2 |  |  |
| RASA3 | Level 2 |  |  |
| PTP4A3 | Level 2 |  |  |
| RPP25L | Level 2 |  |  |
| HIP1 | Level 2 |  |  |
| WEE1 | Level 2 |  |  |
| PARP2 | Level 2 |  |  |
| TMEM97 | Level 2 |  |  |
| SLC25A4 | Level 2 |  |  |
| BTN2A1 | Level 2 |  |  |
| PRNP | Level 2 |  |  |
| BRAF | Level 2 |  |  |
| PDXK | Level 2 |  |  |
| KYAT3 | Level 2 |  |  |
| PPP1R12A | Level 2 |  |  |
| HOXA7 | Level 2 |  |  |
| UHRF1 | Level 2 |  |  |
| ZW10 | Level 2 |  |  |
| LMBR1 | Level 2 |  |  |
| KDF1 | Level 2 |  |  |
| DPYS | Level 2 |  |  |
| RAF1 | Level 2 |  |  |
| LIN7B | Level 2 |  |  |
| TCF25 | Level 2 |  |  |
| MTHFD1L | Level 2 |  |  |
| TKT | Level 2 |  |  |
| PDE2A | Level 2 |  |  |
| MOB2 | Level 2 |  |  |
| SOAT2 | Level 2 |  |  |
| ATP1A2 | Level 2 |  |  |
| ATP1A1 | Level 2 |  |  |
| LPXN | Level 2 |  |  |
| RCC1L | Level 2 |  |  |
| SLC29A1 | Level 2 |  |  |
| APEH | Level 2 |  |  |
| FLT3 | Level 2 |  |  |
| PDK2 | Level 2 |  |  |
| CRYBA2 | Level 2 |  |  |
| IMPDH2 | Level 2 |  |  |
| PDE6H | Level 2 |  |  |
| ATP1A3 | Level 2 |  |  |
| IMPDH1 | Level 2 |  |  |
| PDK3 | Level 2 |  |  |
| PDE9A | Level 2 |  |  |
| ATP1A4 | Level 2 |  |  |
| VASN | Level 2 |  |  |
| LMAN2 | Level 2 |  |  |
| CFL1 | Level 2 |  |  |
| SMARCD1 | Level 2 |  |  |
| CLIC1 | Level 2 |  |  |
| IRF3 | Level 2 |  |  |
| PHF8 | Level 2 |  |  |
| ABCB8 | Level 2 |  |  |
| GLYR1 | Level 2 |  |  |
| RGCC | Level 2 |  |  |
| KIF2A | Level 2 |  |  |
| DDAH2 | Level 2 |  |  |
| ZNF76 | Level 2 |  |  |
| SEPTIN2 | Level 2 |  |  |
| ADA | Level 2 |  |  |
| MAP1LC3B | Level 2 |  |  |
| MSH5 | Level 2 |  |  |
| CCNB1 | Level 2 |  |  |
| INA | Level 2 |  |  |
| APEX2 | Level 2 |  |  |
| MAPK10 | Level 2 |  |  |
| NCAPG | Level 2 |  |  |
| PPM1H | Level 2 |  |  |
| LIMA1 | Level 2 |  |  |
| MTA3 | Level 2 |  |  |
| COA8 | Level 2 |  |  |
| CRYAB | Level 2 |  |  |
| CBS | Level 2 |  |  |
| RNF40 | Level 2 |  |  |
| CCDC186 | Level 2 |  |  |
| HOXB6 | Level 2 |  |  |
| SNRNP70 | Level 2 |  |  |
| TSC2 | Level 2 |  |  |
| PCGF2 | Level 2 |  |  |
| TLR1 | Level 2 |  |  |
| VWA8 | Level 2 |  |  |
| TRIM38 | Level 2 |  |  |
| KLHDC10 | Level 2 |  |  |
| IL31 | Level 2 |  |  |
| BSN | Level 2 |  |  |
| NUDT2 | Level 2 |  |  |
| TNFAIP1 | Level 2 |  |  |
| DNAH8 | Level 2 |  |  |
| TBKBP1 | Level 2 |  |  |
| TNKS1BP1 | Level 2 |  |  |
| P4HA2 | Level 2 |  |  |
| ZBTB43 | Level 2 |  |  |
| CHMP4C | Level 2 |  |  |
| KANK2 | Level 2 |  |  |
| FAM167A | Level 2 |  |  |
| ZNF668 | Level 2 |  |  |
| BCAR1 | Level 2 |  |  |
| CORO7 | Level 2 |  |  |
| TOMM40 | Level 2 |  |  |
| C1ORF35 | Level 2 |  |  |
| CHRD | Level 2 |  |  |
| ITGAV | Level 2 |  |  |
| ERP29 | Level 2 |  |  |
| TEX14 | Level 2 |  |  |
| TBL3 | Level 2 |  |  |
| FKBP2 | Level 2 |  |  |
| TM9SF4 | Level 2 |  |  |
| BAG3 | Level 2 |  |  |
| KLF8 | Level 2 |  |  |
| PLCG2 | Level 2 |  |  |
| SYTL1 | Level 2 |  |  |
| SFN | Level 2 |  |  |
| DVL3 | Level 2 |  |  |
| CNTFR | Level 2 |  |  |
| NSF | Level 2 |  |  |
| SCAMP1 | Level 2 |  |  |
| TGIF1 | Level 2 |  |  |
| TCERG1 | Level 2 |  |  |
| FBXO31 | Level 2 |  |  |
| SERINC3 | Level 2 |  |  |
| KIF3B | Level 2 |  |  |
| TENT4B | Level 2 |  |  |
| PAWR | Level 2 |  |  |
| SCAMP2 | Level 2 |  |  |
| TLR6 | Level 2 |  |  |
| EIF2B5 | Level 2 |  |  |
| H2BC10 | Level 2 |  |  |
| ZNF664 | Level 2 |  |  |
| ITPR2 | Level 2 |  |  |
| NCOR1 | Level 2 |  |  |
| CALCOCO2 | Level 2 |  |  |
| TFAM | Level 2 |  |  |
| RMND1 | Level 2 |  |  |
| SLK | Level 2 |  |  |
| KAT5 | Level 2 |  |  |
| TRAP1 | Level 2 |  |  |
| RUVBL1 | Level 2 |  |  |
| FAM107A | Level 2 |  |  |
| AJUBA | Level 2 |  |  |
| PNN | Level 2 |  |  |
| ANAPC7 | Level 2 |  |  |
| PRMT1 | Level 2 |  |  |
| MRM2 | Level 2 |  |  |
| MIA2 | Level 2 |  |  |
| TOPBP1 | Level 2 |  |  |
| MACF1 | Level 2 |  |  |
| PRKAR1B | Level 2 |  |  |
| HADH | Level 2 |  |  |
| ZNF598 | Level 2 |  |  |
| AIG1 | Level 2 |  |  |
| IGFBP6 | Level 2 |  |  |
| FNBP4 | Level 2 |  |  |
| INO80E | Level 2 |  |  |
| COMMD7 | Level 2 |  |  |
| SCD5 | Level 2 |  |  |
| MAPK12 | Level 2 |  |  |
| DPH1 | Level 2 |  |  |
| LYPLA1 | Level 2 |  |  |
| DIP2B | Level 2 |  |  |
| IP6K1 | Level 2 |  |  |
| SH3BP2 | Level 2 |  |  |
| IPO11 | Level 2 |  |  |
| ACTR8 | Level 2 |  |  |
| TRMT6 | Level 2 |  |  |
| ELOVL1 | Level 2 |  |  |
| TTC19 | Level 2 |  |  |
| QPCT | Level 2 |  |  |
| TAOK2 | Level 2 |  |  |
| SENP1 | Level 2 |  |  |
| NUP160 | Level 2 |  |  |
| MYO1C | Level 2 |  |  |
| NAA25 | Level 2 |  |  |
| CRK | Level 2 |  |  |
| CYP2U1 | Level 2 |  |  |
| EMILIN3 | Level 2 |  |  |
| GAS2 | Level 2 |  |  |
| DIMT1 | Level 2 |  |  |
| LRRC40 | Level 2 |  |  |
| NCOA1 | Level 2 |  |  |
| SMARCA5 | Level 2 |  |  |
| ENOSF1 | Level 2 |  |  |
| ZNF785 | Level 2 |  |  |
| GUK1 | Level 2 |  |  |
| TPD52L2 | Level 2 |  |  |
| BAG5 | Level 2 |  |  |
| ZNF22 | Level 2 |  |  |
| CDSN | Level 2 |  |  |
| PLA2G4B | Level 2 |  |  |
| SSRP1 | Level 2 |  |  |
| SYT7 | Level 2 |  |  |
| PPIC | Level 2 |  |  |
| RRM2 | Level 2 |  |  |
| NETO1 | Level 2 |  |  |
| MTCH2 | Level 2 |  |  |
| MGLL | Level 2 |  |  |
| MCMBP | Level 2 |  |  |
| H1-7 | Level 2 |  |  |
| NTHL1 | Level 2 |  |  |
| RAB3D | Level 2 |  |  |
| H2BC3 | Level 2 |  |  |
| PPFIA1 | Level 2 |  |  |
| SPIN3 | Level 2 |  |  |
| SARS1 | Level 2 |  |  |
| SKIL | Level 2 |  |  |
| ETV5 | Level 2 |  |  |
| A1CF | Level 2 |  |  |
| KCNQ1 | Level 2 |  |  |
| LRP2 | Level 2 |  |  |
| WNT7A | Level 2 |  |  |
| NFKB1 | Level 2 |  |  |
| TSPAN9 | Level 2 |  |  |
| PTPRO | Level 2 |  |  |
| SIPA1L3 | Level 2 |  |  |
| CFH | Level 2 |  |  |
| TAF5L | Level 2 |  |  |
| URB2 | Level 2 |  |  |
| GALNT2 | Level 2 |  |  |
| NOTCH4 | Level 2 |  |  |
| ATP6V1B2 | Level 2 |  |  |
| LZTS1 | Level 2 |  |  |
| TRIM37 | Level 2 |  |  |
| SMG8 | Level 2 |  |  |
| CELSR2 | Level 2 |  |  |
| FAM241A | Level 2 |  |  |
| KLHL3 | Level 2 |  |  |
| CDKN1A | Level 2 |  |  |
| INTS11 | Level 2 |  |  |
| CAMTA1 | Level 2 |  |  |
| SPSB1 | Level 2 |  |  |
| KDM1A | Level 2 |  |  |
| SRRM1 | Level 2 |  |  |
| FGR | Level 2 |  |  |
| PHACTR4 | Level 2 |  |  |
| RCC1 | Level 2 |  |  |
| SF3A3 | Level 2 |  |  |
| FGGY | Level 2 |  |  |
| DNAJB4 | Level 2 |  |  |
| DDAH1 | Level 2 |  |  |
| ODF2L | Level 2 |  |  |
| POGZ | Level 2 |  |  |
| S100A4 | Level 2 |  |  |
| UBAP2L | Level 2 |  |  |
| NME7 | Level 2 |  |  |
| QSOX1 | Level 2 |  |  |
| LAMC1 | Level 2 |  |  |
| PFKFB2 | Level 2 |  |  |
| CD46 | Level 2 |  |  |
| CDC42BPA | Level 2 |  |  |
| MBOAT2 | Level 2 |  |  |
| ASAP2 | Level 2 |  |  |
| APOB | Level 2 |  |  |
| KLHL29 | Level 2 |  |  |
| PRKCE | Level 2 |  |  |
| VPS54 | Level 2 |  |  |
| ZNF638 | Level 2 |  |  |
| EXOC6B | Level 2 |  |  |
| SPR | Level 2 |  |  |
| PTCD3 | Level 2 |  |  |
| FHL2 | Level 2 |  |  |
| PAX8 | Level 2 |  |  |
| ZEB2 | Level 2 |  |  |
| ACVR2A | Level 2 |  |  |
| STAM2 | Level 2 |  |  |
| PRPF40A | Level 2 |  |  |
| ACVR1C | Level 2 |  |  |
| STK39 | Level 2 |  |  |
| MAP3K20 | Level 2 |  |  |
| ZNF385B | Level 2 |  |  |
| SPATS2L | Level 2 |  |  |
| CYP20A1 | Level 2 |  |  |
| ICOS | Level 2 |  |  |
| KLF7 | Level 2 |  |  |
| FN1 | Level 2 |  |  |
| TNS1 | Level 2 |  |  |
| PNKD | Level 2 |  |  |
| CYP27A1 | Level 2 |  |  |
| COPS8 | Level 2 |  |  |
| HDAC4 | Level 2 |  |  |
| UBE2E2 | Level 2 |  |  |
| GOLGA4 | Level 2 |  |  |
| ITGA9 | Level 2 |  |  |
| PRSS50 | Level 2 |  |  |
| CAMKV | Level 2 |  |  |
| CACNA2D2 | Level 2 |  |  |
| SLMAP | Level 2 |  |  |
| ATXN7 | Level 2 |  |  |
| PPP4R2 | Level 2 |  |  |
| CMSS1 | Level 2 |  |  |
| SENP7 | Level 2 |  |  |
| TRMT10C | Level 2 |  |  |
| ZBTB38 | Level 2 |  |  |
| MBNL1 | Level 2 |  |  |
| ARHGEF26 | Level 2 |  |  |
| FNDC3B | Level 2 |  |  |
| TBL1XR1 | Level 2 |  |  |
| YEATS2 | Level 2 |  |  |
| DLG1 | Level 2 |  |  |
| MAEA | Level 2 |  |  |
| WDR1 | Level 2 |  |  |
| PROM1 | Level 2 |  |  |
| ARAP2 | Level 2 |  |  |
| RBM47 | Level 2 |  |  |
| PDLIM5 | Level 2 |  |  |
| TBCK | Level 2 |  |  |
| SYNPO2 | Level 2 |  |  |
| GYPA | Level 2 |  |  |
| ZNF827 | Level 2 |  |  |
| LRBA | Level 2 |  |  |
| PALLD | Level 2 |  |  |
| ITGA1 | Level 2 |  |  |
| ACTBL2 | Level 2 |  |  |
| PLK2 | Level 2 |  |  |
| ADGRV1 | Level 2 |  |  |
| ARRDC3 | Level 2 |  |  |
| FBXL17 | Level 2 |  |  |
| DUSP1 | Level 2 |  |  |
| BNIP1 | Level 2 |  |  |
| RNF130 | Level 2 |  |  |
| RNF144B | Level 2 |  |  |
| KCNQ5 | Level 2 |  |  |
| BCKDHB | Level 2 |  |  |
| FOXO3 | Level 2 |  |  |
| FRK | Level 2 |  |  |
| DCBLD1 | Level 2 |  |  |
| GJA1 | Level 2 |  |  |
| SMOC2 | Level 2 |  |  |
| FOXK1 | Level 2 |  |  |
| HDAC9 | Level 2 |  |  |
| CREB5 | Level 2 |  |  |
| EEPD1 | Level 2 |  |  |
| GCK | Level 2 |  |  |
| YKT6 | Level 2 |  |  |
| CCM2 | Level 2 |  |  |
| TNS3 | Level 2 |  |  |
| GRB10 | Level 2 |  |  |
| LIMK1 | Level 2 |  |  |
| STEAP2 | Level 2 |  |  |
| CDK14 | Level 2 |  |  |
| SEM1 | Level 2 |  |  |
| MUC12 | Level 2 |  |  |
| COG5 | Level 2 |  |  |
| CAPZA2 | Level 2 |  |  |
| ZNF467 | Level 2 |  |  |
| MCPH1 | Level 2 |  |  |
| SLC7A2 | Level 2 |  |  |
| SORBS3 | Level 2 |  |  |
| RBPMS | Level 2 |  |  |
| NRG1 | Level 2 |  |  |
| MAK16 | Level 2 |  |  |
| TTI2 | Level 2 |  |  |
| RNF122 | Level 2 |  |  |
| SLC20A2 | Level 2 |  |  |
| PREX2 | Level 2 |  |  |
| RDH10 | Level 2 |  |  |
| RALYL | Level 2 |  |  |
| PDP1 | Level 2 |  |  |
| CCN3 | Level 2 |  |  |
| TRAPPC9 | Level 2 |  |  |
| PLEC | Level 2 |  |  |
| KANK1 | Level 2 |  |  |
| PLIN2 | Level 2 |  |  |
| C9ORF170 | Level 2 |  |  |
| ZNF462 | Level 2 |  |  |
| ZNF618 | Level 2 |  |  |
| PAPPA | Level 2 |  |  |
| RABGAP1 | Level 2 |  |  |
| NEK6 | Level 2 |  |  |
| SCAI | Level 2 |  |  |
| PPP6C | Level 2 |  |  |
| CELF2 | Level 2 |  |  |
| PLXDC2 | Level 2 |  |  |
| NEBL | Level 2 |  |  |
| WAC | Level 2 |  |  |
| ZNF438 | Level 2 |  |  |
| ARHGAP12 | Level 2 |  |  |
| ANK3 | Level 2 |  |  |
| REEP3 | Level 2 |  |  |
| SIRT1 | Level 2 |  |  |
| RNLS | Level 2 |  |  |
| ACTA2 | Level 2 |  |  |
| BTRC | Level 2 |  |  |
| LDB1 | Level 2 |  |  |
| TCF7L2 | Level 2 |  |  |
| ARNTL | Level 2 |  |  |
| BDNF | Level 2 |  |  |
| CREB3L1 | Level 2 |  |  |
| PTPMT1 | Level 2 |  |  |
| CTNND1 | Level 2 |  |  |
| EHBP1L1 | Level 2 |  |  |
| CLPB | Level 2 |  |  |
| FAM168A | Level 2 |  |  |
| POLD3 | Level 2 |  |  |
| THAP12 | Level 2 |  |  |
| GAB2 | Level 2 |  |  |
| NOX4 | Level 2 |  |  |
| FAM76B | Level 2 |  |  |
| YAP1 | Level 2 |  |  |
| CEP164 | Level 2 |  |  |
| UBASH3B | Level 2 |  |  |
| DUSP16 | Level 2 |  |  |
| BCAT1 | Level 2 |  |  |
| STK38L | Level 2 |  |  |
| PPHLN1 | Level 2 |  |  |
| PRKAG1 | Level 2 |  |  |
| NACA | Level 2 |  |  |
| LRP1 | Level 2 |  |  |
| TMBIM4 | Level 2 |  |  |
| CAND1 | Level 2 |  |  |
| RAB3IP | Level 2 |  |  |
| CNOT2 | Level 2 |  |  |
| CEP83 | Level 2 |  |  |
| RPL6 | Level 2 |  |  |
| SBNO1 | Level 2 |  |  |
| WASF3 | Level 2 |  |  |
| MRPS31 | Level 2 |  |  |
| PRR20A | Level 2 |  |  |
| PCDH17 | Level 2 |  |  |
| DACH1 | Level 2 |  |  |
| OBI1 | Level 2 |  |  |
| SPRY2 | Level 2 |  |  |
| MBNL2 | Level 2 |  |  |
| MCF2L | Level 2 |  |  |
| SNX6 | Level 2 |  |  |
| FBXO33 | Level 2 |  |  |
| CDKL1 | Level 2 |  |  |
| FERMT2 | Level 2 |  |  |
| PPM1A | Level 2 |  |  |
| PLEKHH1 | Level 2 |  |  |
| FOXN3 | Level 2 |  |  |
| YY1 | Level 2 |  |  |
| ATP10A | Level 2 |  |  |
| MAPKBP1 | Level 2 |  |  |
| TRPM7 | Level 2 |  |  |
| PIAS1 | Level 2 |  |  |
| CCDC33 | Level 2 |  |  |
| MORF4L1 | Level 2 |  |  |
| AKAP13 | Level 2 |  |  |
| IDH2 | Level 2 |  |  |
| SLCO3A1 | Level 2 |  |  |
| CHD2 | Level 2 |  |  |
| MCTP2 | Level 2 |  |  |
| UBE2I | Level 2 |  |  |
| SLC9A3R2 | Level 2 |  |  |
| MYH11 | Level 2 |  |  |
| CNEP1R1 | Level 2 |  |  |
| NKD1 | Level 2 |  |  |
| CNOT1 | Level 2 |  |  |
| PDP2 | Level 2 |  |  |
| VAC14 | Level 2 |  |  |
| ZFHX3 | Level 2 |  |  |
| CDYL2 | Level 2 |  |  |
| BANP | Level 2 |  |  |
| ATP2A3 | Level 2 |  |  |
| PITPNM3 | Level 2 |  |  |
| KIAA0753 | Level 2 |  |  |
| TP53 | Level 2 |  |  |
| SMCR8 | Level 2 |  |  |
| SUPT6H | Level 2 |  |  |
| ERAL1 | Level 2 |  |  |
| ATAD5 | Level 2 |  |  |
| GPATCH8 | Level 2 |  |  |
| KANSL1 | Level 2 |  |  |
| MSI2 | Level 2 |  |  |
| VMP1 | Level 2 |  |  |
| C17ORF82 | Level 2 |  |  |
| MRC2 | Level 2 |  |  |
| PECAM1 | Level 2 |  |  |
| APOH | Level 2 |  |  |
| FHOD3 | Level 2 |  |  |
| SLC14A2 | Level 2 |  |  |
| TCF4 | Level 2 |  |  |
| NEDD4L | Level 2 |  |  |
| ZCCHC2 | Level 2 |  |  |
| DAZAP1 | Level 2 |  |  |
| ZBTB7A | Level 2 |  |  |
| MAP2K2 | Level 2 |  |  |
| MRPL4 | Level 2 |  |  |
| ZNF101 | Level 2 |  |  |
| CCNE1 | Level 2 |  |  |
| TSHZ3 | Level 2 |  |  |
| KIAA0355 | Level 2 |  |  |
| AKT2 | Level 2 |  |  |
| SPTBN4 | Level 2 |  |  |
| TMEM239 | Level 2 |  |  |
| BTBD3 | Level 2 |  |  |
| ID1 | Level 2 |  |  |
| DNMT3B | Level 2 |  |  |
| CDK5RAP1 | Level 2 |  |  |
| DYNLRB1 | Level 2 |  |  |
| PROCR | Level 2 |  |  |
| CHD6 | Level 2 |  |  |
| APC | Level 2 |  |  |
| EVA1C | Level 2 |  |  |
| UMODL1 | Level 2 |  |  |
| COL6A1 | Level 2 |  |  |
| DIP2A | Level 2 |  |  |
| ARVCF | Level 2 |  |  |
| TTC28 | Level 2 |  |  |
| TRIOBP | Level 2 |  |  |
| TNRC6B | Level 2 |  |  |
| XRCC6 | Level 2 |  |  |
| BRD1 | Level 2 |  |  |
| PPP2R5E | Level 2 |  |  |
| TSC22D3 | Level 2 |  |  |
| CEBPA | Level 2 |  |  |
| LMNA | Level 2 |  |  |
| DNAJC16 | Level 2 |  |  |
| YOD1 | Level 2 |  |  |
| GPN1 | Level 2 |  |  |
| PPP4R3B | Level 2 |  |  |
| SMARCC1 | Level 2 |  |  |
| EAF2 | Level 2 |  |  |
| AIMP1 | Level 2 |  |  |
| USP53 | Level 2 |  |  |
| H2AC4 | Level 2 |  |  |
| CCHCR1 | Level 2 |  |  |
| CSNK2B | Level 2 |  |  |
| GOPC | Level 2 |  |  |
| FDFT1 | Level 2 |  |  |
| ZBTB6 | Level 2 |  |  |
| GAPVD1 | Level 2 |  |  |
| JMJD1C | Level 2 |  |  |
| SEC24C | Level 2 |  |  |
| PRXL2A | Level 2 |  |  |
| PDCD11 | Level 2 |  |  |
| PPCDC | Level 2 |  |  |
| CMTM3 | Level 2 |  |  |
| SRR | Level 2 |  |  |
| PLEKHM1 | Level 2 |  |  |
| MAPT | Level 2 |  |  |
| SCRN2 | Level 2 |  |  |
| DDX5 | Level 2 |  |  |
| DNAJC5 | Level 2 |  |  |
| HAX1 | Level 2 |  |  |
| ORC4 | Level 2 |  |  |
| AMOTL2 | Level 2 |  |  |
| HLA-A | Level 2 |  |  |
| VARS2 | Level 2 |  |  |
| HLA-DQB2 | Level 2 |  |  |
| FAM91A1 | Level 2 |  |  |
| TLN1 | Level 2 |  |  |
| ACTR1A | Level 2 |  |  |
| BNIP3 | Level 2 |  |  |
| ATP2A2 | Level 2 |  |  |
| MAPKAPK5 | Level 2 |  |  |
| TRAFD1 | Level 2 |  |  |
| CDK2AP1 | Level 2 |  |  |
| MPI | Level 2 |  |  |
| RHOT2 | Level 2 |  |  |
| POLI | Level 2 |  |  |
| ZC3H4 | Level 2 |  |  |
| RTEL1 | Level 2 |  |  |
| RSF1 | Level 2 |  |  |
| SNTB2 | Level 2 |  |  |
| SPATA2L | Level 2 |  |  |
| SHKBP1 | Level 2 |  |  |
| UCKL1 | Level 2 |  |  |
| HLA-DRA | Level 2 |  |  |
| AP3M1 | Level 2 |  |  |
| MPHOSPH9 | Level 2 |  |  |
| HM13 | Level 2 |  |  |
| AARS1 | Level 2 |  |  |
| ACOT8 | Level 2 |  |  |
| ADAM10 | Level 2 |  |  |
| HYKK | Level 2 |  |  |
| AMBRA1 | Level 2 |  |  |
| ANGPTL4 | Level 2 |  |  |
| ANTXR1 | Level 2 |  |  |
| AP1G1 | Level 2 |  |  |
| ARHGAP1 | Level 2 |  |  |
| ARIH1 | Level 2 |  |  |
| ASZ1 | Level 2 |  |  |
| ATG13 | Level 2 |  |  |
| ATXN1L | Level 2 |  |  |
| BAD | Level 2 |  |  |
| C1ORF105 | Level 2 |  |  |
| C1ORF43 | Level 2 |  |  |
| CADM4 | Level 2 |  |  |
| CAMK2G | Level 2 |  |  |
| CARM1 | Level 2 |  |  |
| CCDC36 | Level 2 |  |  |
| CCDC88B | Level 2 |  |  |
| CD40 | Level 2 |  |  |
| CELF1 | Level 2 |  |  |
| CEP57 | Level 2 |  |  |
| CHRM4 | Level 2 |  |  |
| CKAP5 | Level 2 |  |  |
| CLPTM1 | Level 2 |  |  |
| CLTC | Level 2 |  |  |
| CNPY4 | Level 2 |  |  |
| CPA4 | Level 2 |  |  |
| CRTC2 | Level 2 |  |  |
| CTTNBP2NL | Level 2 |  |  |
| CYP2C9 | Level 2 |  |  |
| DAAM1 | Level 2 |  |  |
| DAB2IP | Level 2 |  |  |
| DAG1 | Level 2 |  |  |
| DCAF7 | Level 2 |  |  |
| DDX19A | Level 2 |  |  |
| DDX19B | Level 2 |  |  |
| DDX42 | Level 2 |  |  |
| DGKZ | Level 2 |  |  |
| DHX38 | Level 2 |  |  |
| DNTTIP1 | Level 2 |  |  |
| ECM1 | Level 2 |  |  |
| EIF4A1 | Level 2 |  |  |
| EPOR | Level 2 |  |  |
| ERG | Level 2 |  |  |
| ESRRA | Level 2 |  |  |
| FBRS | Level 2 |  |  |
| FGF11 | Level 2 |  |  |
| FUS | Level 2 |  |  |
| FXR2 | Level 2 |  |  |
| CASTOR1 | Level 2 |  |  |
| GCA | Level 2 |  |  |
| GMPPB | Level 2 |  |  |
| GPX1 | Level 2 |  |  |
| GSN | Level 2 |  |  |
| HEATR6 | Level 2 |  |  |
| HELLS | Level 2 |  |  |
| HERPUD1 | Level 2 |  |  |
| H1-2 | Level 2 |  |  |
| H1-3 | Level 2 |  |  |
| H1-4 | Level 2 |  |  |
| H2AC7 | Level 2 |  |  |
| H2AC8 | Level 2 |  |  |
| H2BC5 | Level 2 |  |  |
| H2BC9 | Level 2 |  |  |
| H3C3 | Level 2 |  |  |
| H3C4 | Level 2 |  |  |
| H3C6 | Level 2 |  |  |
| H3C7 | Level 2 |  |  |
| H3C8 | Level 2 |  |  |
| H4C4 | Level 2 |  |  |
| H4C5 | Level 2 |  |  |
| H4C6 | Level 2 |  |  |
| HOXB13 | Level 2 |  |  |
| HP | Level 2 |  |  |
| HSD3B7 | Level 2 |  |  |
| HSP90AB4P | Level 2 |  |  |
| IFIH1 | Level 2 |  |  |
| IL1F10 | Level 2 |  |  |
| IL1RN | Level 2 |  |  |
| IL36RN | Level 2 |  |  |
| IP6K2 | Level 2 |  |  |
| IREB2 | Level 2 |  |  |
| IST1 | Level 2 |  |  |
| KCTD14 | Level 2 |  |  |
| KCTD9 | Level 2 |  |  |
| KLC1 | Level 2 |  |  |
| L3HYPDH | Level 2 |  |  |
| LDAH | Level 2 |  |  |
| LDLR | Level 2 |  |  |
| LPL | Level 2 |  |  |
| MACROD1 | Level 2 |  |  |
| MAP2K4 | Level 2 |  |  |
| MAP2K5 | Level 2 |  |  |
| MAP3K3 | Level 2 |  |  |
| ME2 | Level 2 |  |  |
| MEOX2 | Level 2 |  |  |
| MIA3 | Level 2 |  |  |
| MIR140 | Level 2 |  |  |
| MIR199A1 | Level 2 |  |  |
| MIR21 | Level 2 |  |  |
| MPDU1 | Level 2 |  |  |
| MPLKIP | Level 2 |  |  |
| MYO1A | Level 2 |  |  |
| MYO9A | Level 2 |  |  |
| NAB2 | Level 2 |  |  |
| NCKIPSD | Level 2 |  |  |
| NCOA5 | Level 2 |  |  |
| NEFH | Level 2 |  |  |
| NF2 | Level 2 |  |  |
| NIPSNAP1 | Level 2 |  |  |
| NLGN2 | Level 2 |  |  |
| NLRC5 | Level 2 |  |  |
| NRBF2 | Level 2 |  |  |
| NUFIP2 | Level 2 |  |  |
| P4HTM | Level 2 |  |  |
| PACSIN3 | Level 2 |  |  |
| PHLPP2 | Level 2 |  |  |
| PKM | Level 2 |  |  |
| PLAU | Level 2 |  |  |
| PLXNB1 | Level 2 |  |  |
| POLR2A | Level 2 |  |  |
| PRDX5 | Level 2 |  |  |
| PRKAR2A | Level 2 |  |  |
| PRPF3 | Level 2 |  |  |
| PSMA4 | Level 2 |  |  |
| PTRH2 | Level 2 |  |  |
| NECTIN2 | Level 2 |  |  |
| PYCARD | Level 2 |  |  |
| QARS1 | Level 2 |  |  |
| QRICH1 | Level 2 |  |  |
| RDX | Level 2 |  |  |
| RGS14 | Level 2 |  |  |
| RHBDD1 | Level 2 |  |  |
| RHOA | Level 2 |  |  |
| RPRD2 | Level 2 |  |  |
| RPS28 | Level 2 |  |  |
| RPS6KA4 | Level 2 |  |  |
| RPS6KB1 | Level 2 |  |  |
| RRP15 | Level 2 |  |  |
| SCARB1 | Level 2 |  |  |
| SDR9C7 | Level 2 |  |  |
| SENP3 | Level 2 |  |  |
| SF3A1 | Level 2 |  |  |
| SF3A2 | Level 2 |  |  |
| SLC25A20 | Level 2 |  |  |
| SLC26A6 | Level 2 |  |  |
| SLC39A1 | Level 2 |  |  |
| SMARCA4 | Level 2 |  |  |
| SMOC1 | Level 2 |  |  |
| SMYD2 | Level 2 |  |  |
| SNX32 | Level 2 |  |  |
| SORT1 | Level 2 |  |  |
| SPC24 | Level 2 |  |  |
| SPP1 | Level 2 |  |  |
| SREBF1 | Level 2 |  |  |
| STAG3L1 | Level 2 |  |  |
| STAT6 | Level 2 |  |  |
| STX1B | Level 2 |  |  |
| TARS2 | Level 2 |  |  |
| TGFBRAP1 | Level 2 |  |  |
| THOC5 | Level 2 |  |  |
| TLK2 | Level 2 |  |  |
| TMED1 | Level 2 |  |  |
| TMEM102 | Level 2 |  |  |
| TNFSF12 | Level 2 |  |  |
| TNFSF13 | Level 2 |  |  |
| TNNC2 | Level 2 |  |  |
| TOM1L2 | Level 2 |  |  |
| TRMT112 | Level 2 |  |  |
| TXNL4B | Level 2 |  |  |
| UBE2C | Level 2 |  |  |
| UBE2Q1 | Level 2 |  |  |
| USP19 | Level 2 |  |  |
| USP3 | Level 2 |  |  |
| USP4 | Level 2 |  |  |
| WAPL | Level 2 |  |  |
| WDR6 | Level 2 |  |  |
| WRAP53 | Level 2 |  |  |
| WWP2 | Level 2 |  |  |
| XRCC3 | Level 2 |  |  |
| ZFYVE21 | Level 2 |  |  |
| ZKSCAN1 | Level 2 |  |  |
| ZPR1 | Level 2 |  |  |
| ZSWIM1 | Level 2 |  |  |
| ZSWIM3 | Level 2 |  |  |
| NPC1 | Level 2 |  |  |
| IMMT | Level 2 |  |  |
| SPCS1 | Level 2 |  |  |
| GNL3 | Level 2 |  |  |
| SLC16A1 | Level 2 |  |  |
| LTA | Level 2 |  |  |
| HNRNPL | Bridging |  |  |
| TRAF2 | Bridging |  |  |
| TRAF6 | Bridging |  |  |
| PLEKHA4 | Bridging |  |  |
| RAC1 | Bridging |  |  |
| LRRFIP2 | Bridging |  |  |
| FLII | Bridging |  |  |
| PFKL | Bridging |  |  |
| TRIM28 | Bridging |  |  |
| SQSTM1 | Bridging |  |  |
| MDM2 | Bridging |  |  |
| MAP3K7 | Bridging |  |  |
| MBIP | Bridging |  |  |
| CYLD | Bridging |  |  |
| TBK1 | Bridging |  |  |
| IKBKE | Bridging |  |  |
| INS | Bridging |  |  |
| CYTH2 | Bridging |  |  |
| IRF7 | Bridging |  |  |
| POLR1C | Bridging |  |  |
| WDYHV1 | Bridging |  |  |
| RNF4 | Bridging |  |  |
| TLR9 | Bridging |  |  |
| TLR10 | Bridging |  |  |
| IKBKG | Bridging |  |  |
| SMURF1 | Bridging |  |  |
| ATG5 | Bridging |  |  |
| SYK | Bridging |  |  |
| TSG101 | Bridging |  |  |
| RIPK2 | Bridging |  |  |
| UBAP1 | Bridging |  |  |
| SMAD3 | Bridging |  |  |
| USP7 | Bridging |  |  |
| PELI1 | Bridging |  |  |
| AKT1 | Bridging |  |  |
| IL1B | Bridging |  |  |
| BANK1 | Bridging |  |  |
| TRAF3 | Bridging |  |  |
| IRAK1 | Bridging |  |  |
| TLR2 | Bridging |  |  |
| STAP2 | Bridging |  |  |
| TLR3 | Bridging |  |  |
| SIAH2 | Bridging |  |  |
| SIAH1 | Bridging |  |  |
| HDAC6 | Bridging |  |  |
| MAPK14 | Bridging |  |  |
| BST2 | Bridging |  |  |
| TNIP1 | Bridging |  |  |
| FADD | Bridging |  |  |
| CBLB | Bridging |  |  |
| CARD9 | Bridging |  |  |
| NFKBIA | Bridging |  |  |
| IL1R1 | Bridging |  |  |
| IKBKB | Bridging |  |  |
| IRF4 | Bridging |  |  |
| TNFRSF1A | Bridging |  |  |
| IL1RAP | Bridging |  |  |
| DOCK8 | Bridging |  |  |
| TXN | Bridging |  |  |
| LRRFIP1 | Bridging |  |  |
| SMAD6 | Bridging |  |  |
| PRDX1 | Bridging |  |  |
| IRF5 | Bridging |  |  |
| CD14 | Bridging |  |  |
| TLR7 | Bridging |  |  |
| BTK | Bridging |  |  |
| SPOP | Bridging |  |  |
| TNFRSF13B | Bridging |  |  |
| MAP3K1 | Bridging |  |  |
| SASH1 | Bridging |  |  |
| CISH | Bridging |  |  |
| GLOD4 | Bridging |  |  |
| MAL | Bridging |  |  |
| CASP1 | Bridging |  |  |
| NOD2 | Bridging |  |  |
| MAP4K4 | Bridging |  |  |
| NGFR | Bridging |  |  |
| TLR4 | Bridging |  |  |
| IRAK3 | Bridging |  |  |
| PTK2B | Bridging |  |  |
| SARM1 | Bridging |  |  |
| CD93 | Bridging |  |  |
| PIK3AP1 | Bridging |  |  |
| CAMLG | Bridging |  |  |
| IRAK2 | Bridging |  |  |
| IRAK4 | Bridging |  |  |
| ARMCX6 | Bridging |  |  |
| TLR8 | Bridging |  |  |
| TIRAP | Bridging |  |  |
| ZNF35 | Bridging |  |  |
| PELI2 | Bridging |  |  |
| IL1RL2 | Bridging |  |  |
| TLR5 | Bridging |  |  |
| AGER | Bridging |  |  |
| RNF152 | Bridging |  |  |
| SPOPL | Bridging |  |  |
| IL1RL1 | Bridging |  |  |
| SLC9A3R2 | Level 2 | Backman, 2021 | 34662886 |
| SLC27A3 | Level 2 |  |  |
| PKD1 | Level 2 |  |  |
| SMAD3 | Bridging |  |  |
| SIAH1 | Bridging |  |  |
| PLEKHA4 | Bridging |  |  |
| AKT1 | Bridging |  |  |
| CNNM2 | Level 2 | Cho, 2021 | 34828409 |
| ATP2B1 | Level 2 |  |  |
| ALDH2 | Level 2 |  |  |
| INO80 | Level 2 |  |  |
| PLEKHA4 | Bridging |  |  |
| RAC1 | Bridging |  |  |
| HNRNPL | Bridging |  |  |
| RNF4 | Bridging |  |  |
| MAP3K7 | Bridging |  |  |
| HIVEP3 | Level 2 | Ehret, 2016 | 27618452 |
| PNPT1 | Level 2 |  |  |
| FGD5 | Level 2 |  |  |
| TBC1D1 | Level 2 |  |  |
| CSNK1G3 | Level 2 |  |  |
| ZC3HC1 | Level 2 |  |  |
| PSMD5 | Level 2 |  |  |
| PSMC3 | Level 2 |  |  |
| LRRC10B | Level 2 |  |  |
| SETBP1 | Level 2 |  |  |
| INSR | Level 2 |  |  |
| CRYAA | Level 2 |  |  |
| SIK1 | Level 2 |  |  |
| CASZ1 | Level 2 |  |  |
| MTHFR | Level 2 |  |  |
| CAPZA1 | Level 2 |  |  |
| MOV10 | Level 2 |  |  |
| MDM4 | Level 2 |  |  |
| NCAPH | Level 2 |  |  |
| GRB14 | Level 2 |  |  |
| ATG7 | Level 2 |  |  |
| SLC4A7 | Level 2 |  |  |
| ULK4 | Level 2 |  |  |
| MAP4 | Level 2 |  |  |
| MECOM | Level 2 |  |  |
| GUCY1A1 | Level 2 |  |  |
| GUCY1B1 | Level 2 |  |  |
| NPR3 | Level 2 |  |  |
| HFE | Level 2 |  |  |
| PRRC2A | Level 2 |  |  |
| ABHD16A | Level 2 |  |  |
| ZNF318 | Level 2 |  |  |
| PLEKHG1 | Level 2 |  |  |
| BLK | Level 2 |  |  |
| CACNB2 | Level 2 |  |  |
| PLCE1 | Level 2 |  |  |
| NT5C2 | Level 2 |  |  |
| LSP1 | Level 2 |  |  |
| TNNT3 | Level 2 |  |  |
| PLEKHA7 | Level 2 |  |  |
| SIPA1 | Level 2 |  |  |
| ARHGAP42 | Level 2 |  |  |
| PDE3A | Level 2 |  |  |
| ATP2B1 | Level 2 |  |  |
| TBX3 | Level 2 |  |  |
| ULK3 | Level 2 |  |  |
| FURIN | Level 2 |  |  |
| FES | Level 2 |  |  |
| GOSR2 | Level 2 |  |  |
| ZNF652 | Level 2 |  |  |
| JAG1 | Level 2 |  |  |
| GNAS | Level 2 |  |  |
| HNRNPL | Bridging |  |  |
| TRAF2 | Bridging |  |  |
| TRAF6 | Bridging |  |  |
| PLEKHA4 | Bridging |  |  |
| RAC1 | Bridging |  |  |
| LRRFIP2 | Bridging |  |  |
| FLII | Bridging |  |  |
| PFKL | Bridging |  |  |
| TRIM28 | Bridging |  |  |
| SQSTM1 | Bridging |  |  |
| MDM2 | Bridging |  |  |
| MAP3K7 | Bridging |  |  |
| MBIP | Bridging |  |  |
| CYLD | Bridging |  |  |
| TBK1 | Bridging |  |  |
| IKBKE | Bridging |  |  |
| INS | Bridging |  |  |
| CYTH2 | Bridging |  |  |
| IRF7 | Bridging |  |  |
| POLR1C | Bridging |  |  |
| WDYHV1 | Bridging |  |  |
| RNF4 | Bridging |  |  |
| TLR9 | Bridging |  |  |
| TLR10 | Bridging |  |  |
| IKBKG | Bridging |  |  |
| SMURF1 | Bridging |  |  |
| ATG5 | Bridging |  |  |
| SYK | Bridging |  |  |
| TSG101 | Bridging |  |  |
| RIPK2 | Bridging |  |  |
| UBAP1 | Bridging |  |  |
| SMAD3 | Bridging |  |  |
| USP7 | Bridging |  |  |
| PELI1 | Bridging |  |  |
| AKT1 | Bridging |  |  |
| IL1B | Bridging |  |  |
| BANK1 | Bridging |  |  |
| TRAF3 | Bridging |  |  |
| IRAK1 | Bridging |  |  |
| TLR2 | Bridging |  |  |
| STAP2 | Bridging |  |  |
| TLR3 | Bridging |  |  |
| SIAH2 | Bridging |  |  |
| SIAH1 | Bridging |  |  |
| HDAC6 | Bridging |  |  |
| MAPK14 | Bridging |  |  |
| BST2 | Bridging |  |  |
| TNIP1 | Bridging |  |  |
| FADD | Bridging |  |  |
| L2HGDH | Level 2 | Evangelou, 2018 | 30224653 |
| PBX3 | Level 2 |  |  |
| KDM4B | Level 2 |  |  |
| YES1 | Level 2 |  |  |
| LRP4 | Level 2 |  |  |
| TERT | Level 2 |  |  |
| MARK3 | Level 2 |  |  |
| NSD3 | Level 2 |  |  |
| TOP3A | Level 2 |  |  |
| ATP2B1 | Level 2 |  |  |
| MSRA | Level 2 |  |  |
| FAM193A | Level 2 |  |  |
| INPP5A | Level 2 |  |  |
| WDR7 | Level 2 |  |  |
| CDKAL1 | Level 2 |  |  |
| NFATC2 | Level 2 |  |  |
| MEX3C | Level 2 |  |  |
| ARHGEF25 | Level 2 |  |  |
| TGFBR2 | Level 2 |  |  |
| KLF5 | Level 2 |  |  |
| PDE8A | Level 2 |  |  |
| IGF1 | Level 2 |  |  |
| SYT1 | Level 2 |  |  |
| BCL2 | Level 2 |  |  |
| THADA | Level 2 |  |  |
| XPR1 | Level 2 |  |  |
| ZMAT2 | Level 2 |  |  |
| MEF2A | Level 2 |  |  |
| PTPRD | Level 2 |  |  |
| FOXC1 | Level 2 |  |  |
| PLXNB2 | Level 2 |  |  |
| EPB41L2 | Level 2 |  |  |
| LRCH1 | Level 2 |  |  |
| PPP2R2D | Level 2 |  |  |
| AQP1 | Level 2 |  |  |
| CTNNB1 | Level 2 |  |  |
| ARL14EP | Level 2 |  |  |
| TNKS | Level 2 |  |  |
| CENPP | Level 2 |  |  |
| FARP2 | Level 2 |  |  |
| TSNARE1 | Level 2 |  |  |
| BMPR1B | Level 2 |  |  |
| NIBAN2 | Level 2 |  |  |
| MERTK | Level 2 |  |  |
| RAD52 | Level 2 |  |  |
| WNT4 | Level 2 |  |  |
| CITED2 | Level 2 |  |  |
| KAT2B | Level 2 |  |  |
| POM121C | Level 2 |  |  |
| BCAR3 | Level 2 |  |  |
| HSPA12A | Level 2 |  |  |
| BCAS3 | Level 2 |  |  |
| GABRA2 | Level 2 |  |  |
| ARHGAP29 | Level 2 |  |  |
| TRIP12 | Level 2 |  |  |
| ERBB4 | Level 2 |  |  |
| JAZF1 | Level 2 |  |  |
| IRF6 | Level 2 |  |  |
| DGKH | Level 2 |  |  |
| MLF1 | Level 2 |  |  |
| SPIB | Level 2 |  |  |
| ARIH2 | Level 2 |  |  |
| PRKD1 | Level 2 |  |  |
| SOX5 | Level 2 |  |  |
| RTN4 | Level 2 |  |  |
| DENND2B | Level 2 |  |  |
| SREK1 | Level 2 |  |  |
| TARS1 | Level 2 |  |  |
| RBFOX1 | Level 2 |  |  |
| RBM26 | Level 2 |  |  |
| CAPRIN1 | Level 2 |  |  |
| SKI | Level 2 |  |  |
| CCT6A | Level 2 |  |  |
| ZBTB20 | Level 2 |  |  |
| TASOR2 | Level 2 |  |  |
| HNRNPL | Bridging |  |  |
| PLEKHA4 | Bridging |  |  |
| TRAF6 | Bridging |  |  |
| AKT1 | Bridging |  |  |
| CBLB | Bridging |  |  |
| MAPK14 | Bridging |  |  |
| RAC1 | Bridging |  |  |
| TRAF2 | Bridging |  |  |
| TRIM28 | Bridging |  |  |
| STAP2 | Bridging |  |  |
| CARD9 | Bridging |  |  |
| NFKBIA | Bridging |  |  |
| IL1R1 | Bridging |  |  |
| MDM2 | Bridging |  |  |
| IKBKB | Bridging |  |  |
| RNF4 | Bridging |  |  |
| SQSTM1 | Bridging |  |  |
| INS | Bridging |  |  |
| IRF4 | Bridging |  |  |
| RIPK2 | Bridging |  |  |
| POLR1C | Bridging |  |  |
| USP7 | Bridging |  |  |
| SMAD3 | Bridging |  |  |
| MAP3K7 | Bridging |  |  |
| SMURF1 | Bridging |  |  |
| IKBKG | Bridging |  |  |
| TNFRSF1A | Bridging |  |  |
| TNIP1 | Bridging |  |  |
| IL1RAP | Bridging |  |  |
| TBK1 | Bridging |  |  |
| DOCK8 | Bridging |  |  |
| SYK | Bridging |  |  |
| SIAH1 | Bridging |  |  |
| TXN | Bridging |  |  |
| HDAC6 | Bridging |  |  |
| LRRFIP1 | Bridging |  |  |
| LRRFIP2 | Bridging |  |  |
| CYLD | Bridging |  |  |
| FLII | Bridging |  |  |
| WDYHV1 | Bridging |  |  |
| SMAD6 | Bridging |  |  |
| PRDX1 | Bridging |  |  |
| SIAH2 | Bridging |  |  |
| MBIP | Bridging |  |  |
| IRF7 | Bridging |  |  |
| IRF5 | Bridging |  |  |
| TSG101 | Bridging |  |  |
| CD14 | Bridging |  |  |
| IRAK1 | Bridging |  |  |
| TLR7 | Bridging |  |  |
| BTK | Bridging |  |  |
| SPOP | Bridging |  |  |
| IKBKE | Bridging |  |  |
| IL1B | Bridging |  |  |
| TNFRSF13B | Bridging |  |  |
| MAP3K1 | Bridging |  |  |
| GFPT1 | Level 2 | Huang, 2022 | 36438602 |
| RBM6 | Level 2 |  |  |
| ZNF589 | Level 2 |  |  |
| UHRF1BP1 | Level 2 |  |  |
| NSUN6 | Level 2 |  |  |
| SFXN4 | Level 2 |  |  |
| TNNT3 | Level 2 |  |  |
| LYZ | Level 2 |  |  |
| CDC16 | Level 2 |  |  |
| EXOSC6 | Level 2 |  |  |
| CRLF3 | Level 2 |  |  |
| ZNF100 | Level 2 |  |  |
| INS | Bridging |  |  |
| TRIM28 | Bridging |  |  |
| PLEKHA4 | Bridging |  |  |
| HNRNPL | Bridging |  |  |
| TSG101 | Bridging |  |  |
| MAPK14 | Bridging |  |  |
| IKBKB | Bridging |  |  |
| RNF4 | Bridging |  |  |
| SPOP | Bridging |  |  |
| SMAD6 | Bridging |  |  |
| CYLD | Bridging |  |  |
| SMAD3 | Bridging |  |  |
| MDM2 | Bridging |  |  |
| DOCK8 | Bridging |  |  |
| LEF1 | Level 2 | Huang_2023 | 36845374 |
| MAPK4 | Level 2 |  |  |
| SMAD3 | Bridging |  |  |
| IRAK1 | Bridging |  |  |
| AKT1 | Bridging |  |  |
| PDE3A | Level 2 | Kato, 2015 | 26390057 |
| AKT1 | Bridging |  |  |
| TRAF3 | Level 1 | Keaton, 2024 | 38689001 |
| NFATC1 | Level 2 |  |  |
| ZBTB34 | Level 2 |  |  |
| YWHAQ | Level 2 |  |  |
| GHR | Level 2 |  |  |
| MBTPS1 | Level 2 |  |  |
| NUP93 | Level 2 |  |  |
| TENT5C | Level 2 |  |  |
| GADD45A | Level 2 |  |  |
| NR4A1 | Level 2 |  |  |
| TRPC4 | Level 2 |  |  |
| EIF4G3 | Level 2 |  |  |
| IL20RB | Level 2 |  |  |
| RAP1A | Level 2 |  |  |
| SUPT16H | Level 2 |  |  |
| FAT1 | Level 2 |  |  |
| ZNF227 | Level 2 |  |  |
| TFAP2A | Level 2 |  |  |
| CAP2 | Level 2 |  |  |
| SIPA1L1 | Level 2 |  |  |
| MEX3B | Level 2 |  |  |
| SENP8 | Level 2 |  |  |
| PDE4D | Level 2 |  |  |
| NFIL3 | Level 2 |  |  |
| OXNAD1 | Level 2 |  |  |
| ZNF250 | Level 2 |  |  |
| HIGD1C | Level 2 |  |  |
| UBE3C | Level 2 |  |  |
| ACTN3 | Level 2 |  |  |
| GPAT3 | Level 2 |  |  |
| DAB2 | Level 2 |  |  |
| TLE1 | Level 2 |  |  |
| NFIA | Level 2 |  |  |
| SMAD7 | Level 2 |  |  |
| RNF165 | Level 2 |  |  |
| SPTBN1 | Level 2 |  |  |
| TRIB2 | Level 2 |  |  |
| MIR138-1 | Level 2 |  |  |
| BIRC6 | Level 2 |  |  |
| UGGT1 | Level 2 |  |  |
| TEK | Level 2 |  |  |
| NAV2 | Level 2 |  |  |
| DHX57 | Level 2 |  |  |
| FOXP1 | Level 2 |  |  |
| MTF2 | Level 2 |  |  |
| CIT | Level 2 |  |  |
| BIN1 | Level 2 |  |  |
| NFIB | Level 2 |  |  |
| SH3YL1 | Level 2 |  |  |
| ABL1 | Level 2 |  |  |
| BCL6 | Level 2 |  |  |
| ARHGAP20 | Level 2 |  |  |
| MITF | Level 2 |  |  |
| GPC6 | Level 2 |  |  |
| SCAF11 | Level 2 |  |  |
| TRIM33 | Level 2 |  |  |
| CEP78 | Level 2 |  |  |
| SLC39A10 | Level 2 |  |  |
| RNF213 | Level 2 |  |  |
| FARP1 | Level 2 |  |  |
| ETV6 | Level 2 |  |  |
| TMPRSS6 | Level 2 |  |  |
| MAPK14 | Bridging |  |  |
| HNRNPL | Bridging |  |  |
| RNF4 | Bridging |  |  |
| INS | Bridging |  |  |
| BANK1 | Bridging |  |  |
| TRAF6 | Bridging |  |  |
| MDM2 | Bridging |  |  |
| TRIM28 | Bridging |  |  |
| RIPK2 | Bridging |  |  |
| SMURF1 | Bridging |  |  |
| SPOP | Bridging |  |  |
| AKT1 | Bridging |  |  |
| PRDX1 | Bridging |  |  |
| CYLD | Bridging |  |  |
| PFKL | Bridging |  |  |
| MAP3K1 | Bridging |  |  |
| PLEKHA4 | Bridging |  |  |
| SASH1 | Bridging |  |  |
| CBLB | Bridging |  |  |
| CISH | Bridging |  |  |
| SQSTM1 | Bridging |  |  |
| SMAD3 | Bridging |  |  |
| TRAF2 | Bridging |  |  |
| HDAC6 | Bridging |  |  |
| GLOD4 | Bridging |  |  |
| TNFRSF1A | Bridging |  |  |
| WDYHV1 | Bridging |  |  |
| MAL | Bridging |  |  |
| FADD | Bridging |  |  |
| RAC1 | Bridging |  |  |
| CASP1 | Bridging |  |  |
| PELI1 | Bridging |  |  |
| TBK1 | Bridging |  |  |
| CARD9 | Bridging |  |  |
| IRF4 | Bridging |  |  |
| NOD2 | Bridging |  |  |
| MAP3K7 | Bridging |  |  |
| SMAD6 | Bridging |  |  |
| IKBKE | Bridging |  |  |
| TLR9 | Bridging |  |  |
| IKBKG | Bridging |  |  |
| SYK | Bridging |  |  |
| TXN | Bridging |  |  |
| IRF5 | Bridging |  |  |
| POLR1C | Bridging |  |  |
| BTK | Bridging |  |  |
| USP7 | Bridging |  |  |
| NFKBIA | Bridging |  |  |
| SIAH1 | Bridging |  |  |
| ATG5 | Bridging |  |  |
| MAP4K4 | Bridging |  |  |
| IL1RAP | Bridging |  |  |
| IKBKB | Bridging |  |  |
| TNIP1 | Bridging |  |  |
| NGFR | Bridging |  |  |
| TLR4 | Bridging |  |  |
| TNFRSF13B | Bridging |  |  |
| TLR3 | Bridging |  |  |
| TRAF3 | Bridging |  |  |
| SYT7 | Level 2 | Kelly, 2022 | 35652341 |
| SMARCD1 | Level 2 |  |  |
| RBM45 | Level 2 |  |  |
| GPR156 | Level 2 |  |  |
| ERG | Level 2 |  |  |
| INS | Bridging |  |  |
| SIAH1 | Bridging |  |  |
| IRF4 | Bridging |  |  |
| SQSTM1 | Bridging |  |  |
| PRDX1 | Bridging |  |  |
| HNRNPL | Bridging |  |  |
| TRIM28 | Bridging |  |  |
| USP7 | Bridging |  |  |
| WDYHV1 | Bridging |  |  |
| PLEKHA4 | Bridging |  |  |
| MAPK14 | Bridging |  |  |
| SPOP | Bridging |  |  |
| RERE | Level 2 | Li, 2024 | 39090212 |
| NUCKS1 | Level 2 |  |  |
| ARL14 | Level 2 |  |  |
| MECOM | Level 2 |  |  |
| COPS4 | Level 2 |  |  |
| HBS1L | Level 2 |  |  |
| UHRF2 | Level 2 |  |  |
| CACNB2 | Level 2 |  |  |
| CLNS1A | Level 2 |  |  |
| ALKBH5 | Level 2 |  |  |
| CDC27 | Level 2 |  |  |
| ZNF652 | Level 2 |  |  |
| NRIP1 | Level 2 |  |  |
| HNRNPL | Bridging |  |  |
| RNF4 | Bridging |  |  |
| MBIP | Bridging |  |  |
| MDM2 | Bridging |  |  |
| SMURF1 | Bridging |  |  |
| SMAD3 | Bridging |  |  |
| SQSTM1 | Bridging |  |  |
| PFKL | Bridging |  |  |
| IKBKB | Bridging |  |  |
| IRF5 | Bridging |  |  |
| PLEKHA4 | Bridging |  |  |
| INS | Bridging |  |  |
| MAP3K1 | Bridging |  |  |
| USP7 | Bridging |  |  |
| RAC1 | Bridging |  |  |
| CYLD | Bridging |  |  |
| TRAF2 | Bridging |  |  |
| MAP3K7 | Bridging |  |  |
| MTHFR | Level 2 | Newton-Cheh, 2009 | 19219041 |
| ZNF652 | Level 2 |  |  |
| TLR9 | Bridging |  |  |
| TLR10 | Bridging |  |  |
| RNF4 | Bridging |  |  |
| NFKBIA | Level 1 | OTP hypertension | 36399499 |
| IKBKB | Level 1 |  |  |
| TLR9 | Level 1 |  |  |
| TLR7 | Level 1 |  |  |
| MAP3K1 | Level 1 |  |  |
| RAC1 | Level 1 |  |  |
| UBAP1 | Level 1 |  |  |
| SARM1 | Level 1 |  |  |
| BANK1 | Level 1 |  |  |
| SMAD6 | Level 1 |  |  |
| IL1B | Level 1 |  |  |
| TLR2 | Level 1 |  |  |
| TLR10 | Level 1 |  |  |
| IL1R1 | Level 1 |  |  |
| TXN | Level 1 |  |  |
| FADD | Level 1 |  |  |
| BMPR2 | Level 2 |  |  |
| AGTR1 | Level 2 |  |  |
| EIF2AK4 | Level 2 |  |  |
| SARS2 | Level 2 |  |  |
| SMAD9 | Level 2 |  |  |
| CPS1 | Level 2 |  |  |
| GUCY1A1 | Level 2 |  |  |
| CAV1 | Level 2 |  |  |
| GUCY1B1 | Level 2 |  |  |
| PKD1 | Level 2 |  |  |
| ATP13A3 | Level 2 |  |  |
| ENG | Level 2 |  |  |
| CACNA1C | Level 2 |  |  |
| ADRB2 | Level 2 |  |  |
| CA2 | Level 2 |  |  |
| SCNN1A | Level 2 |  |  |
| ROCK2 | Level 2 |  |  |
| ROCK1 | Level 2 |  |  |
| NR3C1 | Level 2 |  |  |
| INSR | Level 2 |  |  |
| ARHGAP42 | Level 2 |  |  |
| SCN8A | Level 2 |  |  |
| GABRA2 | Level 2 |  |  |
| CHRM3 | Level 2 |  |  |
| GABRE | Level 2 |  |  |
| PLEKHG1 | Level 2 |  |  |
| SLC7A1 | Level 2 |  |  |
| CACNB2 | Level 2 |  |  |
| NISCH | Level 2 |  |  |
| PGR | Level 2 |  |  |
| FES | Level 2 |  |  |
| NPR3 | Level 2 |  |  |
| NDUFA7 | Level 2 |  |  |
| SWAP70 | Level 2 |  |  |
| NDUFS4 | Level 2 |  |  |
| PLCE1 | Level 2 |  |  |
| KDR | Level 2 |  |  |
| CELA2A | Level 2 |  |  |
| NPNT | Level 2 |  |  |
| CASZ1 | Level 2 |  |  |
| NDUFAB1 | Level 2 |  |  |
| NDUFB4 | Level 2 |  |  |
| NDUFA6 | Level 2 |  |  |
| NDUFA8 | Level 2 |  |  |
| NDUFA4 | Level 2 |  |  |
| NDUFB10 | Level 2 |  |  |
| NDUFA9 | Level 2 |  |  |
| NDUFS3 | Level 2 |  |  |
| NDUFS6 | Level 2 |  |  |
| NDUFA5 | Level 2 |  |  |
| NDUFA12 | Level 2 |  |  |
| NDUFV3 | Level 2 |  |  |
| NDUFB5 | Level 2 |  |  |
| NDUFS2 | Level 2 |  |  |
| NDUFA13 | Level 2 |  |  |
| NDUFAF4 | Level 2 |  |  |
| NDUFC2 | Level 2 |  |  |
| NDUFS8 | Level 2 |  |  |
| GPD2 | Level 2 |  |  |
| NDUFS7 | Level 2 |  |  |
| NDUFV2 | Level 2 |  |  |
| NDUFA2 | Level 2 |  |  |
| NDUFB3 | Level 2 |  |  |
| NDUFB8 | Level 2 |  |  |
| NDUFS5 | Level 2 |  |  |
| NDUFB6 | Level 2 |  |  |
| NDUFS1 | Level 2 |  |  |
| NDUFB11 | Level 2 |  |  |
| NDUFA10 | Level 2 |  |  |
| NDUFV1 | Level 2 |  |  |
| CHRNB4 | Level 2 |  |  |
| PDE3A | Level 2 |  |  |
| VDR | Level 2 |  |  |
| PCDH18 | Level 2 |  |  |
| SVEP1 | Level 2 |  |  |
| PPARA | Level 2 |  |  |
| CMIP | Level 2 |  |  |
| COL4A1 | Level 2 |  |  |
| MECOM | Level 2 |  |  |
| SLC4A7 | Level 2 |  |  |
| FOXC1 | Level 2 |  |  |
| SH3PXD2A | Level 2 |  |  |
| ATP2B1 | Level 2 |  |  |
| ASXL1 | Level 2 |  |  |
| FGD5 | Level 2 |  |  |
| KIT | Level 2 |  |  |
| RUNX3 | Level 2 |  |  |
| DARS2 | Level 2 |  |  |
| ZNF652 | Level 2 |  |  |
| PDGFRB | Level 2 |  |  |
| DNMT3A | Level 2 |  |  |
| PIK3CG | Level 2 |  |  |
| NOS3 | Level 2 |  |  |
| NGF | Level 2 |  |  |
| ZBTB10 | Level 2 |  |  |
| ADORA2A | Level 2 |  |  |
| FBN2 | Level 2 |  |  |
| CCDC170 | Level 2 |  |  |
| PLCH1 | Level 2 |  |  |
| SIK1 | Level 2 |  |  |
| ABL1 | Level 2 |  |  |
| ERAP1 | Level 2 |  |  |
| BCR | Level 2 |  |  |
| ABCC8 | Level 2 |  |  |
| INPP5A | Level 2 |  |  |
| FRMD3 | Level 2 |  |  |
| TNF | Level 2 |  |  |
| DPP4 | Level 2 |  |  |
| COL4A2 | Level 2 |  |  |
| CDKAL1 | Level 2 |  |  |
| ESR1 | Level 2 |  |  |
| PDE10A | Level 2 |  |  |
| PURG | Level 2 |  |  |
| ZNF423 | Level 2 |  |  |
| PPARG | Level 2 |  |  |
| LSP1 | Level 2 |  |  |
| IRAK1BP1 | Level 2 |  |  |
| PKD2 | Level 2 |  |  |
| SOS2 | Level 2 |  |  |
| SEPTIN9 | Level 2 |  |  |
| COL4A4 | Level 2 |  |  |
| PHIP | Level 2 |  |  |
| WT1 | Level 2 |  |  |
| TH | Level 2 |  |  |
| PKHD1 | Level 2 |  |  |
| MAOA | Level 2 |  |  |
| HIPK2 | Level 2 |  |  |
| TTC21B | Level 2 |  |  |
| CHDH | Level 2 |  |  |
| CDKN2B | Level 2 |  |  |
| HDAC7 | Level 2 |  |  |
| TENM3 | Level 2 |  |  |
| EBF2 | Level 2 |  |  |
| MEF2C | Level 2 |  |  |
| MTX2 | Level 2 |  |  |
| MME | Level 2 |  |  |
| COL4A5 | Level 2 |  |  |
| PLG | Level 2 |  |  |
| PDGFRA | Level 2 |  |  |
| CSF1R | Level 2 |  |  |
| GRK6 | Level 2 |  |  |
| CCND1 | Level 2 |  |  |
| F12 | Level 2 |  |  |
| ANO7 | Level 2 |  |  |
| SLC25A37 | Level 2 |  |  |
| MEX3C | Level 2 |  |  |
| PITX2 | Level 2 |  |  |
| MEN1 | Level 2 |  |  |
| DIPK1A | Level 2 |  |  |
| RPL5 | Level 2 |  |  |
| PMS2 | Level 2 |  |  |
| YES1 | Level 2 |  |  |
| PPP2R2B | Level 2 |  |  |
| PKP4 | Level 2 |  |  |
| UFL1 | Level 2 |  |  |
| LRRC10B | Level 2 |  |  |
| TBX3 | Level 2 |  |  |
| HIVEP2 | Level 2 |  |  |
| ADH1B | Level 2 |  |  |
| ULK4 | Level 2 |  |  |
| NAA16 | Level 2 |  |  |
| PREX1 | Level 2 |  |  |
| PDE8A | Level 2 |  |  |
| NOP58 | Level 2 |  |  |
| MFHAS1 | Level 2 |  |  |
| CACNA1H | Level 2 |  |  |
| PTGER3 | Level 2 |  |  |
| NCOR2 | Level 2 |  |  |
| ADGRG6 | Level 2 |  |  |
| TBC1D19 | Level 2 |  |  |
| HIVEP3 | Level 2 |  |  |
| MAPK4 | Level 2 |  |  |
| MYO9B | Level 2 |  |  |
| JAG1 | Level 2 |  |  |
| PLCB1 | Level 2 |  |  |
| PRKAG2 | Level 2 |  |  |
| HOXC4 | Level 2 |  |  |
| THADA | Level 2 |  |  |
| NRIP1 | Level 2 |  |  |
| RBFOX1 | Level 2 |  |  |
| ZC3HC1 | Level 2 |  |  |
| CCND2 | Level 2 |  |  |
| RAD51B | Level 2 |  |  |
| GOSR2 | Level 2 |  |  |
| CNNM2 | Level 2 |  |  |
| KLHL21 | Level 2 |  |  |
| MEF2A | Level 2 |  |  |
| SOX6 | Level 2 |  |  |
| HSD17B12 | Level 2 |  |  |
| MYO6 | Level 2 |  |  |
| EFEMP1 | Level 2 |  |  |
| PAQR5 | Level 2 |  |  |
| HUWE1 | Level 2 |  |  |
| NFE2L2 | Level 2 |  |  |
| PTPRJ | Level 2 |  |  |
| SETBP1 | Level 2 |  |  |
| PRDM1 | Level 2 |  |  |
| PALM2AKAP2 | Level 2 |  |  |
| SBF2 | Level 2 |  |  |
| ANKRD55 | Level 2 |  |  |
| ITGB5 | Level 2 |  |  |
| CXADR | Level 2 |  |  |
| ADCY9 | Level 2 |  |  |
| SIPA1L1 | Level 2 |  |  |
| SHROOM3 | Level 2 |  |  |
| CA12 | Level 2 |  |  |
| GTF2I | Level 2 |  |  |
| CDKN2A | Level 2 |  |  |
| CDC25A | Level 2 |  |  |
| KEAP1 | Level 2 |  |  |
| MOCS2 | Level 2 |  |  |
| HSPA4 | Level 2 |  |  |
| TUBB1 | Level 2 |  |  |
| HAUS8 | Level 2 |  |  |
| GRIN1 | Level 2 |  |  |
| ZBTB34 | Level 2 |  |  |
| STXBP5 | Level 2 |  |  |
| SNX31 | Level 2 |  |  |
| ESR2 | Level 2 |  |  |
| VIM | Level 2 |  |  |
| FAM13A | Level 2 |  |  |
| ARL15 | Level 2 |  |  |
| NFATC2 | Level 2 |  |  |
| MYH9 | Level 2 |  |  |
| TUBB6 | Level 2 |  |  |
| GRIN2A | Level 2 |  |  |
| TUBB3 | Level 2 |  |  |
| TUBB4A | Level 2 |  |  |
| TUBB2A | Level 2 |  |  |
| TUBB4B | Level 2 |  |  |
| TUBB8 | Level 2 |  |  |
| TUBB | Level 2 |  |  |
| GRIN3B | Level 2 |  |  |
| GRIN2D | Level 2 |  |  |
| TUBB2B | Level 2 |  |  |
| HFE | Level 2 |  |  |
| ARHGAP15 | Level 2 |  |  |
| LMO1 | Level 2 |  |  |
| USP28 | Level 2 |  |  |
| ARHGEF18 | Level 2 |  |  |
| LEF1 | Level 2 |  |  |
| FLT1 | Level 2 |  |  |
| GRIA2 | Level 2 |  |  |
| HSF2BP | Level 2 |  |  |
| ZBTB46 | Level 2 |  |  |
| TERT | Level 2 |  |  |
| SYT1 | Level 2 |  |  |
| GJB2 | Level 2 |  |  |
| INF2 | Level 2 |  |  |
| ZFP36L1 | Level 2 |  |  |
| F10 | Level 2 |  |  |
| CFDP1 | Level 2 |  |  |
| BCL2L11 | Level 2 |  |  |
| MGAM | Level 2 |  |  |
| GRIA1 | Level 2 |  |  |
| GRB14 | Level 2 |  |  |
| NT5C2 | Level 2 |  |  |
| GRIK1 | Level 2 |  |  |
| GRIA3 | Level 2 |  |  |
| GRIK2 | Level 2 |  |  |
| GRIK5 | Level 2 |  |  |
| WNT3A | Level 2 |  |  |
| DGKH | Level 2 |  |  |
| KDM2B | Level 2 |  |  |
| NIFK | Level 2 |  |  |
| MKLN1 | Level 2 |  |  |
| NHLRC2 | Level 2 |  |  |
| KAT2B | Level 2 |  |  |
| ID2 | Level 2 |  |  |
| USP8 | Level 2 |  |  |
| FOSL2 | Level 2 |  |  |
| ATG7 | Level 2 |  |  |
| SPTBN1 | Level 2 |  |  |
| DNAJC10 | Level 2 |  |  |
| ARHGAP29 | Level 2 |  |  |
| YWHAE | Level 2 |  |  |
| ZSWIM6 | Level 2 |  |  |
| PLXNB2 | Level 2 |  |  |
| TET2 | Level 2 |  |  |
| RALGPS1 | Level 2 |  |  |
| TMEFF2 | Level 2 |  |  |
| PGPEP1 | Level 2 |  |  |
| GTF2B | Level 2 |  |  |
| PIK3R1 | Level 2 |  |  |
| DCAKD | Level 2 |  |  |
| SOX3 | Level 2 |  |  |
| ZNF268 | Level 2 |  |  |
| NFAT5 | Level 2 |  |  |
| GPATCH2 | Level 2 |  |  |
| ZNF318 | Level 2 |  |  |
| CDC16 | Level 2 |  |  |
| KLHL24 | Level 2 |  |  |
| TRPC5 | Level 2 |  |  |
| CREB1 | Level 2 |  |  |
| PPP2R2A | Level 2 |  |  |
| NR2F2 | Level 2 |  |  |
| MAD1L1 | Level 2 |  |  |
| SLC2A4 | Level 2 |  |  |
| LRRC7 | Level 2 |  |  |
| DCP2 | Level 2 |  |  |
| CWF19L2 | Level 2 |  |  |
| CRYAA | Level 2 |  |  |
| PABPC4 | Level 2 |  |  |
| FBF1 | Level 2 |  |  |
| TCEA2 | Level 2 |  |  |
| DOCK3 | Level 2 |  |  |
| SIPA1 | Level 2 |  |  |
| ZNF443 | Level 2 |  |  |
| MON2 | Level 2 |  |  |
| MDM4 | Level 2 |  |  |
| FBRSL1 | Level 2 |  |  |
| MEF2D | Level 2 |  |  |
| PRKD3 | Level 2 |  |  |
| SPRY4 | Level 2 |  |  |
| COL4A3 | Level 2 |  |  |
| GLI2 | Level 2 |  |  |
| IPO9 | Level 2 |  |  |
| MORC3 | Level 2 |  |  |
| STN1 | Level 2 |  |  |
| DLG4 | Level 2 |  |  |
| DNAJC1 | Level 2 |  |  |
| IRF6 | Level 2 |  |  |
| KIF23 | Level 2 |  |  |
| RNF126 | Level 2 |  |  |
| ANKS1A | Level 2 |  |  |
| EHD4 | Level 2 |  |  |
| MARK3 | Level 2 |  |  |
| SPI1 | Level 2 |  |  |
| TNRC6A | Level 2 |  |  |
| PEPD | Level 2 |  |  |
| PRDM16 | Level 2 |  |  |
| S1PR2 | Level 2 |  |  |
| EMSY | Level 2 |  |  |
| FURIN | Level 2 |  |  |
| CSK | Level 2 |  |  |
| MCU | Level 2 |  |  |
| TSN | Level 2 |  |  |
| MS4A4A | Level 2 |  |  |
| TPPP | Level 2 |  |  |
| GGCX | Level 2 |  |  |
| NUMB | Level 2 |  |  |
| USP34 | Level 2 |  |  |
| AQP1 | Level 2 |  |  |
| CCDC88C | Level 2 |  |  |
| VEGFA | Level 2 |  |  |
| MTUS2 | Level 2 |  |  |
| RRP1B | Level 2 |  |  |
| ZNF407 | Level 2 |  |  |
| ERI1 | Level 2 |  |  |
| HMGA1 | Level 2 |  |  |
| NOTCH3 | Level 2 |  |  |
| CHMP1A | Level 2 |  |  |
| CEP170 | Level 2 |  |  |
| MADD | Level 2 |  |  |
| DPYSL2 | Level 2 |  |  |
| DNMT1 | Level 2 |  |  |
| FAM193A | Level 2 |  |  |
| IMMP2L | Level 2 |  |  |
| SLC1A3 | Level 2 |  |  |
| HSPA12A | Level 2 |  |  |
| MLF1 | Level 2 |  |  |
| HOXA10 | Level 2 |  |  |
| IGF2BP2 | Level 2 |  |  |
| FGFR2 | Level 2 |  |  |
| ARID3C | Level 2 |  |  |
| TARS1 | Level 2 |  |  |
| H2BC4 | Level 2 |  |  |
| CENPV | Level 2 |  |  |
| BLK | Level 2 |  |  |
| MAPKAP1 | Level 2 |  |  |
| TP53INP1 | Level 2 |  |  |
| SDCCAG8 | Level 2 |  |  |
| CLINT1 | Level 2 |  |  |
| TRIM65 | Level 2 |  |  |
| MARCHF2 | Level 2 |  |  |
| EEFSEC | Level 2 |  |  |
| HOXB7 | Level 2 |  |  |
| NOTCH1 | Level 2 |  |  |
| PIP4K2B | Level 2 |  |  |
| EDA2R | Level 2 |  |  |
| RERE | Level 2 |  |  |
| PLCG1 | Level 2 |  |  |
| BMPR1B | Level 2 |  |  |
| MYNN | Level 2 |  |  |
| AKR1B1 | Level 2 |  |  |
| NMT1 | Level 2 |  |  |
| RIN3 | Level 2 |  |  |
| ATXN2 | Level 2 |  |  |
| SMAD4 | Level 2 |  |  |
| ZZEF1 | Level 2 |  |  |
| CLDN7 | Level 2 |  |  |
| PKN2 | Level 2 |  |  |
| PIK3C2B | Level 2 |  |  |
| ZHX3 | Level 2 |  |  |
| MTHFR | Level 2 |  |  |
| PNO1 | Level 2 |  |  |
| MOV10 | Level 2 |  |  |
| ZDHHC18 | Level 2 |  |  |
| FAT1 | Level 2 |  |  |
| NUF2 | Level 2 |  |  |
| CCDC90B | Level 2 |  |  |
| FBXO38 | Level 2 |  |  |
| NEK10 | Level 2 |  |  |
| GEMIN2 | Level 2 |  |  |
| SEC23A | Level 2 |  |  |
| GMDS | Level 2 |  |  |
| CHP1 | Level 2 |  |  |
| TRAF3IP3 | Level 2 |  |  |
| METTL15 | Level 2 |  |  |
| SIK2 | Level 2 |  |  |
| TASOR | Level 2 |  |  |
| AKR1B15 | Level 2 |  |  |
| CLCNKA | Level 2 |  |  |
| SHISA4 | Level 2 |  |  |
| THAP3 | Level 2 |  |  |
| NOC3L | Level 2 |  |  |
| VARS1 | Level 2 |  |  |
| CDC27 | Level 2 |  |  |
| GFM1 | Level 2 |  |  |
| ZFP91 | Level 2 |  |  |
| RPL4 | Level 2 |  |  |
| ARHGEF16 | Level 2 |  |  |
| ILRUN | Level 2 |  |  |
| PRKAA2 | Level 2 |  |  |
| PODXL | Level 2 |  |  |
| BCAS3 | Level 2 |  |  |
| BNIP3L | Level 2 |  |  |
| TRIM47 | Level 2 |  |  |
| WRN | Level 2 |  |  |
| BMPR1A | Level 2 |  |  |
| ZNF100 | Level 2 |  |  |
| KDM4B | Level 2 |  |  |
| HIBADH | Level 2 |  |  |
| RHOC | Level 2 |  |  |
| SIDT2 | Level 2 |  |  |
| CYTH1 | Level 2 |  |  |
| MRPS12 | Level 2 |  |  |
| KSR2 | Level 2 |  |  |
| PHF6 | Level 2 |  |  |
| RBM23 | Level 2 |  |  |
| DCP1A | Level 2 |  |  |
| SMG6 | Level 2 |  |  |
| OPRL1 | Level 2 |  |  |
| CDKN1B | Level 2 |  |  |
| CEBPZ | Level 2 |  |  |
| ICA1L | Level 2 |  |  |
| SNAPC5 | Level 2 |  |  |
| MCC | Level 2 |  |  |
| SMAD1 | Level 2 |  |  |
| SPRYD7 | Level 2 |  |  |
| SGK1 | Level 2 |  |  |
| NSUN6 | Level 2 |  |  |
| WDR11 | Level 2 |  |  |
| EPB41 | Level 2 |  |  |
| PAFAH1B2 | Level 2 |  |  |
| GFER | Level 2 |  |  |
| MAST2 | Level 2 |  |  |
| ALG9 | Level 2 |  |  |
| INO80 | Level 2 |  |  |
| FOLH1 | Level 2 |  |  |
| IL17RB | Level 2 |  |  |
| CARF | Level 2 |  |  |
| PPP3R1 | Level 2 |  |  |
| VKORC1 | Level 2 |  |  |
| AOC1 | Level 2 |  |  |
| CISD2 | Level 2 |  |  |
| CKB | Level 2 |  |  |
| SRRT | Level 2 |  |  |
| IL17A | Level 2 |  |  |
| FKBP1A | Level 2 |  |  |
| HEXIM1 | Level 2 |  |  |
| ARID5B | Level 2 |  |  |
| HOXB5 | Level 2 |  |  |
| IL6R | Level 2 |  |  |
| SLX4IP | Level 2 |  |  |
| ARF1 | Level 2 |  |  |
| PIM3 | Level 2 |  |  |
| H4C3 | Level 2 |  |  |
| NR2C2 | Level 2 |  |  |
| CDK6 | Level 2 |  |  |
| PTEN | Level 2 |  |  |
| MTNR1A | Level 2 |  |  |
| OPRM1 | Level 2 |  |  |
| SENP2 | Level 2 |  |  |
| MPO | Level 2 |  |  |
| MKRN2 | Level 2 |  |  |
| ZNF875 | Level 2 |  |  |
| KCNRG | Level 2 |  |  |
| ZAP70 | Level 2 |  |  |
| ZNF738 | Level 2 |  |  |
| RNF34 | Level 2 |  |  |
| MC4R | Level 2 |  |  |
| ERAP2 | Level 2 |  |  |
| BRD2 | Level 2 |  |  |
| PHETA1 | Level 2 |  |  |
| SUN1 | Level 2 |  |  |
| UBE2D3 | Level 2 |  |  |
| LXN | Level 2 |  |  |
| GET4 | Level 2 |  |  |
| CTDNEP1 | Level 2 |  |  |
| LOX | Level 2 |  |  |
| PCM1 | Level 2 |  |  |
| SNRPC | Level 2 |  |  |
| RPAP3 | Level 2 |  |  |
| EXD3 | Level 2 |  |  |
| PINX1 | Level 2 |  |  |
| ZNF646 | Level 2 |  |  |
| RPS16 | Level 2 |  |  |
| CYB5D1 | Level 2 |  |  |
| BRD4 | Level 2 |  |  |
| CHD3 | Level 2 |  |  |
| PTK2 | Level 2 |  |  |
| AXIN1 | Level 2 |  |  |
| HNRNPM | Level 2 |  |  |
| HLA-DPB1 | Level 2 |  |  |
| XBP1 | Level 2 |  |  |
| KLF3 | Level 2 |  |  |
| ACP2 | Level 2 |  |  |
| PGM2 | Level 2 |  |  |
| GATA2 | Level 2 |  |  |
| NAV1 | Level 2 |  |  |
| PPL | Level 2 |  |  |
| UTP25 | Level 2 |  |  |
| GRHL2 | Level 2 |  |  |
| DCAF16 | Level 2 |  |  |
| MCRS1 | Level 2 |  |  |
| TRIP6 | Level 2 |  |  |
| BRSK2 | Level 2 |  |  |
| HSPH1 | Level 2 |  |  |
| ADAR | Level 2 |  |  |
| POM121C | Level 2 |  |  |
| TOP1 | Level 2 |  |  |
| XPO1 | Level 2 |  |  |
| GAB1 | Level 2 |  |  |
| ZWILCH | Level 2 |  |  |
| ANAPC5 | Level 2 |  |  |
| POC1B | Level 2 |  |  |
| KDM6B | Level 2 |  |  |
| IL6ST | Level 2 |  |  |
| ZNF708 | Level 2 |  |  |
| AHDC1 | Level 2 |  |  |
| DNM1L | Level 2 |  |  |
| ABCF3 | Level 2 |  |  |
| H4C8 | Level 2 |  |  |
| DVL2 | Level 2 |  |  |
| MRPL38 | Level 2 |  |  |
| FAF1 | Level 2 |  |  |
| MAP3K5 | Level 2 |  |  |
| BRD3 | Level 2 |  |  |
| MUC13 | Level 2 |  |  |
| BRDT | Level 2 |  |  |
| TNNT3 | Level 2 |  |  |
| ZNF689 | Level 2 |  |  |
| ALG3 | Level 2 |  |  |
| MFF | Level 2 |  |  |
| GIT2 | Level 2 |  |  |
| CD80 | Level 2 |  |  |
| ARID1A | Level 2 |  |  |
| GEM | Level 2 |  |  |
| EFR3B | Level 2 |  |  |
| TRPA1 | Level 2 |  |  |
| NEDD9 | Level 2 |  |  |
| IGF2BP1 | Level 2 |  |  |
| ARPC3 | Level 2 |  |  |
| HLA-B | Level 2 |  |  |
| RBMXL1 | Level 2 |  |  |
| COPS4 | Level 2 |  |  |
| MAP2K1 | Level 2 |  |  |
| HOXB9 | Level 2 |  |  |
| DDB2 | Level 2 |  |  |
| U2AF1 | Level 2 |  |  |
| RAD51C | Level 2 |  |  |
| TBC1D1 | Level 2 |  |  |
| BCKDK | Level 2 |  |  |
| SNCAIP | Level 2 |  |  |
| ZFP64 | Level 2 |  |  |
| CAPZA1 | Level 2 |  |  |
| ACTR1B | Level 2 |  |  |
| MFN2 | Level 2 |  |  |
| VGLL2 | Level 2 |  |  |
| SRSF4 | Level 2 |  |  |
| SLC22A3 | Level 2 |  |  |
| FAM171B | Level 2 |  |  |
| RNF123 | Level 2 |  |  |
| ZNF140 | Level 2 |  |  |
| TMEM199 | Level 2 |  |  |
| SEC31A | Level 2 |  |  |
| IGF2R | Level 2 |  |  |
| WASF2 | Level 2 |  |  |
| SEPTIN4 | Level 2 |  |  |
| PPP2R1B | Level 2 |  |  |
| UBE2Z | Level 2 |  |  |
| ZNF84 | Level 2 |  |  |
| PLCB4 | Level 2 |  |  |
| DDX47 | Level 2 |  |  |
| IRS1 | Level 2 |  |  |
| COG2 | Level 2 |  |  |
| KCTD1 | Level 2 |  |  |
| PRMT5 | Level 2 |  |  |
| H1-6 | Level 2 |  |  |
| NCF1 | Level 2 |  |  |
| OAS2 | Level 2 |  |  |
| PLCB3 | Level 2 |  |  |
| HEXIM2 | Level 2 |  |  |
| TCHP | Level 2 |  |  |
| ACAP1 | Level 2 |  |  |
| CCNT2 | Level 2 |  |  |
| TMEM165 | Level 2 |  |  |
| SIGMAR1 | Level 2 |  |  |
| EIF4G1 | Level 2 |  |  |
| SNF8 | Level 2 |  |  |
| ECHDC1 | Level 2 |  |  |
| HNF4A | Level 2 |  |  |
| C7ORF50 | Level 2 |  |  |
| SLC22A4 | Level 2 |  |  |
| INTS12 | Level 2 |  |  |
| H2AC6 | Level 2 |  |  |
| LARP4 | Level 2 |  |  |
| HDLBP | Level 2 |  |  |
| OBSCN | Level 2 |  |  |
| DCAF1 | Level 2 |  |  |
| BAG4 | Level 2 |  |  |
| TRIM13 | Level 2 |  |  |
| WDR12 | Level 2 |  |  |
| ZNF107 | Level 2 |  |  |
| TBR1 | Level 2 |  |  |
| KLF2 | Level 2 |  |  |
| NUDT16L1 | Level 2 |  |  |
| MCM8 | Level 2 |  |  |
| PIK3R3 | Level 2 |  |  |
| PLEKHA1 | Level 2 |  |  |
| HOXA9 | Level 2 |  |  |
| LANCL1 | Level 2 |  |  |
| SIK3 | Level 2 |  |  |
| PDE4A | Level 2 |  |  |
| ALDH2 | Level 2 |  |  |
| RMDN3 | Level 2 |  |  |
| GPANK1 | Level 2 |  |  |
| KAT8 | Level 2 |  |  |
| PNMA2 | Level 2 |  |  |
| PRKCSH | Level 2 |  |  |
| ZFP36L2 | Level 2 |  |  |
| FDXACB1 | Level 2 |  |  |
| CAST | Level 2 |  |  |
| PDE4D | Level 2 |  |  |
| TNKS | Level 2 |  |  |
| NQO1 | Level 2 |  |  |
| PPP2R3A | Level 2 |  |  |
| TRIM73 | Level 2 |  |  |
| ULK3 | Level 2 |  |  |
| TSHZ2 | Level 2 |  |  |
| PSMC3 | Level 2 |  |  |
| PDE3B | Level 2 |  |  |
| GOLIM4 | Level 2 |  |  |
| FANCA | Level 2 |  |  |
| FST | Level 2 |  |  |
| ATE1 | Level 2 |  |  |
| RRM2B | Level 2 |  |  |
| BCL7A | Level 2 |  |  |
| PBX3 | Level 2 |  |  |
| LPIN3 | Level 2 |  |  |
| DNAAF5 | Level 2 |  |  |
| PLN | Level 2 |  |  |
| NUDC | Level 2 |  |  |
| ARL14EP | Level 2 |  |  |
| NUCB2 | Level 2 |  |  |
| PPP1R7 | Level 2 |  |  |
| PRELID1 | Level 2 |  |  |
| RNF146 | Level 2 |  |  |
| PARP1 | Level 2 |  |  |
| PDK1 | Level 2 |  |  |
| STAG1 | Level 2 |  |  |
| SRCIN1 | Level 2 |  |  |
| STX4 | Level 2 |  |  |
| LMNB2 | Level 2 |  |  |
| IL37 | Level 2 |  |  |
| DNAJB8 | Level 2 |  |  |
| TNIP2 | Level 2 |  |  |
| RET | Level 2 |  |  |
| ANKRD13A | Level 2 |  |  |
| POLDIP2 | Level 2 |  |  |
| DNAJC11 | Level 2 |  |  |
| PSMB3 | Level 2 |  |  |
| MICU2 | Level 2 |  |  |
| MTAP | Level 2 |  |  |
| EIF4B | Level 2 |  |  |
| ARHGAP10 | Level 2 |  |  |
| MTMR4 | Level 2 |  |  |
| CNR2 | Level 2 |  |  |
| ZNF527 | Level 2 |  |  |
| MAP4 | Level 2 |  |  |
| NLRC3 | Level 2 |  |  |
| KCNN2 | Level 2 |  |  |
| THG1L | Level 2 |  |  |
| PLEKHA7 | Level 2 |  |  |
| ZNF589 | Level 2 |  |  |
| MRPL15 | Level 2 |  |  |
| MAPK11 | Level 2 |  |  |
| MINDY4 | Level 2 |  |  |
| RBM33 | Level 2 |  |  |
| FGFR1 | Level 2 |  |  |
| ZCCHC10 | Level 2 |  |  |
| IRF1 | Level 2 |  |  |
| ADCY3 | Level 2 |  |  |
| ADAMTS12 | Level 2 |  |  |
| FLT4 | Level 2 |  |  |
| SRD5A3 | Level 2 |  |  |
| CTSD | Level 2 |  |  |
| HAUS4 | Level 2 |  |  |
| PPP4R3A | Level 2 |  |  |
| SERTAD4 | Level 2 |  |  |
| MAPK3 | Level 2 |  |  |
| ITGA2 | Level 2 |  |  |
| MTRF1 | Level 2 |  |  |
| PDCD10 | Level 2 |  |  |
| ZNF793 | Level 2 |  |  |
| BAG6 | Level 2 |  |  |
| TKFC | Level 2 |  |  |
| HLA-C | Level 2 |  |  |
| C11ORF58 | Level 2 |  |  |
| RAB24 | Level 2 |  |  |
| RHEB | Level 2 |  |  |
| ALKBH3 | Level 2 |  |  |
| LAMB2 | Level 2 |  |  |
| RASA3 | Level 2 |  |  |
| PTP4A3 | Level 2 |  |  |
| RPP25L | Level 2 |  |  |
| HIP1 | Level 2 |  |  |
| WEE1 | Level 2 |  |  |
| PARP2 | Level 2 |  |  |
| TMEM97 | Level 2 |  |  |
| SLC25A4 | Level 2 |  |  |
| BTN2A1 | Level 2 |  |  |
| PRNP | Level 2 |  |  |
| BRAF | Level 2 |  |  |
| PDXK | Level 2 |  |  |
| KYAT3 | Level 2 |  |  |
| FARP2 | Level 2 |  |  |
| PPP1R12A | Level 2 |  |  |
| HOXA7 | Level 2 |  |  |
| UHRF1 | Level 2 |  |  |
| ZW10 | Level 2 |  |  |
| LMBR1 | Level 2 |  |  |
| NSD3 | Level 2 |  |  |
| KDF1 | Level 2 |  |  |
| DPYS | Level 2 |  |  |
| RAF1 | Level 2 |  |  |
| LIN7B | Level 2 |  |  |
| TCF25 | Level 2 |  |  |
| MTHFD1L | Level 2 |  |  |
| TKT | Level 2 |  |  |
| PDE2A | Level 2 |  |  |
| MOB2 | Level 2 |  |  |
| SOAT2 | Level 2 |  |  |
| ATP1A2 | Level 2 |  |  |
| ATP1A1 | Level 2 |  |  |
| LPXN | Level 2 |  |  |
| RCC1L | Level 2 |  |  |
| SLC29A1 | Level 2 |  |  |
| APEH | Level 2 |  |  |
| FLT3 | Level 2 |  |  |
| PDK2 | Level 2 |  |  |
| CRYBA2 | Level 2 |  |  |
| IMPDH2 | Level 2 |  |  |
| PDE6H | Level 2 |  |  |
| ATP1A3 | Level 2 |  |  |
| IMPDH1 | Level 2 |  |  |
| PDK3 | Level 2 |  |  |
| PDE9A | Level 2 |  |  |
| ATP1A4 | Level 2 |  |  |
| VASN | Level 2 |  |  |
| LMAN2 | Level 2 |  |  |
| CFL1 | Level 2 |  |  |
| SMARCD1 | Level 2 |  |  |
| CLIC1 | Level 2 |  |  |
| IRF3 | Level 2 |  |  |
| PHF8 | Level 2 |  |  |
| ABCB8 | Level 2 |  |  |
| GLYR1 | Level 2 |  |  |
| PRRC2A | Level 2 |  |  |
| RGCC | Level 2 |  |  |
| KIF2A | Level 2 |  |  |
| DDAH2 | Level 2 |  |  |
| ZNF76 | Level 2 |  |  |
| SEPTIN2 | Level 2 |  |  |
| ADA | Level 2 |  |  |
| MAP1LC3B | Level 2 |  |  |
| MSH5 | Level 2 |  |  |
| CCNB1 | Level 2 |  |  |
| INA | Level 2 |  |  |
| APEX2 | Level 2 |  |  |
| MAPK10 | Level 2 |  |  |
| NCAPG | Level 2 |  |  |
| PPM1H | Level 2 |  |  |
| LIMA1 | Level 2 |  |  |
| MTA3 | Level 2 |  |  |
| COA8 | Level 2 |  |  |
| CRYAB | Level 2 |  |  |
| CBS | Level 2 |  |  |
| RNF40 | Level 2 |  |  |
| CCDC186 | Level 2 |  |  |
| HOXB6 | Level 2 |  |  |
| SNRNP70 | Level 2 |  |  |
| TSC2 | Level 2 |  |  |
| CTNNB1 | Level 2 |  |  |
| PCGF2 | Level 2 |  |  |
| TLR1 | Level 2 |  |  |
| VWA8 | Level 2 |  |  |
| TRIM38 | Level 2 |  |  |
| KLHDC10 | Level 2 |  |  |
| IL31 | Level 2 |  |  |
| BSN | Level 2 |  |  |
| NUDT2 | Level 2 |  |  |
| TNFAIP1 | Level 2 |  |  |
| DNAH8 | Level 2 |  |  |
| TBKBP1 | Level 2 |  |  |
| TNKS1BP1 | Level 2 |  |  |
| P4HA2 | Level 2 |  |  |
| ZBTB43 | Level 2 |  |  |
| CHMP4C | Level 2 |  |  |
| KANK2 | Level 2 |  |  |
| FAM167A | Level 2 |  |  |
| ZNF668 | Level 2 |  |  |
| BCAR1 | Level 2 |  |  |
| CORO7 | Level 2 |  |  |
| TOMM40 | Level 2 |  |  |
| C1ORF35 | Level 2 |  |  |
| CHRD | Level 2 |  |  |
| ITGAV | Level 2 |  |  |
| ERP29 | Level 2 |  |  |
| TEX14 | Level 2 |  |  |
| TBL3 | Level 2 |  |  |
| FKBP2 | Level 2 |  |  |
| TM9SF4 | Level 2 |  |  |
| BAG3 | Level 2 |  |  |
| KLF8 | Level 2 |  |  |
| PLCG2 | Level 2 |  |  |
| SYTL1 | Level 2 |  |  |
| SFN | Level 2 |  |  |
| DVL3 | Level 2 |  |  |
| CNTFR | Level 2 |  |  |
| NSF | Level 2 |  |  |
| SCAMP1 | Level 2 |  |  |
| TGIF1 | Level 2 |  |  |
| TCERG1 | Level 2 |  |  |
| FBXO31 | Level 2 |  |  |
| SERINC3 | Level 2 |  |  |
| KIF3B | Level 2 |  |  |
| TENT4B | Level 2 |  |  |
| PAWR | Level 2 |  |  |
| SCAMP2 | Level 2 |  |  |
| TLR6 | Level 2 |  |  |
| EIF2B5 | Level 2 |  |  |
| H2BC10 | Level 2 |  |  |
| ZNF664 | Level 2 |  |  |
| ITPR2 | Level 2 |  |  |
| NCOR1 | Level 2 |  |  |
| CALCOCO2 | Level 2 |  |  |
| TFAM | Level 2 |  |  |
| RMND1 | Level 2 |  |  |
| SLK | Level 2 |  |  |
| KAT5 | Level 2 |  |  |
| TRAP1 | Level 2 |  |  |
| RUVBL1 | Level 2 |  |  |
| FAM107A | Level 2 |  |  |
| AJUBA | Level 2 |  |  |
| PNN | Level 2 |  |  |
| ANAPC7 | Level 2 |  |  |
| PRMT1 | Level 2 |  |  |
| MRM2 | Level 2 |  |  |
| MIA2 | Level 2 |  |  |
| TOPBP1 | Level 2 |  |  |
| MACF1 | Level 2 |  |  |
| PRKAR1B | Level 2 |  |  |
| HADH | Level 2 |  |  |
| ZNF598 | Level 2 |  |  |
| AIG1 | Level 2 |  |  |
| IGFBP6 | Level 2 |  |  |
| FNBP4 | Level 2 |  |  |
| INO80E | Level 2 |  |  |
| COMMD7 | Level 2 |  |  |
| SCD5 | Level 2 |  |  |
| MAPK12 | Level 2 |  |  |
| DPH1 | Level 2 |  |  |
| LYPLA1 | Level 2 |  |  |
| DIP2B | Level 2 |  |  |
| IP6K1 | Level 2 |  |  |
| SH3BP2 | Level 2 |  |  |
| IPO11 | Level 2 |  |  |
| ACTR8 | Level 2 |  |  |
| TRMT6 | Level 2 |  |  |
| ELOVL1 | Level 2 |  |  |
| TTC19 | Level 2 |  |  |
| QPCT | Level 2 |  |  |
| TAOK2 | Level 2 |  |  |
| SENP1 | Level 2 |  |  |
| NUP160 | Level 2 |  |  |
| MYO1C | Level 2 |  |  |
| NAA25 | Level 2 |  |  |
| CRK | Level 2 |  |  |
| CYP2U1 | Level 2 |  |  |
| EMILIN3 | Level 2 |  |  |
| GAS2 | Level 2 |  |  |
| DIMT1 | Level 2 |  |  |
| LRRC40 | Level 2 |  |  |
| NCOA1 | Level 2 |  |  |
| SMARCA5 | Level 2 |  |  |
| ENOSF1 | Level 2 |  |  |
| ZNF785 | Level 2 |  |  |
| GUK1 | Level 2 |  |  |
| TPD52L2 | Level 2 |  |  |
| BAG5 | Level 2 |  |  |
| ZNF22 | Level 2 |  |  |
| CDSN | Level 2 |  |  |
| PLA2G4B | Level 2 |  |  |
| SSRP1 | Level 2 |  |  |
| MSRA | Level 2 |  |  |
| SYT7 | Level 2 |  |  |
| PPIC | Level 2 |  |  |
| RRM2 | Level 2 |  |  |
| NETO1 | Level 2 |  |  |
| MTCH2 | Level 2 |  |  |
| MGLL | Level 2 |  |  |
| MCMBP | Level 2 |  |  |
| H1-7 | Level 2 |  |  |
| NTHL1 | Level 2 |  |  |
| RAB3D | Level 2 |  |  |
| H2BC3 | Level 2 |  |  |
| PPFIA1 | Level 2 |  |  |
| SPIN3 | Level 2 |  |  |
| RBM6 | Level 2 |  |  |
| SARS1 | Level 2 |  |  |
| RAC1 | Bridging |  |  |
| HNRNPL | Bridging |  |  |
| MAP3K7 | Bridging |  |  |
| SMURF1 | Bridging |  |  |
| SMAD6 | Bridging |  |  |
| TRAF3 | Bridging |  |  |
| PLEKHA4 | Bridging |  |  |
| SQSTM1 | Bridging |  |  |
| WDYHV1 | Bridging |  |  |
| SMAD3 | Bridging |  |  |
| TRIM28 | Bridging |  |  |
| IKBKG | Bridging |  |  |
| BTK | Bridging |  |  |
| MAP4K4 | Bridging |  |  |
| TNFRSF1A | Bridging |  |  |
| IRAK1 | Bridging |  |  |
| TRAF6 | Bridging |  |  |
| TRAF2 | Bridging |  |  |
| NGFR | Bridging |  |  |
| HDAC6 | Bridging |  |  |
| TLR4 | Bridging |  |  |
| RNF4 | Bridging |  |  |
| ATG5 | Bridging |  |  |
| TLR9 | Bridging |  |  |
| SIAH1 | Bridging |  |  |
| AKT1 | Bridging |  |  |
| TXN | Bridging |  |  |
| POLR1C | Bridging |  |  |
| MDM2 | Bridging |  |  |
| MAPK14 | Bridging |  |  |
| TSG101 | Bridging |  |  |
| PRDX1 | Bridging |  |  |
| IL1B | Bridging |  |  |
| NFKBIA | Bridging |  |  |
| IKBKE | Bridging |  |  |
| CYLD | Bridging |  |  |
| IKBKB | Bridging |  |  |
| IRAK3 | Bridging |  |  |
| INS | Bridging |  |  |
| CYTH2 | Bridging |  |  |
| IRF7 | Bridging |  |  |
| SPOP | Bridging |  |  |
| SIAH2 | Bridging |  |  |
| PTK2B | Bridging |  |  |
| SARM1 | Bridging |  |  |
| GLOD4 | Bridging |  |  |
| NOD2 | Bridging |  |  |
| CD93 | Bridging |  |  |
| PFKL | Bridging |  |  |
| FLII | Bridging |  |  |
| LRRFIP2 | Bridging |  |  |
| SYK | Bridging |  |  |
| CBLB | Bridging |  |  |
| CISH | Bridging |  |  |
| IL1R1 | Bridging |  |  |
| USP7 | Bridging |  |  |
| PIK3AP1 | Bridging |  |  |
| CD14 | Bridging |  |  |
| TNIP1 | Bridging |  |  |
| FADD | Bridging |  |  |
| TBK1 | Bridging |  |  |
| LRRFIP1 | Bridging |  |  |
| MAP3K1 | Bridging |  |  |
| SASH1 | Bridging |  |  |
| MBIP | Bridging |  |  |
| CAMLG | Bridging |  |  |
| IRAK2 | Bridging |  |  |
| STAP2 | Bridging |  |  |
| CARD9 | Bridging |  |  |
| IRF4 | Bridging |  |  |
| IRF5 | Bridging |  |  |
| TLR2 | Bridging |  |  |
| IRAK4 | Bridging |  |  |
| PELI1 | Bridging |  |  |
| DOCK8 | Bridging |  |  |
| CASP1 | Bridging |  |  |
| MAL | Bridging |  |  |
| RIPK2 | Bridging |  |  |
| BST2 | Bridging |  |  |
| ARMCX6 | Bridging |  |  |
| TLR8 | Bridging |  |  |
| UBAP1 | Bridging |  |  |
| IL1RAP | Bridging |  |  |
| TIRAP | Bridging |  |  |
| TLR3 | Bridging |  |  |
| ZNF35 | Bridging |  |  |
| TNFRSF13B | Bridging |  |  |
| TLR7 | Bridging |  |  |
| BANK1 | Bridging |  |  |
| TLR10 | Bridging |  |  |
| PELI2 | Bridging |  |  |
| IL1RL2 | Bridging |  |  |
| TLR5 | Bridging |  |  |
| AGER | Bridging |  |  |
| RNF152 | Bridging |  |  |
| SKIL | Level 2 | Pattaro, 2016 | 26831199 |
| ETV5 | Level 2 |  |  |
| A1CF | Level 2 |  |  |
| KCNQ1 | Level 2 |  |  |
| NFATC1 | Level 2 |  |  |
| SDCCAG8 | Level 2 |  |  |
| LRP2 | Level 2 |  |  |
| WNT7A | Level 2 |  |  |
| NFKB1 | Level 2 |  |  |
| TSPAN9 | Level 2 |  |  |
| PTPRO | Level 2 |  |  |
| SIPA1L3 | Level 2 |  |  |
| SMAD3 | Bridging |  |  |
| MAP3K7 | Bridging |  |  |
| RNF4 | Bridging |  |  |
| SASH1 | Bridging |  |  |
| CISH | Bridging |  |  |
| INS | Bridging |  |  |
| TRAF6 | Bridging |  |  |
| MAPK14 | Bridging |  |  |
| HNRNPL | Bridging |  |  |
| IKBKG | Bridging |  |  |
| LRRFIP2 | Bridging |  |  |
| RAC1 | Bridging |  |  |
| PIK3AP1 | Bridging |  |  |
| IKBKB | Bridging |  |  |
| TXN | Bridging |  |  |
| TNIP1 | Bridging |  |  |
| FADD | Bridging |  |  |
| TLR2 | Bridging |  |  |
| RIPK2 | Bridging |  |  |
| IRAK4 | Bridging |  |  |
| TBK1 | Bridging |  |  |
| TLR3 | Bridging |  |  |
| NOD2 | Bridging |  |  |
| NFKBIA | Bridging |  |  |
| IKBKE | Bridging |  |  |
| PRDX1 | Bridging |  |  |
| IL1R1 | Bridging |  |  |
| CD14 | Bridging |  |  |
| MAP3K1 | Bridging |  |  |
| TLR5 | Bridging |  |  |
| SYK | Bridging |  |  |
| PLEKHA4 | Bridging |  |  |
| SQSTM1 | Bridging |  |  |
| CFH | Level 2 | Phuah, 2017 | 28969386 |
| TAF5L | Level 2 |  |  |
| URB2 | Level 2 |  |  |
| GALNT2 | Level 2 |  |  |
| NOTCH4 | Level 2 |  |  |
| ATP6V1B2 | Level 2 |  |  |
| LZTS1 | Level 2 |  |  |
| MPO | Level 2 |  |  |
| TRIM37 | Level 2 |  |  |
| SMG8 | Level 2 |  |  |
| SMAD3 | Bridging |  |  |
| MBIP | Bridging |  |  |
| PLEKHA4 | Bridging |  |  |
| TRIM28 | Bridging |  |  |
| RNF4 | Bridging |  |  |
| HNRNPL | Bridging |  |  |
| NOD2 | Bridging |  |  |
| MDM2 | Bridging |  |  |
| CARD9 | Bridging |  |  |
| POLR1C | Bridging |  |  |
| WDYHV1 | Bridging |  |  |
| ZNF35 | Bridging |  |  |
| IRAK2 | Bridging |  |  |
| TRAF3 | Bridging |  |  |
| SQSTM1 | Bridging |  |  |
| TRAF6 | Bridging |  |  |
| TRAF2 | Bridging |  |  |
| NGFR | Bridging |  |  |
| IKBKG | Bridging |  |  |
| SMURF1 | Bridging |  |  |
| LRRFIP1 | Bridging |  |  |
| LRRFIP2 | Bridging |  |  |
| PRDX1 | Bridging |  |  |
| FLII | Bridging |  |  |
| FBN2 | Level 2 | Saluja, 2024 | 39011893 |
| RNF4 | Bridging |  |  |
| CELSR2 | Level 2 | Shah, 2020 | 31919418 |
| PITX2 | Level 2 |  |  |
| FAM241A | Level 2 |  |  |
| KLHL3 | Level 2 |  |  |
| CDKN1A | Level 2 |  |  |
| BAG3 | Level 2 |  |  |
| ATXN2 | Level 2 |  |  |
| AKT1 | Bridging |  |  |
| SMAD3 | Bridging |  |  |
| RNF4 | Bridging |  |  |
| SQSTM1 | Bridging |  |  |
| MAPK14 | Bridging |  |  |
| TXN | Bridging |  |  |
| TNIP1 | Bridging |  |  |
| MDM2 | Bridging |  |  |
| NGFR | Bridging |  |  |
| HDAC6 | Bridging |  |  |
| IKBKG | Bridging |  |  |
| TSG101 | Bridging |  |  |
| SIAH1 | Bridging |  |  |
| IRAK1 | Bridging |  |  |
| IRAK2 | Bridging |  |  |
| DOCK8 | Bridging |  |  |
| HNRNPL | Bridging |  |  |
| PLEKHA4 | Bridging |  |  |
| PLCB1 | Level 2 | Singh, 2023 | 38104120 |
| RUNX1 | Level 2 |  |  |
| MAPRE2 | Level 2 |  |  |
| PPARA | Level 2 |  |  |
| MAPK14 | Bridging |  |  |
| SMAD3 | Bridging |  |  |
| SIAH1 | Bridging |  |  |
| TRAF6 | Bridging |  |  |
| INS | Bridging |  |  |
| PLEKHA4 | Bridging |  |  |
| HNRNPL | Bridging |  |  |
| BANK1 | Level 1 | van Duijvenboden, 2023 | 37683633 |
| IRF5 | Level 1 |  |  |
| UBAP1 | Level 1 |  |  |
| NFKBIA | Level 1 |  |  |
| SMAD3 | Level 1 |  |  |
| INTS11 | Level 2 |  |  |
| SKI | Level 2 |  |  |
| PRDM16 | Level 2 |  |  |
| CAMTA1 | Level 2 |  |  |
| RERE | Level 2 |  |  |
| SPSB1 | Level 2 |  |  |
| CASZ1 | Level 2 |  |  |
| MTHFR | Level 2 |  |  |
| WNT4 | Level 2 |  |  |
| KDM1A | Level 2 |  |  |
| SRRM1 | Level 2 |  |  |
| ZDHHC18 | Level 2 |  |  |
| WASF2 | Level 2 |  |  |
| AHDC1 | Level 2 |  |  |
| FGR | Level 2 |  |  |
| PHACTR4 | Level 2 |  |  |
| RCC1 | Level 2 |  |  |
| SF3A3 | Level 2 |  |  |
| PABPC4 | Level 2 |  |  |
| HIVEP3 | Level 2 |  |  |
| PIK3R3 | Level 2 |  |  |
| FAF1 | Level 2 |  |  |
| FGGY | Level 2 |  |  |
| DNAJB4 | Level 2 |  |  |
| DDAH1 | Level 2 |  |  |
| ODF2L | Level 2 |  |  |
| PKN2 | Level 2 |  |  |
| GTF2B | Level 2 |  |  |
| BCAR3 | Level 2 |  |  |
| ARHGAP29 | Level 2 |  |  |
| CAPZA1 | Level 2 |  |  |
| MOV10 | Level 2 |  |  |
| POGZ | Level 2 |  |  |
| S100A4 | Level 2 |  |  |
| UBAP2L | Level 2 |  |  |
| NME7 | Level 2 |  |  |
| QSOX1 | Level 2 |  |  |
| XPR1 | Level 2 |  |  |
| LAMC1 | Level 2 |  |  |
| NAV1 | Level 2 |  |  |
| MDM4 | Level 2 |  |  |
| PFKFB2 | Level 2 |  |  |
| CD46 | Level 2 |  |  |
| IRF6 | Level 2 |  |  |
| GPATCH2 | Level 2 |  |  |
| CDC42BPA | Level 2 |  |  |
| WNT3A | Level 2 |  |  |
| SDCCAG8 | Level 2 |  |  |
| MBOAT2 | Level 2 |  |  |
| ASAP2 | Level 2 |  |  |
| APOB | Level 2 |  |  |
| KLHL29 | Level 2 |  |  |
| ADCY3 | Level 2 |  |  |
| FOSL2 | Level 2 |  |  |
| PRKD3 | Level 2 |  |  |
| ZFP36L2 | Level 2 |  |  |
| PRKCE | Level 2 |  |  |
| RTN4 | Level 2 |  |  |
| PNPT1 | Level 2 |  |  |
| USP34 | Level 2 |  |  |
| VPS54 | Level 2 |  |  |
| ZNF638 | Level 2 |  |  |
| EXOC6B | Level 2 |  |  |
| SPR | Level 2 |  |  |
| PTCD3 | Level 2 |  |  |
| ZAP70 | Level 2 |  |  |
| FHL2 | Level 2 |  |  |
| MERTK | Level 2 |  |  |
| PAX8 | Level 2 |  |  |
| GLI2 | Level 2 |  |  |
| ARHGAP15 | Level 2 |  |  |
| ZEB2 | Level 2 |  |  |
| ACVR2A | Level 2 |  |  |
| STAM2 | Level 2 |  |  |
| PRPF40A | Level 2 |  |  |
| ACVR1C | Level 2 |  |  |
| GRB14 | Level 2 |  |  |
| STK39 | Level 2 |  |  |
| MAP3K20 | Level 2 |  |  |
| ZNF385B | Level 2 |  |  |
| SPATS2L | Level 2 |  |  |
| CYP20A1 | Level 2 |  |  |
| ICOS | Level 2 |  |  |
| KLF7 | Level 2 |  |  |
| CPS1 | Level 2 |  |  |
| ERBB4 | Level 2 |  |  |
| FN1 | Level 2 |  |  |
| TNS1 | Level 2 |  |  |
| PNKD | Level 2 |  |  |
| CYP27A1 | Level 2 |  |  |
| COL4A4 | Level 2 |  |  |
| TRIP12 | Level 2 |  |  |
| COPS8 | Level 2 |  |  |
| HDAC4 | Level 2 |  |  |
| FARP2 | Level 2 |  |  |
| ATG7 | Level 2 |  |  |
| WNT7A | Level 2 |  |  |
| FGD5 | Level 2 |  |  |
| KAT2B | Level 2 |  |  |
| UBE2E2 | Level 2 |  |  |
| SLC4A7 | Level 2 |  |  |
| TGFBR2 | Level 2 |  |  |
| GOLGA4 | Level 2 |  |  |
| ITGA9 | Level 2 |  |  |
| CTNNB1 | Level 2 |  |  |
| ULK4 | Level 2 |  |  |
| PRSS50 | Level 2 |  |  |
| MAP4 | Level 2 |  |  |
| CDC25A | Level 2 |  |  |
| ARIH2 | Level 2 |  |  |
| CAMKV | Level 2 |  |  |
| CACNA2D2 | Level 2 |  |  |
| TASOR | Level 2 |  |  |
| SLMAP | Level 2 |  |  |
| ATXN7 | Level 2 |  |  |
| MITF | Level 2 |  |  |
| FOXP1 | Level 2 |  |  |
| PPP4R2 | Level 2 |  |  |
| CMSS1 | Level 2 |  |  |
| SENP7 | Level 2 |  |  |
| TRMT10C | Level 2 |  |  |
| ZBTB20 | Level 2 |  |  |
| ITGB5 | Level 2 |  |  |
| GATA2 | Level 2 |  |  |
| ZBTB38 | Level 2 |  |  |
| MBNL1 | Level 2 |  |  |
| ARHGEF26 | Level 2 |  |  |
| MLF1 | Level 2 |  |  |
| ARL14 | Level 2 |  |  |
| MECOM | Level 2 |  |  |
| FNDC3B | Level 2 |  |  |
| TBL1XR1 | Level 2 |  |  |
| YEATS2 | Level 2 |  |  |
| SENP2 | Level 2 |  |  |
| DLG1 | Level 2 |  |  |
| MAEA | Level 2 |  |  |
| FAM193A | Level 2 |  |  |
| WDR1 | Level 2 |  |  |
| PROM1 | Level 2 |  |  |
| TBC1D19 | Level 2 |  |  |
| ARAP2 | Level 2 |  |  |
| TBC1D1 | Level 2 |  |  |
| RBM47 | Level 2 |  |  |
| GABRA2 | Level 2 |  |  |
| TMEM165 | Level 2 |  |  |
| SHROOM3 | Level 2 |  |  |
| FAM13A | Level 2 |  |  |
| PDLIM5 | Level 2 |  |  |
| BMPR1B | Level 2 |  |  |
| UBE2D3 | Level 2 |  |  |
| NPNT | Level 2 |  |  |
| TBCK | Level 2 |  |  |
| LEF1 | Level 2 |  |  |
| SYNPO2 | Level 2 |  |  |
| GYPA | Level 2 |  |  |
| ZNF827 | Level 2 |  |  |
| LRBA | Level 2 |  |  |
| GUCY1A1 | Level 2 |  |  |
| GUCY1B1 | Level 2 |  |  |
| PALLD | Level 2 |  |  |
| TERT | Level 2 |  |  |
| NPR3 | Level 2 |  |  |
| TARS1 | Level 2 |  |  |
| ITGA1 | Level 2 |  |  |
| ARL15 | Level 2 |  |  |
| ACTBL2 | Level 2 |  |  |
| PLK2 | Level 2 |  |  |
| KIF2A | Level 2 |  |  |
| SREK1 | Level 2 |  |  |
| ADGRV1 | Level 2 |  |  |
| ARRDC3 | Level 2 |  |  |
| ERAP1 | Level 2 |  |  |
| FBXL17 | Level 2 |  |  |
| CSNK1G3 | Level 2 |  |  |
| FBN2 | Level 2 |  |  |
| HSPA4 | Level 2 |  |  |
| ZMAT2 | Level 2 |  |  |
| SPRY4 | Level 2 |  |  |
| DUSP1 | Level 2 |  |  |
| BNIP1 | Level 2 |  |  |
| RNF130 | Level 2 |  |  |
| FOXC1 | Level 2 |  |  |
| GMDS | Level 2 |  |  |
| RNF144B | Level 2 |  |  |
| CDKAL1 | Level 2 |  |  |
| HFE | Level 2 |  |  |
| PRRC2A | Level 2 |  |  |
| ABHD16A | Level 2 |  |  |
| MSH5 | Level 2 |  |  |
| HMGA1 | Level 2 |  |  |
| CDKN1A | Level 2 |  |  |
| ZNF318 | Level 2 |  |  |
| VEGFA | Level 2 |  |  |
| PKHD1 | Level 2 |  |  |
| KCNQ5 | Level 2 |  |  |
| PHIP | Level 2 |  |  |
| BCKDHB | Level 2 |  |  |
| UFL1 | Level 2 |  |  |
| PRDM1 | Level 2 |  |  |
| FOXO3 | Level 2 |  |  |
| FRK | Level 2 |  |  |
| VGLL2 | Level 2 |  |  |
| DCBLD1 | Level 2 |  |  |
| GJA1 | Level 2 |  |  |
| EPB41L2 | Level 2 |  |  |
| CITED2 | Level 2 |  |  |
| AIG1 | Level 2 |  |  |
| STXBP5 | Level 2 |  |  |
| PLEKHG1 | Level 2 |  |  |
| ESR1 | Level 2 |  |  |
| OPRM1 | Level 2 |  |  |
| SLC22A3 | Level 2 |  |  |
| PDE10A | Level 2 |  |  |
| SMOC2 | Level 2 |  |  |
| FOXK1 | Level 2 |  |  |
| HDAC9 | Level 2 |  |  |
| HIBADH | Level 2 |  |  |
| JAZF1 | Level 2 |  |  |
| CREB5 | Level 2 |  |  |
| EEPD1 | Level 2 |  |  |
| GCK | Level 2 |  |  |
| YKT6 | Level 2 |  |  |
| CCM2 | Level 2 |  |  |
| TNS3 | Level 2 |  |  |
| GRB10 | Level 2 |  |  |
| LIMK1 | Level 2 |  |  |
| GTF2I | Level 2 |  |  |
| POM121C | Level 2 |  |  |
| STEAP2 | Level 2 |  |  |
| CDK14 | Level 2 |  |  |
| CDK6 | Level 2 |  |  |
| SEM1 | Level 2 |  |  |
| MUC12 | Level 2 |  |  |
| PIK3CG | Level 2 |  |  |
| COG5 | Level 2 |  |  |
| CAPZA2 | Level 2 |  |  |
| ZC3HC1 | Level 2 |  |  |
| MKLN1 | Level 2 |  |  |
| HIPK2 | Level 2 |  |  |
| ZNF467 | Level 2 |  |  |
| NOS3 | Level 2 |  |  |
| PRKAG2 | Level 2 |  |  |
| MCPH1 | Level 2 |  |  |
| TNKS | Level 2 |  |  |
| MSRA | Level 2 |  |  |
| BLK | Level 2 |  |  |
| SLC7A2 | Level 2 |  |  |
| SORBS3 | Level 2 |  |  |
| SLC25A37 | Level 2 |  |  |
| EBF2 | Level 2 |  |  |
| DPYSL2 | Level 2 |  |  |
| RBPMS | Level 2 |  |  |
| NRG1 | Level 2 |  |  |
| MAK16 | Level 2 |  |  |
| TTI2 | Level 2 |  |  |
| RNF122 | Level 2 |  |  |
| NSD3 | Level 2 |  |  |
| SLC20A2 | Level 2 |  |  |
| PREX2 | Level 2 |  |  |
| RDH10 | Level 2 |  |  |
| RALYL | Level 2 |  |  |
| PDP1 | Level 2 |  |  |
| SNX31 | Level 2 |  |  |
| CCN3 | Level 2 |  |  |
| TRAPPC9 | Level 2 |  |  |
| PTK2 | Level 2 |  |  |
| TSNARE1 | Level 2 |  |  |
| PLEC | Level 2 |  |  |
| KANK1 | Level 2 |  |  |
| PTPRD | Level 2 |  |  |
| PLIN2 | Level 2 |  |  |
| MTAP | Level 2 |  |  |
| FRMD3 | Level 2 |  |  |
| C9ORF170 | Level 2 |  |  |
| CENPP | Level 2 |  |  |
| ZNF462 | Level 2 |  |  |
| SVEP1 | Level 2 |  |  |
| ZNF618 | Level 2 |  |  |
| PAPPA | Level 2 |  |  |
| PSMD5 | Level 2 |  |  |
| RABGAP1 | Level 2 |  |  |
| NEK6 | Level 2 |  |  |
| SCAI | Level 2 |  |  |
| PPP6C | Level 2 |  |  |
| PBX3 | Level 2 |  |  |
| NIBAN2 | Level 2 |  |  |
| CELF2 | Level 2 |  |  |
| CACNB2 | Level 2 |  |  |
| PLXDC2 | Level 2 |  |  |
| NEBL | Level 2 |  |  |
| WAC | Level 2 |  |  |
| ZNF438 | Level 2 |  |  |
| ARHGAP12 | Level 2 |  |  |
| ANK3 | Level 2 |  |  |
| REEP3 | Level 2 |  |  |
| SIRT1 | Level 2 |  |  |
| PTEN | Level 2 |  |  |
| RNLS | Level 2 |  |  |
| ACTA2 | Level 2 |  |  |
| PLCE1 | Level 2 |  |  |
| BTRC | Level 2 |  |  |
| LDB1 | Level 2 |  |  |
| NT5C2 | Level 2 |  |  |
| STN1 | Level 2 |  |  |
| TCF7L2 | Level 2 |  |  |
| HSPA12A | Level 2 |  |  |
| BAG3 | Level 2 |  |  |
| FGFR2 | Level 2 |  |  |
| PPP2R2D | Level 2 |  |  |
| INPP5A | Level 2 |  |  |
| LSP1 | Level 2 |  |  |
| TNNT3 | Level 2 |  |  |
| LMO1 | Level 2 |  |  |
| DENND2B | Level 2 |  |  |
| SWAP70 | Level 2 |  |  |
| ARNTL | Level 2 |  |  |
| SOX6 | Level 2 |  |  |
| PLEKHA7 | Level 2 |  |  |
| NUCB2 | Level 2 |  |  |
| BDNF | Level 2 |  |  |
| METTL15 | Level 2 |  |  |
| ARL14EP | Level 2 |  |  |
| WT1 | Level 2 |  |  |
| CREB3L1 | Level 2 |  |  |
| LRP4 | Level 2 |  |  |
| PSMC3 | Level 2 |  |  |
| PTPMT1 | Level 2 |  |  |
| CTNND1 | Level 2 |  |  |
| LRRC10B | Level 2 |  |  |
| EHBP1L1 | Level 2 |  |  |
| SIPA1 | Level 2 |  |  |
| CLPB | Level 2 |  |  |
| FAM168A | Level 2 |  |  |
| POLD3 | Level 2 |  |  |
| THAP12 | Level 2 |  |  |
| CLNS1A | Level 2 |  |  |
| GAB2 | Level 2 |  |  |
| NOX4 | Level 2 |  |  |
| FAM76B | Level 2 |  |  |
| ARHGAP42 | Level 2 |  |  |
| PGR | Level 2 |  |  |
| YAP1 | Level 2 |  |  |
| CWF19L2 | Level 2 |  |  |
| SIK2 | Level 2 |  |  |
| CEP164 | Level 2 |  |  |
| UBASH3B | Level 2 |  |  |
| RAD52 | Level 2 |  |  |
| CACNA1C | Level 2 |  |  |
| CCND2 | Level 2 |  |  |
| DUSP16 | Level 2 |  |  |
| PDE3A | Level 2 |  |  |
| SOX5 | Level 2 |  |  |
| BCAT1 | Level 2 |  |  |
| STK38L | Level 2 |  |  |
| PPHLN1 | Level 2 |  |  |
| HDAC7 | Level 2 |  |  |
| H1-7 | Level 2 |  |  |
| PRKAG1 | Level 2 |  |  |
| DIP2B | Level 2 |  |  |
| HOXC4 | Level 2 |  |  |
| NACA | Level 2 |  |  |
| LRP1 | Level 2 |  |  |
| ARHGEF25 | Level 2 |  |  |
| TMBIM4 | Level 2 |  |  |
| CAND1 | Level 2 |  |  |
| RAB3IP | Level 2 |  |  |
| CNOT2 | Level 2 |  |  |
| SYT1 | Level 2 |  |  |
| ATP2B1 | Level 2 |  |  |
| CEP83 | Level 2 |  |  |
| IGF1 | Level 2 |  |  |
| RPL6 | Level 2 |  |  |
| TBX3 | Level 2 |  |  |
| SBNO1 | Level 2 |  |  |
| NCOR2 | Level 2 |  |  |
| FBRSL1 | Level 2 |  |  |
| WASF3 | Level 2 |  |  |
| SLC7A1 | Level 2 |  |  |
| MRPS31 | Level 2 |  |  |
| NAA16 | Level 2 |  |  |
| DGKH | Level 2 |  |  |
| LRCH1 | Level 2 |  |  |
| TRIM13 | Level 2 |  |  |
| PRR20A | Level 2 |  |  |
| PCDH17 | Level 2 |  |  |
| DACH1 | Level 2 |  |  |
| KLF5 | Level 2 |  |  |
| OBI1 | Level 2 |  |  |
| RBM26 | Level 2 |  |  |
| SPRY2 | Level 2 |  |  |
| MBNL2 | Level 2 |  |  |
| COL4A2 | Level 2 |  |  |
| MCF2L | Level 2 |  |  |
| CDC16 | Level 2 |  |  |
| PRKD1 | Level 2 |  |  |
| SNX6 | Level 2 |  |  |
| FBXO33 | Level 2 |  |  |
| SOS2 | Level 2 |  |  |
| L2HGDH | Level 2 |  |  |
| CDKL1 | Level 2 |  |  |
| FERMT2 | Level 2 |  |  |
| PPM1A | Level 2 |  |  |
| PLEKHH1 | Level 2 |  |  |
| ZFP36L1 | Level 2 |  |  |
| FOXN3 | Level 2 |  |  |
| RIN3 | Level 2 |  |  |
| YY1 | Level 2 |  |  |
| MARK3 | Level 2 |  |  |
| ATP10A | Level 2 |  |  |
| EIF2AK4 | Level 2 |  |  |
| INO80 | Level 2 |  |  |
| MAPKBP1 | Level 2 |  |  |
| TRPM7 | Level 2 |  |  |
| PIAS1 | Level 2 |  |  |
| CCDC33 | Level 2 |  |  |
| ULK3 | Level 2 |  |  |
| MORF4L1 | Level 2 |  |  |
| PDE8A | Level 2 |  |  |
| AKAP13 | Level 2 |  |  |
| IDH2 | Level 2 |  |  |
| FURIN | Level 2 |  |  |
| FES | Level 2 |  |  |
| SLCO3A1 | Level 2 |  |  |
| CHD2 | Level 2 |  |  |
| MCTP2 | Level 2 |  |  |
| MEF2A | Level 2 |  |  |
| UBE2I | Level 2 |  |  |
| SLC9A3R2 | Level 2 |  |  |
| ADCY9 | Level 2 |  |  |
| PPL | Level 2 |  |  |
| MYH11 | Level 2 |  |  |
| TNRC6A | Level 2 |  |  |
| ZNF423 | Level 2 |  |  |
| CNEP1R1 | Level 2 |  |  |
| NKD1 | Level 2 |  |  |
| CNOT1 | Level 2 |  |  |
| PDP2 | Level 2 |  |  |
| NFAT5 | Level 2 |  |  |
| VAC14 | Level 2 |  |  |
| ZFHX3 | Level 2 |  |  |
| CFDP1 | Level 2 |  |  |
| CDYL2 | Level 2 |  |  |
| CMIP | Level 2 |  |  |
| BANP | Level 2 |  |  |
| CRK | Level 2 |  |  |
| SMG6 | Level 2 |  |  |
| ATP2A3 | Level 2 |  |  |
| ZZEF1 | Level 2 |  |  |
| PITPNM3 | Level 2 |  |  |
| KIAA0753 | Level 2 |  |  |
| TP53 | Level 2 |  |  |
| SLC2A4 | Level 2 |  |  |
| TOP3A | Level 2 |  |  |
| SMCR8 | Level 2 |  |  |
| SUPT6H | Level 2 |  |  |
| ERAL1 | Level 2 |  |  |
| ATAD5 | Level 2 |  |  |
| GPATCH8 | Level 2 |  |  |
| KANSL1 | Level 2 |  |  |
| GOSR2 | Level 2 |  |  |
| HOXB7 | Level 2 |  |  |
| ZNF652 | Level 2 |  |  |
| MSI2 | Level 2 |  |  |
| VMP1 | Level 2 |  |  |
| BCAS3 | Level 2 |  |  |
| C17ORF82 | Level 2 |  |  |
| MRC2 | Level 2 |  |  |
| PECAM1 | Level 2 |  |  |
| APOH | Level 2 |  |  |
| SEPTIN9 | Level 2 |  |  |
| CYTH1 | Level 2 |  |  |
| YES1 | Level 2 |  |  |
| FHOD3 | Level 2 |  |  |
| SETBP1 | Level 2 |  |  |
| SLC14A2 | Level 2 |  |  |
| MAPK4 | Level 2 |  |  |
| MEX3C | Level 2 |  |  |
| TCF4 | Level 2 |  |  |
| WDR7 | Level 2 |  |  |
| NEDD4L | Level 2 |  |  |
| MC4R | Level 2 |  |  |
| ZCCHC2 | Level 2 |  |  |
| BCL2 | Level 2 |  |  |
| RNF126 | Level 2 |  |  |
| DAZAP1 | Level 2 |  |  |
| ZBTB7A | Level 2 |  |  |
| MAP2K2 | Level 2 |  |  |
| UHRF1 | Level 2 |  |  |
| KDM4B | Level 2 |  |  |
| INSR | Level 2 |  |  |
| MRPL4 | Level 2 |  |  |
| NOTCH3 | Level 2 |  |  |
| KLF2 | Level 2 |  |  |
| MYO9B | Level 2 |  |  |
| ZNF101 | Level 2 |  |  |
| ZNF100 | Level 2 |  |  |
| CCNE1 | Level 2 |  |  |
| TSHZ3 | Level 2 |  |  |
| PEPD | Level 2 |  |  |
| KIAA0355 | Level 2 |  |  |
| AKT2 | Level 2 |  |  |
| SPTBN4 | Level 2 |  |  |
| SNRNP70 | Level 2 |  |  |
| SPIB | Level 2 |  |  |
| TMEM239 | Level 2 |  |  |
| PLCB1 | Level 2 |  |  |
| SLX4IP | Level 2 |  |  |
| JAG1 | Level 2 |  |  |
| BTBD3 | Level 2 |  |  |
| ID1 | Level 2 |  |  |
| DNMT3B | Level 2 |  |  |
| CDK5RAP1 | Level 2 |  |  |
| DYNLRB1 | Level 2 |  |  |
| PROCR | Level 2 |  |  |
| CHD6 | Level 2 |  |  |
| PREX1 | Level 2 |  |  |
| NFATC2 | Level 2 |  |  |
| APC | Level 2 |  |  |
| TCEA2 | Level 2 |  |  |
| NRIP1 | Level 2 |  |  |
| EVA1C | Level 2 |  |  |
| UMODL1 | Level 2 |  |  |
| CRYAA | Level 2 |  |  |
| SIK1 | Level 2 |  |  |
| RRP1B | Level 2 |  |  |
| COL6A1 | Level 2 |  |  |
| DIP2A | Level 2 |  |  |
| ARVCF | Level 2 |  |  |
| TTC28 | Level 2 |  |  |
| TRIOBP | Level 2 |  |  |
| TNRC6B | Level 2 |  |  |
| XRCC6 | Level 2 |  |  |
| BRD1 | Level 2 |  |  |
| PLXNB2 | Level 2 |  |  |
| TRIM28 | Bridging |  |  |
| SIAH2 | Bridging |  |  |
| SMAD3 | Bridging |  |  |
| HNRNPL | Bridging |  |  |
| SQSTM1 | Bridging |  |  |
| SMURF1 | Bridging |  |  |
| AKT1 | Bridging |  |  |
| PRDX1 | Bridging |  |  |
| TSG101 | Bridging |  |  |
| PFKL | Bridging |  |  |
| RNF4 | Bridging |  |  |
| MBIP | Bridging |  |  |
| TLR9 | Bridging |  |  |
| TLR10 | Bridging |  |  |
| HDAC6 | Bridging |  |  |
| USP7 | Bridging |  |  |
| PLEKHA4 | Bridging |  |  |
| SPOP | Bridging |  |  |
| INS | Bridging |  |  |
| BTK | Bridging |  |  |
| RAC1 | Bridging |  |  |
| SYK | Bridging |  |  |
| MAPK14 | Bridging |  |  |
| IKBKG | Bridging |  |  |
| TRAF6 | Bridging |  |  |
| MDM2 | Bridging |  |  |
| NGFR | Bridging |  |  |
| IKBKE | Bridging |  |  |
| CYLD | Bridging |  |  |
| TRAF2 | Bridging |  |  |
| PIK3AP1 | Bridging |  |  |
| IKBKB | Bridging |  |  |
| FADD | Bridging |  |  |
| GLOD4 | Bridging |  |  |
| CBLB | Bridging |  |  |
| TNIP1 | Bridging |  |  |
| MAP3K7 | Bridging |  |  |
| LRRFIP2 | Bridging |  |  |
| ATG5 | Bridging |  |  |
| RIPK2 | Bridging |  |  |
| UBAP1 | Bridging |  |  |
| AGER | Bridging |  |  |
| MAP4K4 | Bridging |  |  |
| TNFRSF1A | Bridging |  |  |
| PELI1 | Bridging |  |  |
| DOCK8 | Bridging |  |  |
| IRF5 | Bridging |  |  |
| PTK2B | Bridging |  |  |
| CYTH2 | Bridging |  |  |
| TXN | Bridging |  |  |
| TNFRSF13B | Bridging |  |  |
| IRAK1 | Bridging |  |  |
| CD14 | Bridging |  |  |
| CD93 | Bridging |  |  |
| TLR2 | Bridging |  |  |
| MAP3K1 | Bridging |  |  |
| IL1RAP | Bridging |  |  |
| ZNF35 | Bridging |  |  |
| TBK1 | Bridging |  |  |
| PELI2 | Bridging |  |  |
| POLR1C | Bridging |  |  |
| NFKBIA | Bridging |  |  |
| IL1B | Bridging |  |  |
| FLII | Bridging |  |  |
| IRF7 | Bridging |  |  |
| SIAH1 | Bridging |  |  |
| LRRFIP1 | Bridging |  |  |
| TLR7 | Bridging |  |  |
| NOD2 | Bridging |  |  |
| SMAD6 | Bridging |  |  |
| SARM1 | Bridging |  |  |
| TLR4 | Bridging |  |  |
| WDYHV1 | Bridging |  |  |
| TRAF3 | Bridging |  |  |
| CAMLG | Bridging |  |  |
| IRF4 | Bridging |  |  |
| CISH | Bridging |  |  |
| SASH1 | Bridging |  |  |
| MAL | Bridging |  |  |
| SPOPL | Bridging |  |  |
| BST2 | Bridging |  |  |
| BANK1 | Bridging |  |  |
| STAP2 | Bridging |  |  |
| TLR3 | Bridging |  |  |
| IRAK3 | Bridging |  |  |
| IRAK2 | Bridging |  |  |
| CARD9 | Bridging |  |  |
| IL1RL1 | Bridging |  |  |
| IRAK4 | Bridging |  |  |
| CASP1 | Bridging |  |  |
| IL1R1 | Bridging |  |  |
| TLR8 | Bridging |  |  |
| TLR5 | Bridging |  |  |
| PIK3CG | Level 2 | Wain, 2011 | 21909110 |
| CCN3 | Level 2 |  |  |
| MAP4 | Level 2 |  |  |
| SYK | Bridging |  |  |
| PIK3AP1 | Bridging |  |  |
| NGFR | Bridging |  |  |
| AKT1 | Bridging |  |  |
| INS | Bridging |  |  |
| HNRNPL | Bridging |  |  |
| CYLD | Bridging |  |  |
| PLEKHA4 | Bridging |  |  |
| NFKBIA | Level 1 | Wain, 2017 | 28739976 |
| EBF2 | Level 2 |  |  |
| SEPTIN9 | Level 2 |  |  |
| AKT2 | Level 2 |  |  |
| TRAF2 | Bridging |  |  |
| SYK | Bridging |  |  |
| MAPK14 | Bridging |  |  |
| IKBKB | Bridging |  |  |
| IRAK1 | Bridging |  |  |
| NGFR | Bridging |  |  |
| IKBKG | Bridging |  |  |
| TLR2 | Bridging |  |  |
| MAP3K7 | Bridging |  |  |
| IRAK4 | Bridging |  |  |
| TBK1 | Bridging |  |  |
| AKT1 | Bridging |  |  |
| IKBKE | Bridging |  |  |
| CD14 | Bridging |  |  |
| MAP3K1 | Bridging |  |  |
| HNRNPL | Bridging |  |  |
| TRAF6 | Bridging |  |  |
| TRIM28 | Bridging |  |  |
| CYLD | Bridging |  |  |
| PLEKHA4 | Bridging |  |  |
| INTS11 | Level 2 | Warren, 2017 | 28135244 |
| CELA2A | Level 2 |  |  |
| GTF2B | Level 2 |  |  |
| FOSL2 | Level 2 |  |  |
| PRKD3 | Level 2 |  |  |
| NPNT | Level 2 |  |  |
| FBN2 | Level 2 |  |  |
| MKLN1 | Level 2 |  |  |
| HIPK2 | Level 2 |  |  |
| MCF2L | Level 2 |  |  |
| FERMT2 | Level 2 |  |  |
| PPP2R5E | Level 2 |  |  |
| CFDP1 | Level 2 |  |  |
| CRK | Level 2 |  |  |
| GPATCH2 | Level 2 |  |  |
| CDC42BPA | Level 2 |  |  |
| WNT3A | Level 2 |  |  |
| SDCCAG8 | Level 2 |  |  |
| ADCY3 | Level 2 |  |  |
| TNS1 | Level 2 |  |  |
| CAMKV | Level 2 |  |  |
| CACNA2D2 | Level 2 |  |  |
| TASOR | Level 2 |  |  |
| SENP2 | Level 2 |  |  |
| PKHD1 | Level 2 |  |  |
| PDE10A | Level 2 |  |  |
| SNX31 | Level 2 |  |  |
| MTAP | Level 2 |  |  |
| BDNF | Level 2 |  |  |
| PPL | Level 2 |  |  |
| CMIP | Level 2 |  |  |
| MAPK4 | Level 2 |  |  |
| CCNE1 | Level 2 |  |  |
| PLCB1 | Level 2 |  |  |
| SF3A3 | Level 2 |  |  |
| FGGY | Level 2 |  |  |
| PRKCE | Level 2 |  |  |
| FN1 | Level 2 |  |  |
| GATA2 | Level 2 |  |  |
| PALLD | Level 2 |  |  |
| GJA1 | Level 2 |  |  |
| ESR1 | Level 2 |  |  |
| SLC20A2 | Level 2 |  |  |
| TRAPPC9 | Level 2 |  |  |
| SCAI | Level 2 |  |  |
| ARHGAP12 | Level 2 |  |  |
| NOX4 | Level 2 |  |  |
| CEP164 | Level 2 |  |  |
| CEP83 | Level 2 |  |  |
| VAC14 | Level 2 |  |  |
| KIAA0753 | Level 2 |  |  |
| TP53 | Level 2 |  |  |
| SLC2A4 | Level 2 |  |  |
| MRC2 | Level 2 |  |  |
| SLC14A2 | Level 2 |  |  |
| ARVCF | Level 2 |  |  |
| XRCC6 | Level 2 |  |  |
| ZNF638 | Level 2 |  |  |
| TRIM28 | Bridging |  |  |
| CYLD | Bridging |  |  |
| SARM1 | Bridging |  |  |
| CBLB | Bridging |  |  |
| TNIP1 | Bridging |  |  |
| SMURF1 | Bridging |  |  |
| PLEKHA4 | Bridging |  |  |
| HNRNPL | Bridging |  |  |
| CYTH2 | Bridging |  |  |
| IKBKG | Bridging |  |  |
| INS | Bridging |  |  |
| RNF4 | Bridging |  |  |
| HDAC6 | Bridging |  |  |
| SIAH2 | Bridging |  |  |
| SMAD3 | Bridging |  |  |
| SIAH1 | Bridging |  |  |
| MDM2 | Bridging |  |  |
| NGFR | Bridging |  |  |
| MAP3K7 | Bridging |  |  |
| SPOP | Bridging |  |  |
| MAP3K1 | Bridging |  |  |
| RAC1 | Bridging |  |  |
| NOD2 | Bridging |  |  |
| SYK | Bridging |  |  |
| PTK2B | Bridging |  |  |
| TSG101 | Bridging |  |  |
| CD93 | Bridging |  |  |
| SASH1 | Bridging |  |  |
| TRAF2 | Bridging |  |  |
| TRAF6 | Bridging |  |  |
| LRRFIP2 | Bridging |  |  |
| TLR9 | Bridging |  |  |
| SQSTM1 | Bridging |  |  |
| CAMLG | Bridging |  |  |
| TXN | Bridging |  |  |
| AGER | Bridging |  |  |
| USP7 | Bridging |  |  |
| AKT1 | Bridging |  |  |
| IRAK1 | Bridging |  |  |
| CISH | Bridging |  |  |
| MAPK14 | Bridging |  |  |
| BTK | Bridging |  |  |
| MAP4K4 | Bridging |  |  |
| IKBKB | Bridging |  |  |
| PELI2 | Bridging |  |  |
| POLR1C | Bridging |  |  |
| PRDX1 | Bridging |  |  |
| PFKL | Bridging |  |  |
| ATG5 | Bridging |  |  |
| TRAF3 | Bridging |  |  |
| LRRFIP1 | Bridging |  |  |
| TBK1 | Bridging |  |  |
| IKBKE | Bridging |  |  |
| FLII | Bridging |  |  |
| TNFRSF1A | Bridging |  |  |
| TLR4 | Bridging |  |  |
| CASP1 | Bridging |  |  |
| PELI1 | Bridging |  |  |
| WDYHV1 | Bridging |  |  |
| NFKBIA | Bridging |  |  |
| IRF7 | Bridging |  |  |
| TLR5 | Bridging |  |  |
| TNFRSF13B | Bridging |  |  |
| TSC22D3 | Level 2 | Zeller, 2017 | 28784648 |
| CEBPA | Level 2 |  |  |
| F12 | Level 2 |  |  |
| LMNA | Level 2 |  |  |
| MDM2 | Bridging |  |  |
| SMAD3 | Bridging |  |  |
| CD93 | Bridging |  |  |
| CASP1 | Bridging |  |  |
| SIAH1 | Bridging |  |  |
| HNRNPL | Bridging |  |  |
| IRAK1 | Bridging |  |  |
| TRIM28 | Bridging |  |  |
| HDAC6 | Bridging |  |  |
| USP7 | Bridging |  |  |
| PELI1 | Bridging |  |  |
| RNF4 | Bridging |  |  |
| SMURF1 | Bridging |  |  |
| NOD2 | Bridging |  |  |
| ATG5 | Bridging |  |  |
| CAMLG | Bridging |  |  |
| CYLD | Bridging |  |  |
| WDYHV1 | Bridging |  |  |
| PLEKHA4 | Bridging |  |  |
| MAL | Bridging |  |  |
| DNAJC11 | Level 2 | Zhang, 2024 | 38872756 |
| THAP3 | Level 2 |  |  |
| MTHFR | Level 2 |  |  |
| CELA2A | Level 2 |  |  |
| DNAJC16 | Level 2 |  |  |
| PKN2 | Level 2 |  |  |
| KYAT3 | Level 2 |  |  |
| YOD1 | Level 2 |  |  |
| ADCY3 | Level 2 |  |  |
| GPN1 | Level 2 |  |  |
| THADA | Level 2 |  |  |
| PPP4R3B | Level 2 |  |  |
| TNS1 | Level 2 |  |  |
| FGD5 | Level 2 |  |  |
| SLC4A7 | Level 2 |  |  |
| SMARCC1 | Level 2 |  |  |
| MAP4 | Level 2 |  |  |
| ZNF589 | Level 2 |  |  |
| EAF2 | Level 2 |  |  |
| ARHGEF26 | Level 2 |  |  |
| TBCK | Level 2 |  |  |
| AIMP1 | Level 2 |  |  |
| USP53 | Level 2 |  |  |
| PCDH18 | Level 2 |  |  |
| ARL15 | Level 2 |  |  |
| IRF1 | Level 2 |  |  |
| NDUFA2 | Level 2 |  |  |
| H2AC4 | Level 2 |  |  |
| CDSN | Level 2 |  |  |
| CCHCR1 | Level 2 |  |  |
| HLA-B | Level 2 |  |  |
| PRRC2A | Level 2 |  |  |
| CSNK2B | Level 2 |  |  |
| NOTCH4 | Level 2 |  |  |
| PHIP | Level 2 |  |  |
| UFL1 | Level 2 |  |  |
| GOPC | Level 2 |  |  |
| ERI1 | Level 2 |  |  |
| MSRA | Level 2 |  |  |
| FDFT1 | Level 2 |  |  |
| FGFR1 | Level 2 |  |  |
| PTK2 | Level 2 |  |  |
| TSNARE1 | Level 2 |  |  |
| ZBTB6 | Level 2 |  |  |
| GAPVD1 | Level 2 |  |  |
| MAPKAP1 | Level 2 |  |  |
| JMJD1C | Level 2 |  |  |
| SEC24C | Level 2 |  |  |
| PRXL2A | Level 2 |  |  |
| PDCD11 | Level 2 |  |  |
| PLEKHA1 | Level 2 |  |  |
| PDE3B | Level 2 |  |  |
| NUCB2 | Level 2 |  |  |
| MADD | Level 2 |  |  |
| SPI1 | Level 2 |  |  |
| CWF19L2 | Level 2 |  |  |
| HDAC7 | Level 2 |  |  |
| POC1B | Level 2 |  |  |
| ATXN2 | Level 2 |  |  |
| NAA16 | Level 2 |  |  |
| TRPM7 | Level 2 |  |  |
| ULK3 | Level 2 |  |  |
| PPCDC | Level 2 |  |  |
| AKAP13 | Level 2 |  |  |
| MEF2A | Level 2 |  |  |
| GLYR1 | Level 2 |  |  |
| PPL | Level 2 |  |  |
| TNRC6A | Level 2 |  |  |
| CMTM3 | Level 2 |  |  |
| CFDP1 | Level 2 |  |  |
| SMG6 | Level 2 |  |  |
| SRR | Level 2 |  |  |
| KIAA0753 | Level 2 |  |  |
| NMT1 | Level 2 |  |  |
| HEXIM1 | Level 2 |  |  |
| PLEKHM1 | Level 2 |  |  |
| MAPT | Level 2 |  |  |
| KANSL1 | Level 2 |  |  |
| NSF | Level 2 |  |  |
| GOSR2 | Level 2 |  |  |
| SCRN2 | Level 2 |  |  |
| IGF2BP1 | Level 2 |  |  |
| ZNF652 | Level 2 |  |  |
| RAD51C | Level 2 |  |  |
| BCAS3 | Level 2 |  |  |
| PECAM1 | Level 2 |  |  |
| DDX5 | Level 2 |  |  |
| TRIM65 | Level 2 |  |  |
| SEPTIN9 | Level 2 |  |  |
| CYTH1 | Level 2 |  |  |
| MAPK4 | Level 2 |  |  |
| S1PR2 | Level 2 |  |  |
| JAG1 | Level 2 |  |  |
| ID1 | Level 2 |  |  |
| DNAJC5 | Level 2 |  |  |
| NRIP1 | Level 2 |  |  |
| PLXNB2 | Level 2 |  |  |
| UBAP2L | Level 2 |  |  |
| HAX1 | Level 2 |  |  |
| IPO9 | Level 2 |  |  |
| MDM4 | Level 2 |  |  |
| CEP170 | Level 2 |  |  |
| SDCCAG8 | Level 2 |  |  |
| USP34 | Level 2 |  |  |
| XPO1 | Level 2 |  |  |
| NCAPH | Level 2 |  |  |
| CCNT2 | Level 2 |  |  |
| ORC4 | Level 2 |  |  |
| SLMAP | Level 2 |  |  |
| ATXN7 | Level 2 |  |  |
| AMOTL2 | Level 2 |  |  |
| MECOM | Level 2 |  |  |
| PLK2 | Level 2 |  |  |
| SLC22A4 | Level 2 |  |  |
| PRELID1 | Level 2 |  |  |
| HLA-A | Level 2 |  |  |
| VARS2 | Level 2 |  |  |
| MSH5 | Level 2 |  |  |
| HLA-DQB2 | Level 2 |  |  |
| FRK | Level 2 |  |  |
| FAM91A1 | Level 2 |  |  |
| TLN1 | Level 2 |  |  |
| ACTR1A | Level 2 |  |  |
| NT5C2 | Level 2 |  |  |
| BNIP3 | Level 2 |  |  |
| BDNF | Level 2 |  |  |
| METTL15 | Level 2 |  |  |
| ATP2A2 | Level 2 |  |  |
| MAPKAPK5 | Level 2 |  |  |
| TRAFD1 | Level 2 |  |  |
| CDK2AP1 | Level 2 |  |  |
| SBNO1 | Level 2 |  |  |
| PLA2G4B | Level 2 |  |  |
| MPI | Level 2 |  |  |
| FURIN | Level 2 |  |  |
| RHOT2 | Level 2 |  |  |
| TBL3 | Level 2 |  |  |
| POLI | Level 2 |  |  |
| ZNF443 | Level 2 |  |  |
| ZC3H4 | Level 2 |  |  |
| SLX4IP | Level 2 |  |  |
| PREX1 | Level 2 |  |  |
| RTEL1 | Level 2 |  |  |
| SENP7 | Level 2 |  |  |
| MLF1 | Level 2 |  |  |
| FBN2 | Level 2 |  |  |
| PPP6C | Level 2 |  |  |
| RSF1 | Level 2 |  |  |
| PHETA1 | Level 2 |  |  |
| SNTB2 | Level 2 |  |  |
| SPATA2L | Level 2 |  |  |
| YES1 | Level 2 |  |  |
| SHKBP1 | Level 2 |  |  |
| UCKL1 | Level 2 |  |  |
| HLA-DRA | Level 2 |  |  |
| SNF8 | Level 2 |  |  |
| SIK1 | Level 2 |  |  |
| AP3M1 | Level 2 |  |  |
| MPHOSPH9 | Level 2 |  |  |
| HAUS8 | Level 2 |  |  |
| HM13 | Level 2 |  |  |
| INS | Bridging |  |  |
| PLEKHA4 | Bridging |  |  |
| RAC1 | Bridging |  |  |
| TLR9 | Bridging |  |  |
| TLR10 | Bridging |  |  |
| CYLD | Bridging |  |  |
| SARM1 | Bridging |  |  |
| CAMLG | Bridging |  |  |
| AKT1 | Bridging |  |  |
| HNRNPL | Bridging |  |  |
| TRAF6 | Bridging |  |  |
| POLR1C | Bridging |  |  |
| TNFRSF1A | Bridging |  |  |
| SYK | Bridging |  |  |
| TRIM28 | Bridging |  |  |
| RNF4 | Bridging |  |  |
| LRRFIP2 | Bridging |  |  |
| FLII | Bridging |  |  |
| SMAD3 | Bridging |  |  |
| IKBKB | Bridging |  |  |
| HDAC6 | Bridging |  |  |
| IKBKE | Bridging |  |  |
| SIAH2 | Bridging |  |  |
| MAPK14 | Bridging |  |  |
| TRAF2 | Bridging |  |  |
| ATG5 | Bridging |  |  |
| TRAF3 | Bridging |  |  |
| MDM2 | Bridging |  |  |
| SPOP | Bridging |  |  |
| USP7 | Bridging |  |  |
| IKBKG | Bridging |  |  |
| TSG101 | Bridging |  |  |
| CARD9 | Bridging |  |  |
| WDYHV1 | Bridging |  |  |
| TXN | Bridging |  |  |
| IRAK1 | Bridging |  |  |
| PFKL | Bridging |  |  |
| SQSTM1 | Bridging |  |  |
| MAP4K4 | Bridging |  |  |
| NFKBIA | Bridging |  |  |
| TBK1 | Bridging |  |  |
| MAP3K7 | Bridging |  |  |
| PRDX1 | Bridging |  |  |
| AGER | Bridging |  |  |
| PTK2B | Bridging |  |  |
| CBLB | Bridging |  |  |
| STAP2 | Bridging |  |  |
| MAL | Bridging |  |  |
| TLR5 | Bridging |  |  |
| IRF4 | Bridging |  |  |
| ZNF35 | Bridging |  |  |
| TNIP1 | Bridging |  |  |
| FADD | Bridging |  |  |
| BTK | Bridging |  |  |
| CASP1 | Bridging |  |  |
| LRRFIP1 | Bridging |  |  |
| IRF7 | Bridging |  |  |
| MBIP | Bridging |  |  |
| PELI1 | Bridging |  |  |
| CD14 | Bridging |  |  |
| SMURF1 | Bridging |  |  |
| CYTH2 | Bridging |  |  |
| DOCK8 | Bridging |  |  |
| IRF5 | Bridging |  |  |
| MAP3K1 | Bridging |  |  |
| UBAP1 | Bridging |  |  |
| CD93 | Bridging |  |  |
| SIAH1 | Bridging |  |  |
| NGFR | Bridging |  |  |
| TLR7 | Bridging |  |  |
| IL1R1 | Bridging |  |  |
| TLR3 | Bridging |  |  |
| SMAD3 | Level 1 | Zheng, 2024 | 38969659 |
| AARS1 | Level 2 |  |  |
| ABCB8 | Level 2 |  |  |
| ACOT8 | Level 2 |  |  |
| ACP2 | Level 2 |  |  |
| ADAM10 | Level 2 |  |  |
| HYKK | Level 2 |  |  |
| AMBRA1 | Level 2 |  |  |
| ANGPTL4 | Level 2 |  |  |
| ANTXR1 | Level 2 |  |  |
| AP1G1 | Level 2 |  |  |
| APEH | Level 2 |  |  |
| COA8 | Level 2 |  |  |
| ARHGAP1 | Level 2 |  |  |
| ARHGAP42 | Level 2 |  |  |
| ARID5B | Level 2 |  |  |
| ARIH1 | Level 2 |  |  |
| ARIH2 | Level 2 |  |  |
| ARNTL | Level 2 |  |  |
| ASZ1 | Level 2 |  |  |
| ATG13 | Level 2 |  |  |
| ATP2B1 | Level 2 |  |  |
| ATXN1L | Level 2 |  |  |
| ATXN2 | Level 2 |  |  |
| BAD | Level 2 |  |  |
| BAG5 | Level 2 |  |  |
| BCAS3 | Level 2 |  |  |
| BCKDK | Level 2 |  |  |
| BLK | Level 2 |  |  |
| BSN | Level 2 |  |  |
| C17ORF82 | Level 2 |  |  |
| C1ORF105 | Level 2 |  |  |
| C1ORF35 | Level 2 |  |  |
| C1ORF43 | Level 2 |  |  |
| CACNB2 | Level 2 |  |  |
| CADM4 | Level 2 |  |  |
| CAMK2G | Level 2 |  |  |
| CARM1 | Level 2 |  |  |
| CASZ1 | Level 2 |  |  |
| CCDC36 | Level 2 |  |  |
| CCDC88B | Level 2 |  |  |
| CD40 | Level 2 |  |  |
| CDC25A | Level 2 |  |  |
| CDKN1A | Level 2 |  |  |
| CDKN1B | Level 2 |  |  |
| CDKN2A | Level 2 |  |  |
| CDKN2B | Level 2 |  |  |
| CELF1 | Level 2 |  |  |
| CELSR2 | Level 2 |  |  |
| CEP57 | Level 2 |  |  |
| CHD3 | Level 2 |  |  |
| CHMP1A | Level 2 |  |  |
| CHRM4 | Level 2 |  |  |
| CHRNB4 | Level 2 |  |  |
| CKAP5 | Level 2 |  |  |
| CKB | Level 2 |  |  |
| CLNS1A | Level 2 |  |  |
| CLPTM1 | Level 2 |  |  |
| CLTC | Level 2 |  |  |
| CMIP | Level 2 |  |  |
| CNNM2 | Level 2 |  |  |
| CNPY4 | Level 2 |  |  |
| CPA4 | Level 2 |  |  |
| CREB3L1 | Level 2 |  |  |
| CRTC2 | Level 2 |  |  |
| CTTNBP2NL | Level 2 |  |  |
| CWF19L2 | Level 2 |  |  |
| CYB5D1 | Level 2 |  |  |
| CYP2C9 | Level 2 |  |  |
| DAAM1 | Level 2 |  |  |
| DAB2IP | Level 2 |  |  |
| DAG1 | Level 2 |  |  |
| DCAF7 | Level 2 |  |  |
| DDB2 | Level 2 |  |  |
| DDX19A | Level 2 |  |  |
| DDX19B | Level 2 |  |  |
| DDX42 | Level 2 |  |  |
| DGKZ | Level 2 |  |  |
| DHX38 | Level 2 |  |  |
| DNAJC5 | Level 2 |  |  |
| DNTTIP1 | Level 2 |  |  |
| DPP4 | Level 2 |  |  |
| ECM1 | Level 2 |  |  |
| EIF4A1 | Level 2 |  |  |
| EPOR | Level 2 |  |  |
| ERG | Level 2 |  |  |
| ERI1 | Level 2 |  |  |
| ESRRA | Level 2 |  |  |
| EXOSC6 | Level 2 |  |  |
| F12 | Level 2 |  |  |
| FAM167A | Level 2 |  |  |
| FBN2 | Level 2 |  |  |
| FBRS | Level 2 |  |  |
| FES | Level 2 |  |  |
| FGF11 | Level 2 |  |  |
| FNBP4 | Level 2 |  |  |
| FRK | Level 2 |  |  |
| FST | Level 2 |  |  |
| FUS | Level 2 |  |  |
| FXR2 | Level 2 |  |  |
| CASTOR1 | Level 2 |  |  |
| GCA | Level 2 |  |  |
| GFPT1 | Level 2 |  |  |
| GMPPB | Level 2 |  |  |
| GPX1 | Level 2 |  |  |
| GSN | Level 2 |  |  |
| HEATR6 | Level 2 |  |  |
| HELLS | Level 2 |  |  |
| HERPUD1 | Level 2 |  |  |
| HFE | Level 2 |  |  |
| H1-2 | Level 2 |  |  |
| H1-3 | Level 2 |  |  |
| H1-4 | Level 2 |  |  |
| H1-6 | Level 2 |  |  |
| H2AC6 | Level 2 |  |  |
| H2AC7 | Level 2 |  |  |
| H2AC8 | Level 2 |  |  |
| H2BC4 | Level 2 |  |  |
| H2BC5 | Level 2 |  |  |
| H2BC9 | Level 2 |  |  |
| H3C3 | Level 2 |  |  |
| H3C4 | Level 2 |  |  |
| H3C6 | Level 2 |  |  |
| H3C7 | Level 2 |  |  |
| H3C8 | Level 2 |  |  |
| H4C3 | Level 2 |  |  |
| H4C4 | Level 2 |  |  |
| H4C5 | Level 2 |  |  |
| H4C6 | Level 2 |  |  |
| H4C8 | Level 2 |  |  |
| HMGA1 | Level 2 |  |  |
| HOXB13 | Level 2 |  |  |
| HP | Level 2 |  |  |
| HSD3B7 | Level 2 |  |  |
| HSP90AB4P | Level 2 |  |  |
| IFIH1 | Level 2 |  |  |
| IGF2R | Level 2 |  |  |
| IL1F10 | Level 2 |  |  |
| IL1RN | Level 2 |  |  |
| IL36RN | Level 2 |  |  |
| IL6R | Level 2 |  |  |
| IP6K1 | Level 2 |  |  |
| IP6K2 | Level 2 |  |  |
| IPO9 | Level 2 |  |  |
| IREB2 | Level 2 |  |  |
| IRF1 | Level 2 |  |  |
| IRS1 | Level 2 |  |  |
| IST1 | Level 2 |  |  |
| JMJD1C | Level 2 |  |  |
| KANK2 | Level 2 |  |  |
| KAT8 | Level 2 |  |  |
| KCTD14 | Level 2 |  |  |
| KCTD9 | Level 2 |  |  |
| KDM2B | Level 2 |  |  |
| KDM4B | Level 2 |  |  |
| KDM6B | Level 2 |  |  |
| KLC1 | Level 2 |  |  |
| KLF3 | Level 2 |  |  |
| L3HYPDH | Level 2 |  |  |
| LAMB2 | Level 2 |  |  |
| LDAH | Level 2 |  |  |
| LDLR | Level 2 |  |  |
| LMAN2 | Level 2 |  |  |
| LPL | Level 2 |  |  |
| LRP1 | Level 2 |  |  |
| LRP4 | Level 2 |  |  |
| LSP1 | Level 2 |  |  |
| MACROD1 | Level 2 |  |  |
| MADD | Level 2 |  |  |
| MAP2K4 | Level 2 |  |  |
| MAP2K5 | Level 2 |  |  |
| MAP3K3 | Level 2 |  |  |
| MAP4 | Level 2 |  |  |
| MCF2L | Level 2 |  |  |
| ME2 | Level 2 |  |  |
| MEOX2 | Level 2 |  |  |
| MEX3C | Level 2 |  |  |
| MFHAS1 | Level 2 |  |  |
| MIA3 | Level 2 |  |  |
| MIR140 | Level 2 |  |  |
| MIR199A1 | Level 2 |  |  |
| MIR21 | Level 2 |  |  |
| MKLN1 | Level 2 |  |  |
| MPDU1 | Level 2 |  |  |
| MPLKIP | Level 2 |  |  |
| MRC2 | Level 2 |  |  |
| MSRA | Level 2 |  |  |
| MTCH2 | Level 2 |  |  |
| MTHFR | Level 2 |  |  |
| MYO1A | Level 2 |  |  |
| MYO9A | Level 2 |  |  |
| NAB2 | Level 2 |  |  |
| NCKIPSD | Level 2 |  |  |
| NCOA5 | Level 2 |  |  |
| NDUFA7 | Level 2 |  |  |
| NDUFS3 | Level 2 |  |  |
| NDUFS4 | Level 2 |  |  |
| NEFH | Level 2 |  |  |
| NF2 | Level 2 |  |  |
| NFAT5 | Level 2 |  |  |
| NIPSNAP1 | Level 2 |  |  |
| NLGN2 | Level 2 |  |  |
| NLRC5 | Level 2 |  |  |
| NOC3L | Level 2 |  |  |
| NOS3 | Level 2 |  |  |
| NPR3 | Level 2 |  |  |
| NRBF2 | Level 2 |  |  |
| NT5C2 | Level 2 |  |  |
| NUFIP2 | Level 2 |  |  |
| NUP160 | Level 2 |  |  |
| P4HA2 | Level 2 |  |  |
| P4HTM | Level 2 |  |  |
| PACSIN3 | Level 2 |  |  |
| PAFAH1B2 | Level 2 |  |  |
| PDGFRA | Level 2 |  |  |
| PECAM1 | Level 2 |  |  |
| PHLPP2 | Level 2 |  |  |
| PINX1 | Level 2 |  |  |
| PKHD1 | Level 2 |  |  |
| PKM | Level 2 |  |  |
| PLAU | Level 2 |  |  |
| PLCB3 | Level 2 |  |  |
| PLCE1 | Level 2 |  |  |
| PLG | Level 2 |  |  |
| PLXNB1 | Level 2 |  |  |
| POLR2A | Level 2 |  |  |
| PRDX5 | Level 2 |  |  |
| PRELID1 | Level 2 |  |  |
| PRKAR2A | Level 2 |  |  |
| PRPF3 | Level 2 |  |  |
| PSMA4 | Level 2 |  |  |
| PSMC3 | Level 2 |  |  |
| PTPMT1 | Level 2 |  |  |
| PTPRJ | Level 2 |  |  |
| PTRH2 | Level 2 |  |  |
| NECTIN2 | Level 2 |  |  |
| PYCARD | Level 2 |  |  |
| QARS1 | Level 2 |  |  |
| QRICH1 | Level 2 |  |  |
| RAB24 | Level 2 |  |  |
| RDX | Level 2 |  |  |
| REEP3 | Level 2 |  |  |
| RGS14 | Level 2 |  |  |
| RHBDD1 | Level 2 |  |  |
| RHOA | Level 2 |  |  |
| RNF123 | Level 2 |  |  |
| RNF34 | Level 2 |  |  |
| RPRD2 | Level 2 |  |  |
| RPS28 | Level 2 |  |  |
| RPS6KA4 | Level 2 |  |  |
| RPS6KB1 | Level 2 |  |  |
| RRP15 | Level 2 |  |  |
| RSF1 | Level 2 |  |  |
| SARS2 | Level 2 |  |  |
| SCARB1 | Level 2 |  |  |
| SDCCAG8 | Level 2 |  |  |
| SDR9C7 | Level 2 |  |  |
| SENP3 | Level 2 |  |  |
| SENP8 | Level 2 |  |  |
| SERTAD4 | Level 2 |  |  |
| SF3A1 | Level 2 |  |  |
| SF3A2 | Level 2 |  |  |
| SIDT2 | Level 2 |  |  |
| SIK3 | Level 2 |  |  |
| SLC22A3 | Level 2 |  |  |
| SLC22A4 | Level 2 |  |  |
| SLC25A20 | Level 2 |  |  |
| SLC26A6 | Level 2 |  |  |
| SLC39A1 | Level 2 |  |  |
| SMAD4 | Level 2 |  |  |
| SMARCA4 | Level 2 |  |  |
| SMARCD1 | Level 2 |  |  |
| SMOC1 | Level 2 |  |  |
| SMYD2 | Level 2 |  |  |
| SNRPC | Level 2 |  |  |
| SNX32 | Level 2 |  |  |
| SORT1 | Level 2 |  |  |
| SOX6 | Level 2 |  |  |
| SPC24 | Level 2 |  |  |
| SPI1 | Level 2 |  |  |
| SPP1 | Level 2 |  |  |
| SPSB1 | Level 2 |  |  |
| SPTBN4 | Level 2 |  |  |
| SREBF1 | Level 2 |  |  |
| STAG3L1 | Level 2 |  |  |
| STAT6 | Level 2 |  |  |
| STX1B | Level 2 |  |  |
| STX4 | Level 2 |  |  |
| TARS2 | Level 2 |  |  |
| TGFBRAP1 | Level 2 |  |  |
| THADA | Level 2 |  |  |
| THOC5 | Level 2 |  |  |
| TLK2 | Level 2 |  |  |
| TMED1 | Level 2 |  |  |
| TMEM102 | Level 2 |  |  |
| TNFSF12 | Level 2 |  |  |
| TNFSF13 | Level 2 |  |  |
| TNNC2 | Level 2 |  |  |
| TNNT3 | Level 2 |  |  |
| TOM1L2 | Level 2 |  |  |
| TOMM40 | Level 2 |  |  |
| TP53 | Level 2 |  |  |
| TPD52L2 | Level 2 |  |  |
| TRMT112 | Level 2 |  |  |
| TTC28 | Level 2 |  |  |
| TXNL4B | Level 2 |  |  |
| UBE2C | Level 2 |  |  |
| UBE2Q1 | Level 2 |  |  |
| UCKL1 | Level 2 |  |  |
| UHRF1 | Level 2 |  |  |
| USP19 | Level 2 |  |  |
| USP3 | Level 2 |  |  |
| USP4 | Level 2 |  |  |
| VEGFA | Level 2 |  |  |
| VKORC1 | Level 2 |  |  |
| VMP1 | Level 2 |  |  |
| WAPL | Level 2 |  |  |
| WDR6 | Level 2 |  |  |
| WRAP53 | Level 2 |  |  |
| WWP2 | Level 2 |  |  |
| XRCC3 | Level 2 |  |  |
| ZBTB46 | Level 2 |  |  |
| ZBTB7A | Level 2 |  |  |
| ZC3HC1 | Level 2 |  |  |
| ZFYVE21 | Level 2 |  |  |
| ZKSCAN1 | Level 2 |  |  |
| ZNF589 | Level 2 |  |  |
| ZNF646 | Level 2 |  |  |
| ZNF668 | Level 2 |  |  |
| ZPR1 | Level 2 |  |  |
| ZSWIM1 | Level 2 |  |  |
| ZSWIM3 | Level 2 |  |  |
| RAC1 | Bridging |  |  |
| SQSTM1 | Bridging |  |  |
| PLEKHA4 | Bridging |  |  |
| RNF4 | Bridging |  |  |
| INS | Bridging |  |  |
| CD93 | Bridging |  |  |
| HNRNPL | Bridging |  |  |
| CISH | Bridging |  |  |
| IRAK1 | Bridging |  |  |
| TRAF6 | Bridging |  |  |
| TRIM28 | Bridging |  |  |
| BST2 | Bridging |  |  |
| PFKL | Bridging |  |  |
| SPOP | Bridging |  |  |
| ZNF35 | Bridging |  |  |
| MDM2 | Bridging |  |  |
| PRDX1 | Bridging |  |  |
| TRAF2 | Bridging |  |  |
| TSG101 | Bridging |  |  |
| MAPK14 | Bridging |  |  |
| ATG5 | Bridging |  |  |
| IRF4 | Bridging |  |  |
| AKT1 | Bridging |  |  |
| MAP3K1 | Bridging |  |  |
| CD14 | Bridging |  |  |
| BANK1 | Bridging |  |  |
| TRAF3 | Bridging |  |  |
| TLR2 | Bridging |  |  |
| STAP2 | Bridging |  |  |
| TLR3 | Bridging |  |  |
| POLR1C | Bridging |  |  |
| SMAD3 | Bridging |  |  |
| TIRAP | Bridging |  |  |
| FLII | Bridging |  |  |
| CARD9 | Bridging |  |  |
| MAP4K4 | Bridging |  |  |
| IKBKB | Bridging |  |  |
| TXN | Bridging |  |  |
| HDAC6 | Bridging |  |  |
| IKBKG | Bridging |  |  |
| RIPK2 | Bridging |  |  |
| MAL | Bridging |  |  |
| USP7 | Bridging |  |  |
| TNIP1 | Bridging |  |  |
| NGFR | Bridging |  |  |
| SIAH1 | Bridging |  |  |
| IKBKE | Bridging |  |  |
| TNFRSF1A | Bridging |  |  |
| SARM1 | Bridging |  |  |
| CYLD | Bridging |  |  |
| DOCK8 | Bridging |  |  |
| SMURF1 | Bridging |  |  |
| PTK2B | Bridging |  |  |
| MAP3K7 | Bridging |  |  |
| NFKBIA | Bridging |  |  |
| TLR4 | Bridging |  |  |
| IL1RL1 | Bridging |  |  |
| WDYHV1 | Bridging |  |  |
| CAMLG | Bridging |  |  |
| BTK | Bridging |  |  |
| LRRFIP2 | Bridging |  |  |
| IRAK3 | Bridging |  |  |
| SYK | Bridging |  |  |
| CBLB | Bridging |  |  |
| MBIP | Bridging |  |  |
| TBK1 | Bridging |  |  |
| GLOD4 | Bridging |  |  |
| IL1R1 | Bridging |  |  |
| IRAK4 | Bridging |  |  |
| CYTH2 | Bridging |  |  |
| PELI1 | Bridging |  |  |
| TLR9 | Bridging |  |  |
| TLR10 | Bridging |  |  |
| FADD | Bridging |  |  |
| IL1B | Bridging |  |  |
| SIAH2 | Bridging |  |  |
| TLR5 | Bridging |  |  |
| CASP1 | Bridging |  |  |
| IRF7 | Bridging |  |  |
| AGER | Bridging |  |  |
| TLR7 | Bridging |  |  |
| TNFRSF13B | Bridging |  |  |
| IL1RAP | Bridging |  |  |
| SMAD6 | Bridging |  |  |
| NOD2 | Bridging |  |  |
| PELI2 | Bridging |  |  |
| LRRFIP1 | Bridging |  |  |
| HFE | Level 2 | Zucker, 2023 | 37133573 |
| SLC4A7 | Level 2 |  |  |
| SWAP70 | Level 2 |  |  |
| SIPA1 | Level 2 |  |  |
| LSP1 | Level 2 |  |  |
| SLC22A4 | Level 2 |  |  |
| BAG6 | Level 2 |  |  |
| ZNF738 | Level 2 |  |  |
| PHETA1 | Level 2 |  |  |
| NPC1 | Level 2 |  |  |
| INPP5A | Level 2 |  |  |
| PTPRJ | Level 2 |  |  |
| IMMT | Level 2 |  |  |
| ZNF100 | Level 2 |  |  |
| SPCS1 | Level 2 |  |  |
| SMG6 | Level 2 |  |  |
| ZC3HC1 | Level 2 |  |  |
| GNL3 | Level 2 |  |  |
| ACP2 | Level 2 |  |  |
| RRP1B | Level 2 |  |  |
| PLCE1 | Level 2 |  |  |
| SLC16A1 | Level 2 |  |  |
| NOS3 | Level 2 |  |  |
| NOTCH4 | Level 2 |  |  |
| NGF | Level 2 |  |  |
| FNBP4 | Level 2 |  |  |
| CPA4 | Level 2 |  |  |
| PLCB3 | Level 2 |  |  |
| NUP160 | Level 2 |  |  |
| LTA | Level 2 |  |  |
| TBCK | Level 2 |  |  |
| SQSTM1 | Bridging |  |  |
| RAC1 | Bridging |  |  |
| RNF4 | Bridging |  |  |
| HDAC6 | Bridging |  |  |
| MDM2 | Bridging |  |  |
| TRIM28 | Bridging |  |  |
| TSG101 | Bridging |  |  |
| USP7 | Bridging |  |  |
| IKBKE | Bridging |  |  |
| CD93 | Bridging |  |  |
| CARD9 | Bridging |  |  |
| LRRFIP2 | Bridging |  |  |
| TLR3 | Bridging |  |  |
| PLEKHA4 | Bridging |  |  |
| HNRNPL | Bridging |  |  |
| IRAK1 | Bridging |  |  |
| PFKL | Bridging |  |  |
| SYK | Bridging |  |  |
| MAPK14 | Bridging |  |  |
| SIAH2 | Bridging |  |  |
| SIAH1 | Bridging |  |  |
| TRAF6 | Bridging |  |  |
| AKT1 | Bridging |  |  |
| SMAD3 | Bridging |  |  |
| NGFR | Bridging |  |  |
| CYLD | Bridging |  |  |
| TNFRSF1A | Bridging |  |  |

Supplementary Table 2: Association of MyD88 and CD14 mRNA expression with markers of endothelial function and vascular stiffness. Linear regression models, adjusted for storage time, rin number and plate layout. A: Association with flow mediated dilation (FMD). B: Association with stiffness index (SI)

**A:**

|  | **Estimate** | **L95%CI** | **U95%CI** | **p-value** |
| --- | --- | --- | --- | --- |
| N: 1,245 |  |  |  |  |
| Sex (women) | 3.27 | 2.72 | 3.81 | <0.0001 |
| Age [y] | -0.102 | -0.127 | -0.0778 | <0.0001 |
| MYD88 [MYD88>10.7] | -0.797 | -1.82 | 0.223 | 0.13 |
| CD14 [per SD] | 0.0853 | -0.201 | 0.372 | 0.56 |

**B:**

|  | **Estimate** | **L95%CI** | **U95%CI** | **p-value** |
| --- | --- | --- | --- | --- |
| N: 1,153 |  |  |  |  |
| Sex (women) | -1.93 | -2.24 | -1.63 | <0.0001 |
| Age [y] | 0.0867 | 0.0728 | 0.101 | <0.0001 |
| MYD88 [MYD88>10.7] | 0.124 | -0.445 | 0.694 | 0.67 |
| CD14 [per SD] | -0.115 | -0.273 | 0.0437 | 0.16 |

**Supplementary Table 3: MyD88 single nucleotide polymorhpisms, MyD88 mRNA expression and systolic blood pressure**. Linear regression: estimated effects of Myd88 SNPs (A) and MyD88 mRNA Expression (B) on prevalent systolic blood pressure in 4160 individuals of the GHS, adjusted for sex, age, diabetes, obesity, smoking, dyslipidemia, family history of MI/Stroke and medication. P-values (P< 0.05) are highlighted in bold.

**A:**

|  | **Estimate** | **L95%CI** | **U95%CI** | **p-value** |
| --- | --- | --- | --- | --- |
| N: 4160 |  |  |  |  |
| Sex (women) | -5.01 | -5.98 | -4.04 | **<0.0001** |
| Age [y] | 0.575 | 0.526 | 0.623 | **<0.0001** |
| rs7744 (per G allel) | -0.687 | -1.62 | 0.249 | 0.15 |
| rs4988453 (per A allel) | 0.455 | -1.12 | 2,03 | 0.57 |
| Antihypertensives (c02) | 3,25 | -1.47 | 7,97 | 0.18 |
| Diuretics (c03) | -3.94 | -6.11 | -1.77 | **0.00038** |
| Beta-blockers (c07) | 1,28 | -0.151 | 2,70 | 0.080 |
| Calcium channel blocker (c08) | 2,41 | 0.417 | 4,41 | **0.018** |

**B:**

|  | **Estimate** | | | **L95%CI** | **U95%CI** | | | **p-value** |
| --- | --- | --- | --- | --- | --- | --- | --- | --- |
| N: 1261 | | **Estimate** | **L95%CI** | | | **U95%CI** | **p-value** | |
| Sex (women) | | -6.13 | -7.90 | | | -4.35 | **<0.0001** | |
| Age [y] | | 0.536 | 0.445 | | | 0.627 | **<0.0001** | |
| MYD88 [MYD88>10.7] | | -2.27 | -5.55 | | | 1.01 | 0.18 | |
| Antihypertensives (c02) | | -3.42 | -7.71 | | | 0.864 | 0.12 | |
| Diuretics (c03) | | 1.18 | -1.45 | | | 3.81 | 0.38 | |
| Beta-blockers (c07) | | 0.353 | -3.58 | | | 4.29 | 0.86 | |
| Calcium channel blocker (c08) | | 3.88 | 1.41 | | | 6.35 | **0.0022** | |

**Supplementary Table 4:** Cohort characteristics of participants from GHS grouped by low (<=10.7) and high expression (>10.7) of MyD88. P-values (P< 0.05) are highlighted in bold.

|  | **All (1274)** | **MYD88<=10.7 (1151)** | **MYD88>10.7 (123)** | **p** |
| --- | --- | --- | --- | --- |
| Sex (Women) | 618 (48.5%) | 564 (49.0%) | 54 (43.9%) | 0.30 |
| Age [y] | 54.7(11.1 SD) | 54.5(11.1 SD) | 56.7(10.8 SD) | **0.032** |
| **CVRFs:** |  |  |  |  |
| Diabetes (yes) | 119 (9.4%) | 103 (9.0%) | 16 (13.0%) | 0.14 |
| Obesity (yes) | 290 (22.8%) | 252 (21.9%) | 38 (30.9%) | **0.031** |
| Smoking (yes) | 236 (18.6%) | 213 (18.5%) | 23 (18.9%) | 0.90 |
| Hypertension (yes) | 628 (49.3%) | 562 (48.8%) | 66 (53.7%) | 0.34 |
| Dyslipidemia (yes) | 567 (44.6%) | 509 (44.2%) | 58 (47.9%) | 0.44 |
| FH of MI/Stroke (yes) | 290 (22.8%) | 260 (22.6%) | 30 (24.4%) | 0.65 |
| **CVRFs (continous variable):** |  |  |  |  |
| BMI [kg/m²] | 27.0(4.6 SD) | 26.9(4.6 SD) | 28.0(4.6 SD) | **0.014** |
| Height [cm] | 171(9 SD) | 171(9 SD) | 171(8 SD) | 0.97 |
| WHtR | 0.547(0.077 SD) | 0.545(0.076 SD) | 0.565(0.076 SD) | **0.0061** |
| SBP [mmHg] | 132(18 SD) | 132(18 SD) | 132(15 SD) | 0.90 |
| DBP [mmHg] | 83.5(9.7 SD) | 83.5(9.7 SD) | 83.8(9.5 SD) | 0.76 |
| HR [bpm] | 68.7(11.0 SD) | 68.8(11.0 SD) | 67.7(10.3 SD) | 0.28 |
| Cholesterol [mmol/l] | 224(43 SD) | 224(43 SD) | 218(38 SD) | 0.084 |
| HDL [mg/dl] | 56.7(15.5 SD) | 56.9(15.5 SD) | 54.6(15.6 SD) | 0.12 |
| LDL [mg/dl] | 141(36 SD) | 142(36 SD) | 137(34 SD) | 0.14 |
| LDL/HDL | 2.65(0.95 SD) | 2.65(0.95 SD) | 2.68(0.93 SD) | 0.77 |
| Triglycerides [mg/dl] | 107.4 (80.4, 153.4) | 107.0 (80.1, 151.4) | 109.7 (82.4, 168.3) | 0.38 |
| HbA1c [%] | 5.40 (5.10, 5.70) | 5.30 (5.00, 5.70) | 5.40 (5.20, 5.90) | **0.0049** |
| **Diseases:** |  |  |  |  |
| MI (yes) | 26 (2.0%) | 24 (2.1%) | 2 (1.6%) | 1.00 |
| Stroke (yes) | 21 (1.7%) | 18 (1.6%) | 3 (2.5%) | 0.45 |
| AF (yes) | 40 (3.2%) | 34 (3.0%) | 6 (5.0%) | 0.27 |
| PAD (yes) | 60 (4.8%) | 51 (4.5%) | 9 (7.4%) | 0.17 |
| CAD (yes) | 58 (4.6%) | 50 (4.4%) | 8 (6.6%) | 0.25 |
| CHF (yes) | 17 (1.3%) | 12 (1.0%) | 5 (4.1%) | **0.018** |
| DVT (yes) | 48 (3.8%) | 39 (3.4%) | 9 (7.4%) | **0.042** |
| PE (yes) | 1 (0.1%) | 1 (0.1%) | 0 (0%) | 1.00 |
| COPD (yes) | 51 (4.0%) | 46 (4.0%) | 5 (4.1%) | 1.00 |
| CKD (yes) | 59 (4.6%) | 50 (4.3%) | 9 (7.4%) | 0.17 |
| CLD (yes) | 13 (1.0%) | 12 (1.0%) | 1 (0.8%) | 1.00 |
| Cancer (yes) | 115 (9.0%) | 99 (8.6%) | 16 (13.1%) | 0.099 |
| acute infection (yes) | 293 (23.3%) | 274 (24.1%) | 19 (15.7%) | **0.041** |
| **Endothelial and vascular function:** |  |  |  |  |
| baseline RI | 69.0 (57.0, 78.0) | 69.0 (57.0, 78.0) | 73.0 (58.0, 80.3) | **0.046** |
| hyperemic RI | 68.0 (53.0, 78.0) | 67.0 (53.0, 77.0) | 71.0 (57.0, 80.0) | 0.065 |
| RI difference | 2.00 (-4.00, 9.00) | 2.00 (-4.00, 9.00) | 1.00 (-5.33, 9.00) | 0.53 |
| fRHI | 0.656 (0.348, 0.915) | 0.665 (0.357, 0.918) | 0.595 (0.236, 0.874) | 0.11 |
| Mean BL pre FMD [mm] | 4.35 (3.66, 4.96) | 4.36 (3.65, 4.96) | 4.20 (3.80, 4.98) | 0.67 |
| FMD [%] | 7.43 (4.76, 10.89) | 7.44 (4.80, 11.06) | 7.28 (4.28, 10.17) | 0.14 |
| SI [m/s] | 8.59 (6.50, 11.21) | 8.50 (6.45, 11.14) | 9.71 (6.97, 11.88) | **0.018** |
| ABI | 0.963(0.132 SD) | 0.962(0.133 SD) | 0.968(0.124 SD) | 0.64 |
| IMT [mm] | 0.642(0.127 SD) | 0.639(0.126 SD) | 0.668(0.132 SD) | **0.022** |
| incident Hypertension (yes) | 115 (20.2%) | 105 (20.3%) | 10 (19.2%) | 1.00 |
| Heart failure (HF) (yes) | 61 (5.0%) | 56 (5.0%) | 5 (4.5%) | 1.00 |
| Diastolic dysfunction (DDo) (yes) | 225 (20.0%) | 197 (19.3%) | 28 (27.2%) | 0.070 |
| **Genetic:** |  |  |  |  |
| MYD88 | 10.22(0.33 SD) | 10.15(0.27 SD) | 10.84(0.15 SD) | **<0.0001** |
| CD14 | 14.5(0.1 SD) | 14.5(0.1 SD) | 14.5(0.2 SD) | **<0.0001** |
| storage.time | 314(92 SD) | 313(90 SD) | 330(107 SD) | 0.091 |
| RIN | 9.36(0.43 SD) | 9.36(0.43 SD) | 9.37(0.44 SD) | 0.81 |
| **rs7744** |  |  |  |  |
| - A/A | 780 (69.5%) | 701 (69.3%) | 79 (71.2%) |  |
| - A/G | 318 (28.3%) | 287 (28.4%) | 31 (27.9%) |  |
| - G/G | 24 (2.1%) | 23 (2.3%) | 1 (0.9%) |  |
| **rs4988453** |  |  |  |  |
| - C/C | 1008 (89.8%) | 909 (89.9%) | 99 (89.2%) |  |
| - C/A | 110 (9.8%) | 99 (9.8%) | 11 (9.9%) |  |
| - A/A | 4 (0.4%) | 3 (0.3%) | 1 (0.9%) |  |

**Supplementary Table 5**: Systolic blood pressure values measured at baseline (grey) for 1 week and subsequently measured SBP after Angiotensin II infusion (orange).

|  | Systolic blood pressure recording [h] | C57BL/6 | | | | | MyD88 ^-/-^ | | | | |
| --- | --- | --- | --- | --- | --- | --- | --- | --- | --- | --- | --- |
|  |  | #1 | #2 | #3 | #4 | #5 | #1 | #2 | #3 | #4 | #5 |
| Baseline | 24 | 119,8 | 104,9 | 111,1 | 105,4 | 104,9 | 108,7 | 112,6 | 116,3 | 107,5 | 119,0 |
|  | 48 | 119,3 | 101,7 | 111,1 | 105,3 | 106,4 | 108,5 | 111,1 | 113,3 | 106,9 | 118,2 |
|  | 72 | 121,3 | 100,8 | 110,4 | 107,0 | 106,6 | 106,2 | 106,1 | 108,8 | 106,5 | 118,6 |
|  | 96 | 119,3 | 100,6 | 112,3 | 108,7 | 106,2 | 103,6 | 106,9 | 110,2 | 102,9 | 121,4 |
|  | 120 | 124,6 | 99,4 | 111,4 | 108,2 | 110,1 | 109,5 | 106,7 | 109,5 | 106,9 | 118,2 |
|  | 144 | 120,4 | 102,5 | 110,7 | 107,4 | 110,1 | 106,5 | 110,6 | 116,9 | 109,6 | 116,5 |
|  | 168 | 118,0 | 101,9 | 114,0 | 109,1 | 112,3 | 103,5 | 111,0 | 113,8 | 105,9 | 115,0 |
| AngII infusion | 192 | 123,2 | 124,1 | 140,6 | 138,9 | 134,2 | 103,4 | 113,8 | 112,2 | 130,2 | 142,7 |
|  | 216 | 112,9 | 120,1 | 137,7 | 140,9 | 145,2 | 118,4 | 127,5 | 127,1 | 114,7 | 139,8 |
|  | 240 | 154,7 | 117,2 | 130,0 | 140,7 | 147,0 | 134,9 | 129,4 | 127,4 | 108,5 | 116,6 |
|  | 264 | 157,1 | 122,1 | 134,6 | 136,6 | 146,2 | 126,8 | 130,4 | 130,4 | 105,7 | 112,3 |
|  | 288 | 148,3 | 128,7 | 146,6 | 145,8 | 149,2 | 131,5 | 131,6 | 133,3 | 106,5 | 132,1 |
|  | 312 | 142,1 | 136,6 | 151,4 | 146,2 | 154,7 | 133,8 | 129,6 | 128,9 | 103,9 | 139,1 |
|  | 336 | 144,9 | 137,9 | 148,7 | 155,1 | 159,9 | 133,4 | 127,3 | 133,1 | 114,5 | 142,5 |

**Supplementary Table 6**: **Interaction of anti-hypersensitive medication in GHS patients with MyD88>10.7 to all-cause death.** A: Anti-hypersensitive medication in patients with low (MyD88<=10.7) and high (MyD88>10.7) MyD88 expression. B: Cox regression: estimated effects of high MyD88 expression (MyD88>10.7) on all-cause mortality in 1274 individuals with anti-hypertensive treatment from the GHS, adjusted for sex and age. P-values (P< 0.05) are highlighted in bold.

**A:**

| **Medication:** | **All (1274)** | **MYD88<=10.7 (1151)** | **MYD88>10.7 (123)** |
| --- | --- | --- | --- |
| ACE Inhibitors: C09A (gesamt) (yes) | 119 (9.4%) | 107 (9.3%) | 12 (9.8%) |
| Angiotensin II receptor blockers (ARBs): C09C (gesamt) (yes) | 42 (3.3%) | 39 (3.4%) | 3 (2.4%) |
| Calcium channel blockers. C08 (yes) | 80 (6.3%) | 72 (6.3%) | 8 (6.5%) |
| Beta blocking agents C07 (yes) | 203 (16.0%) | 177 (15.5%) | 26 (21.1%) |
| Aldosteron Anatagonisten: C03D (yes) | 1 (0.1%) | 1 (0.1%) | 0 (0%) |
| Diuretrics (Thiazids) C03A und C03B (yes) | 22 (1.7%) | 19 (1.7%) | 3 (2.4%) |
| Furosemid C03C (yes) | 22 (1.7%) | 18 (1.6%) | 4 (3.3%) |

**B:**

|  | **Sample** | **HR** | **L95%CI** | **U95%CI** | **p-value** | **C-Index** |
| --- | --- | --- | --- | --- | --- | --- |
| Event~ | N:1274 (178 events) |  |  |  |  | 0.822410 |
| Sex (Women) |  | 0.5226 | 0.3824 | 0.7141 | **<0.0001** | 0.00724 |
| Age [5y] |  | 19.314 | 17.468 | 21.355 | **<0.0001** | 0.22733 |
| MYD88 [>10.7] |  | 12.624 | 0.8253 | 19.312 | 0.28 | 0.00074 |
|  | **Sample** | **HR** | **L95%CI** | **U95%CI** | **p-value** | **C-Index** |
| Event~ | N:1268 (178 events) |  |  |  |  | 0.822381 |
| Sex (Women) |  | 0.5237 | 0.3832 | 0.7157 | **<0.0001** | 0.00765 |
| Age [5y] |  | 19.213 | 17.350 | 21.276 | **<0.0001** | 0.20353 |
| MYD88 [>10.7] |  | 13.389 | 0.8340 | 21.497 | 0.23 | 0.00084 |
| C09A * MYD88>10.7 |  | 0.7193 | 0.2472 | 20.929 | 0.55 | 0.00011 |
| C09A |  | 12.432 | 0.8184 | 18.884 | 0.31 | 0.00070 |
|  | **Sample** | **HR** | **L95%CI** | **U95%CI** | **p-value** | **C-Index** |
| Event~ | N:1268 (178 events) |  |  |  |  | 0.825673 |
| Sex (Women) |  | 0.5225 | 0.3816 | 0.7154 | **<0.0001** | 0.00781 |
| Age [5y] |  | 19.144 | 17.278 | 21.211 | **<0.0001** | 0.18382 |
| MYD88 [>10.7] |  | 0.9650 | 0.5425 | 17.167 | 0.90 | -0.00015 |
| C07 * MYD88>10.7 |  | 18.912 | 0.7971 | 44.870 | 0.15 | 0.00037 |
| C09A |  | 13.605 | 0.9542 | 19.398 | 0.089 | 0.00192 |
|  | **Sample** | **HR** | **L95%CI** | **U95%CI** | **p-value** | **C-Index** |
| Event~ | N:1268 (178 events) |  |  |  |  | 0.824737 |
| Sex (Women) |  | 0.5259 | 0.3848 | 0.7187 | **<0.0001** | 0.00731 |
| Age [5y] |  | 18.998 | 17.163 | 21.029 | **<0.0001** | 0.19447 |
| MYD88 [>10.7] |  | 0.7702 | 0.3772 | 15.730 | 0.47 | 0.00038 |
| Hyp.Rx * MYD88>10.7 |  | 23.379 | 0.9844 | 55.520 | 0.054 | 0.00247 |
| C09A |  | 10.757 | 0.7206 | 16.058 | 0.72 | 0.00037 |
